# Instruments for assessing social health in the context of cognitive decline and dementia: a systematic review

**DOI:** 10.3389/fpsyt.2024.1387192

**Published:** 2024-11-13

**Authors:** Janissa Altona, Henrik Wiegelmann, Marta Lenart-Bulga, Myrra Vernooij-Dassen, Eline Verspoor, Imke Seifert, Julia Misonow, Dorota Szcześniak, Joanna Rymaszewska, Rabih Chattat, Yun-Hee Jeon, Esme Moniz-Cook, Martina Roes, Marieke Perry, Karin Wolf-Ostermann

**Affiliations:** ^1^ Department of Nursing Science Research, Institute of Public Health and Nursing Research, University of Bremen, Bremen, Germany; ^2^ Department of Psychiatry, Wroclaw Medical University, Wroclaw, Poland; ^3^ Institute for Health Sciences Radboud, Radboudumc Alzheimer Center, Radboud University Medical Center, Nijmegen, Netherlands; ^4^ Department of Clinical Neuroscience, Wroclaw University of Science and Technology, Wroclaw, Poland; ^5^ Department of Psychology, Alma Mater Studiorum, University of Bologna, Bologna, Italy; ^6^ Susan Wakil School of Nursing and Midwifery, The University of Sydney, Sydney, NSW, Australia; ^7^ Faculty of Health Sciences, University of Hull, Hull, United Kingdom; ^8^ German Center for Neurodegenerative Diseases e. V. (DZNE), site Witten, Department of Nursing Science, Faculty of Health, University of Witten/Herdecke, Witten, Germany; ^9^ Department of Primary and Community Care, Radboud University Medical Center, Nijmegen, Netherlands

**Keywords:** social health, social factors, dementia, cognitive decline, measures

## Abstract

The concept of social health has recently received increasing attention in dementia research. Various notions of what social health is and how it can be measured are circulating. They may pose challenges for comparing results and interpreting them for the development of interventions. This systematic review aims to classify existing instruments that measure various domains of social health. To achieve this, we applied a new multidimensional framework consisting of six key domains of social health. A systematic review was conducted following the PRISMA 2020 guidelines. PubMed/MEDLINE, PsychINFO, and CINAHL were searched for studies published between January 2000 and July 2023. A total of 227 studies (longitudinal, case–control, and cross-sectional cohort studies) with 102 single instruments were included. The search terms were as follows: (1) dementia (i.e., Alzheimer’s, cognitive impairment); (2) social health markers (i.e., decision-making, social participation, loneliness); and (3) instruments (i.e., tools, measures). The instruments are mainly self-reported, and the number of items ranges from 3 to 126. Despite the wide array of instruments available, most focus on individual domains of social health. We recommend the development of more conceptually robust instruments that can comprehensively evaluate psychosocial interventions and adequately capture all domains of social health.

## 1 Introduction

Social health as a concept emerged within the context of a critique of a narrow understanding of health, recognized as a steady state of complete physical, mental, and social well-being (1). Huber and colleagues (2) therefore re-conceptualized health as the “ability to adapt and self-manage”, pointing to a more dynamic nature and consideration of chronic diseases. Social health was described as a dynamic balance between individual capacities and the limits set by one’s social environment. The individual capacities refer to the three abilities: (1) to fulfill potential and obligations on a societal level, (2) to manage life with some degree of independence despite a medical condition, and (3) to participate in social activities including work.

Meaningful social relationships are crucial for healthy aging, contributing to lower mortality rates and better physical and mental health outcomes (3). Social connections, engagement, and participation in social activities are critical dimensions of social health, potentially buffering against cognitive decline by delaying or even protecting against the onset of dementia (4, 5).

With increasing knowledge of the role of social factors in the prevention, development, and intervention of dementia research in the past two decades, the need to refine and adapt the concept of social health for this specific population has gained importance (6–9). Valtorta et al. (10) note the continued lack of clarity of terminology and constructs surrounding social relationships, social networks, and types of social support (11, 12). Therefore, in response to Huber’s new social health definition, Dröes et al. (13) operationalized the three individual domains for people with dementia, emphasizing the limitations and possibilities arising from the condition. These are as follows: (1) the ability of a person living with dementia to function in society according to his or her competencies and talents (potentials) in the best possible way, and to meet social demands (obligations) on a micro and macro-societal level, (2) the ability of the person with dementia to preserve autonomy and to solve problems in daily life, as well as to adapt to and cope with the practical and emotional consequences, (3) the act of being occupied or involved in meaningful activities, social interactions, and social ties and relationships which are meaningful to the person living with dementia (PlwD).

Earlier, somewhat independently of the forging of the concept of social health, Berkman and colleagues (14) created a social network model, linking the macro and meso social environment with the health of an individual. This model includes not only structural and functional network elements but also indicates their connection with the so-called psychosocial mechanisms, i.e. social support, social influence, social engagement, person-to-person contact, and access to resources and material goods. Already then, the authors indicated interchangeably used terms of social activity, social participation, social interaction, or social engagement.

These two, so to say, independently existing approaches to social relations (subjective-individual and objective-social network) not only demonstrate the risk of mixing similar concepts (i.e., social participation and frequency of contacts) and difficulties in comparing studies, but they may have implications for understanding health outcomes. For example, studies using objective and subjective social measurements found differing impacts on health (15–17). A comprehensive systematic review of randomized controlled trials (RCTs), longitudinal, and genetic studies (18) already pointed to the problem of heterogeneity of definitions and measures of various social factors, which contributes to the difficulty in collecting scientific evidence and making comparisons between results.

Currently, most of the existing studies investigate the relationship between cognitive health and separate social health factors, i.e. social support, social engagement, social network size, social participation, and social isolation, during the entire trajectory from cognitive health to severe dementia (5, 18–20). More recently, researchers have used collective indexes, such as the social network index (with network size and social support as two standardized indices) to conduct longitudinal work on the risk of dementia and brain volume (21). The lack of a systematized concept of social health led to a natural consequence of the heterogeneity of the operationalization of individual social constructs.

The first classification of tools measuring social relations was undertaken by Valtorta et al. (10), in which instruments were classified based on two dimensions: (1) structural and functional aspects of social relations, and (2) the degree of subjectivism attributed to respondents. However, this work is a fair description and comparison of the 54 tools. A few years later, Siette et al. (2021) (22) still pointing to the overall disagreement over the definition and theoretical basis of social networks, conducted a comprehensive overview, including 229 studies with 21 instruments measuring social networks for older adults, and distinguished three dimensions: quantitative, qualitative, and alter members (specific ties to other people, e.g., family members or neighbors). Thus, the authors tackled the multidimensionality of the social network concept, which was once assessed mainly in terms of structural aspects. However, in the last decade, social networks are also measured in terms of accessibility and the social support received from them. Siette et al. (2021) also found that this heterogeneity forced the understanding of the social network as an “umbrella” concept that includes various concepts, such as structure, support, and even a subjective assessment of the quality of social network contacts (22).

A different approach to social health—building on Huber’s conceptual work (4)—was presented by Mangiaracina et al. (23), leaving the social network and focusing on the individual dimension of social functioning. They have provided an overview of available instruments for the assessment of two domains, previously described by Dröes et al. (13) for the dementia population: the ability to manage life with some degree of independence and participation in social activities. The authors identified eight instruments for self-management and three for participation in social activities applied in community-dwelling people with mild dementia, bringing up the need for feasibility, better psychometrics, and developing tools covering the quality of social interactions. However, in a more recent systematic review, Grothe et al. (24) emphasized the need to differentiate social functioning from similar terms, such as social participation, social activities, or social engagement as well as social network. As a result of an extensive review of the literature, researchers identified three tools for measuring social functioning in people with dementia, while pointing to the importance of relationship changes in the dementia process and their assessment by both dyadic actors, the persons with dementia, and main informal caregivers.

In the light of various approaches to social health in the context of dementia, which includes both the assessment of the role of the individual person and the role of the social environment, it becomes more and more justified to understand this area of health as a result of the interaction of at least two participants. Particularly, for people with dementia where cognitive decline can put pressure on the person’s functioning, the social environment becomes equally important for the person’s social health (25). Hence, the original concept has been reconceived to add the environment level, relating to the characteristics of the person’s immediate social environment (26). Social health is understood as a reciprocal relational concept in which well-being is defined by how an individual relates to the social environment and how the social environment relates to the individual. Such an approach emphasizes the role of a person’s interaction with the social world and points to shared responsibility for the overall picture of social health (26). In this article, therefore, we refer to the most recent and comprehensive understanding of the social health of Vernooij-Dassen et al. in the context of dementia (26), describing two dimensions: the individual and the social environment (**Table 1**). The individual level includes the capacity to fulfill one’s potential (competencies) and obligations (social demands); the ability to manage life with some degree of independence (despite cognitive decline and/or dementia); and the ability to participate in social activities. The environmental level involves the domains: structure/infrastructure (e.g., size, density, or types of relationships); functions served by the immediate network (e.g., emotional support, instrumental aid; appraisal of the quality of the relationship and interaction (e.g., relationship quality and satisfaction).

**Table 1:** Social Health framework used to systematize instruments (26).

	Level	Domains	Definition	Examples of markers
Social Health A relational concept in which well-being is defined by how individuals relate to their social environment and how the social environment relates to individuals	A. Individual	A1. Capacities	The capacity to fulfill one’s potential (competencies) and obligations (social demands)	exercising choice, to comply or not with social norms/expectations
		A2. Independence	The ability to manage life with some degree of independence (despite a medical condition)	autonomy, having control, or freedom in following own norms, participating in shared decision-making
		A3. Social participation	The ability to participate in social activities	playing together, visiting friends, joining social events
	B. Social environment^a^	B1. Structure	Social (infra)structure allows interaction and also potentially access to resources and material goods	size, density or types of relationships, family or friends’ networks or network diversity, frequency of social contacts, social isolation, marital status
		B2. Functions	Functions served by the immediate network	emotional (expressions of empathy, love, trust, and caring) or informational support (advice, suggestions, and information)
		B3. Appraisal	Appraisal of the quality of the relationship and interaction	satisfaction with social ties, loneliness, or stigma experience

a Immediate circle around the individual.

Now that a conceptual framework for social health with six clearly defined domains has been proposed, the next step is to operationalize its use in practice-based research, as was suggested by Moniz-Cook et al. (27). This requires sound domain-specific instruments for the measurement of social health in dementia to evaluate new emerging psychosocial interventions that support people with dementia to live well with the condition.

The present study seeks to address the gap in our knowledge of assessment of social health in relation to cognition and dementia in older people. The added value is the use of a clear conceptual framework to examine the psychometric qualities of relevant instruments used in older adult populations.

This review addresses the following four objectives:

1. To identify instruments used to measure social health markers within the context of cognitive functioning, cognitive decline, and dementia;
2. To provide an overview of the characteristics, context, and psychometric properties of these instruments;
3. To examine the consistency of how social health markers are measured; and

To identify social health domains that lack coverage with validated measurement tools and make recommendations for the future development of social health measures.4.

## 2 Materials and methods

### 2.1 Search strategy

This systematic review was conducted following the PRISMA 2020 guidelines (28) and registered at the Open Science Framework (OSF) platform (DOI: 10.17605/OSF.IO/8J9YT) (see **Supplementary Table S1** for the PRISMA 2020 checklist). Three electronic databases were independently searched by four reviewers (H.W., M.L.B., I.S., T.R.): PubMed/MEDLINE, PsychINFO, and CINAHL for publication records in English. These databases were selected due to their comprehensive coverage of relevant literature in the fields of medicine, psychology, and nursing sciences. These databases offer a high number of peer-reviewed articles and are at the forefront of providing research findings on dementia and social health. The online search covered the publication period from January 2000 till July 2020. An updated search covering the period from August 2020 to September 2023 was conducted independently by two additional reviewers (J.A. and J.M.).The search strategy included terms and synonyms for 1) dementia (i.e., Alzheimer’s, cognitive impairment); 2) social health markers (i.e., decision-making, social participation, loneliness), and 3) instruments (i.e., tools, measures). An example of the full search string for PsycINFO can be found in **Supplementary Table S2**. In addition, reference lists from eligible publications were checked to identify all potentially relevant articles. The search results were uploaded to a reference management software (Citavi 6) to organize and store the data and remove duplicates. Then the results were downloaded into the Rayyan application – a free web tool for the organization and documentation of the screening process (29). First, after removing duplicates, four reviewers (H.W., M.L.B., I.S., T.R.) independently screened titles and abstracts, then full texts against the inclusion and exclusion criteria. For the search update (publication period 2020-2023) two reviewers (J.A. and J.M.) screened the additional studies. A consensus approach through discussion was taken to resolve discrepancies between reviewers. At this stage, a group of experts provided the review team with five literature reviews published in recent years on instruments measuring social or psychosocial aspects within relevant epidemiological and/or dementia research (10, 22–24, 27). These publications were suggested for comparison with the results from the database search. The search strategy involved comprehensive and well-structured searches across key databases, using relevant terms to identify studies on social health in dementia, which were then carefully screened.

### 2.2 Eligibility criteria in the main literature review

Inclusion criteria were structured around outcomes, study design, study setting, and population of interest. The publications were screened based on the following criteria: (1) the study focuses either on a population with dementia/Alzheimer’s or on cognitive decline/impairment in older people; (2) longitudinal, case-control, and cross-sectional cohort studies; (3) the studies used validated, multi-item instruments measuring at least one social health marker; and (4) any primary research papers, peer-reviewed publications, published in English. The following exclusion criteria were applied: (1) the population under investigation had a clearly defined other primary diagnosis (i.e.) nondementia/cognitive decline); (2) the study focuses on formal/informal caregivers/physicians; (3) nonprimary research papers (e.g., reviews, commentaries, protocols, unpublished dissertations, conference proceedings, conference abstracts); and (4) pharmacological studies and animal model studies. An exception to the exclusion criteria regarding “reviews” were the five reviews (10, 22–24, 27) received from the experts since it was important to capture any missing instruments from the database search. In summary, these criteria ensure that only studies directly measuring social health in people with dementia or cognitive decline using reliable tools are included while excluding studies on unrelated populations or nonempirical research.

### 2.3 Data extraction and synthesis

The data of the included articles were extracted by two researchers independently (H.W., M.L.B.). The extraction chart included characteristics of the instruments: (1) instrument’s name; (2) authors; (3) year of first publication; (4) way of data collection; (5) target population; (6) target setting; (7) number of items; (8) subscales; (9) scoring; (10) reliability; (11) validity; (12) responsiveness to change; (13) applied for dementia population: yes/no; (14) revisions/adaptations; (15) references: authors and year; and (16) names of subscales or items categorized to the six social health domains. Results were described and summarized narratively, according to three steps: (1) tabulation of the results into a predefined categorical framework; (2) the analysis of extracted data; and (3) a synthesis of the findings under each domain (30).

### 2.4 Quality and risk of bias assessment

As this review focuses on the identification and assessment of existing social health instruments, we did not evaluate the studies’ quality and/or risk of bias. The decision not to systematically assess the quality of the individual studies was made deliberately to focus specifically on the comprehensive identification and categorization of instruments already being used. However, we checked the original validation and the psychometric characteristics of the identified instruments.

## 3 Results

### 3.1 Identification of social health instruments

**Figure 1:**
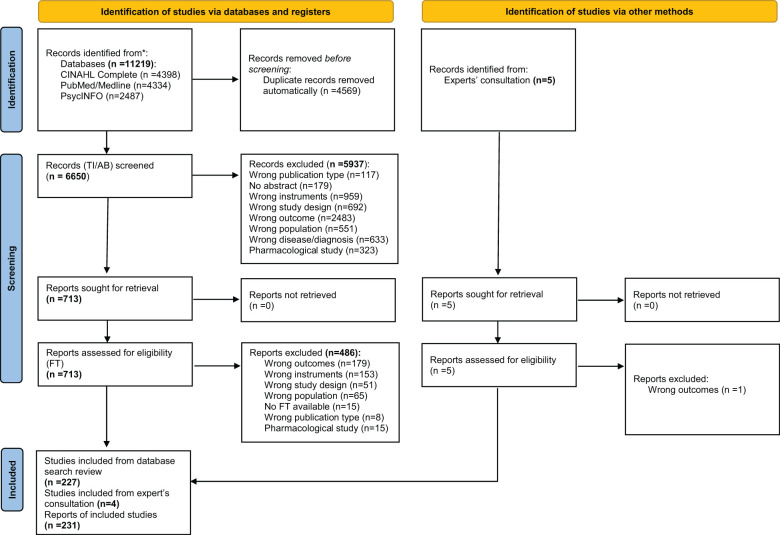
PRISMA flow diagram (title/abstract (TI/AB), full text (FT)).

The online searches identified 11,219 publication records (**Figure 1**). After removing duplicates (n = 4,541), 6,678 titles and abstracts were double-screened, resulting in 741 publications selected for full-text review. Of these, 146 studies met the eligibility criteria. Additionally, five publication records received from the experts were included in the main sample for the review to capture any missing instruments from the database search. One study (27) was excluded as none of the mentioned instruments were relevant to our study. Identified instruments (n = 92) from the literature databases were compared with the instruments found in the four additional papers. Based on this procedure, we included a further 10 new instruments. The additional identification by expert opinion shows that our approach to literature search is flexible and open to additions to ensure the most complete coverage possible. Thus, a total of 227 studies, with 102 instruments are included in this review and synthesis (see **Supplementary Table S3** for references of all studies included).

### 3.2 Overview of the characteristics of studies

The general information on the characteristics of the 102 instruments included in the synthesis is shown in **Supplementary Table 4**.

Social Health Domain(s): (A1) The capacity to fulfill one’s potential (competencies) and obligations (social demands); (A2) The ability to manage life with some degree of independence (despite a medical condition); (A3) The ability to participate in social activities; (B1) Structure/infrastructure (e.g., size, density, or types of relationships); (B2) Functions served by an immediate network (e.g., emotional support, instrumental aid); (B3) Appraisal of the quality of the relationship and interaction (e.g., relationship quality and satisfaction).

The majority of studies were conducted in the USA (n = 77), UK (n = 31), China (n = 18), the Netherlands (n = 14), followed by Australia (n = 12), Germany (n = 10), Japan (n = 10), Taiwan (n = 10), and Italy (n = 8). As we included a broad range of study types (i.e., feasibility studies, cross-national panel studies) sample sizes show a broad range between 7 and 21,241, with a median of 222.

**Figure 2:**
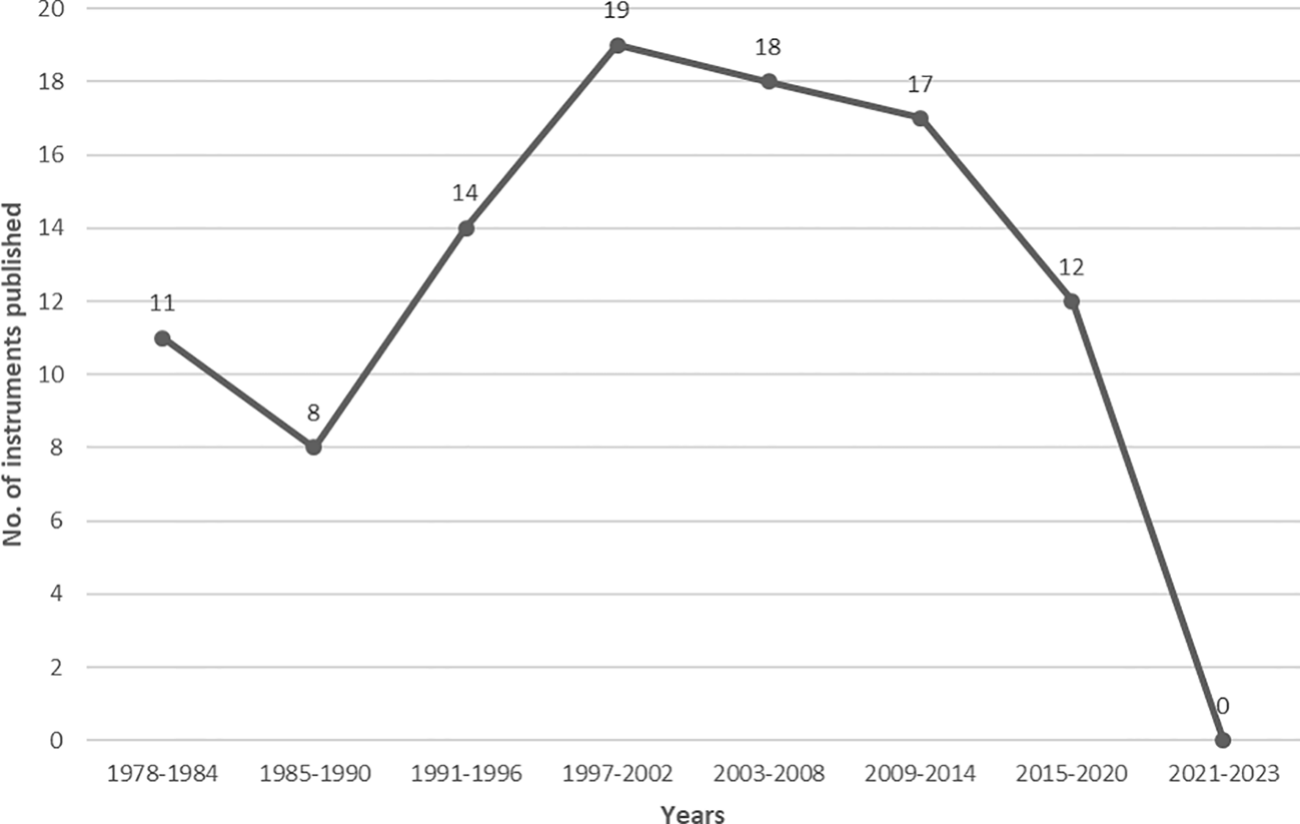
Date of first publication of included instruments.

Date of first instrument publication ranged from 1978 to 2023 (**Figure 2**), with most instruments published between 1997 and 2002 (n = 19), 2003 and 2008 (n = 18), and 2009 and 2014 (n = 17). In the most recent period (2021–2023), there are no publications on new instruments, which might indicate that there is a certain degree of saturation and that already existing instruments are predominantly used.

**Figure 3:**
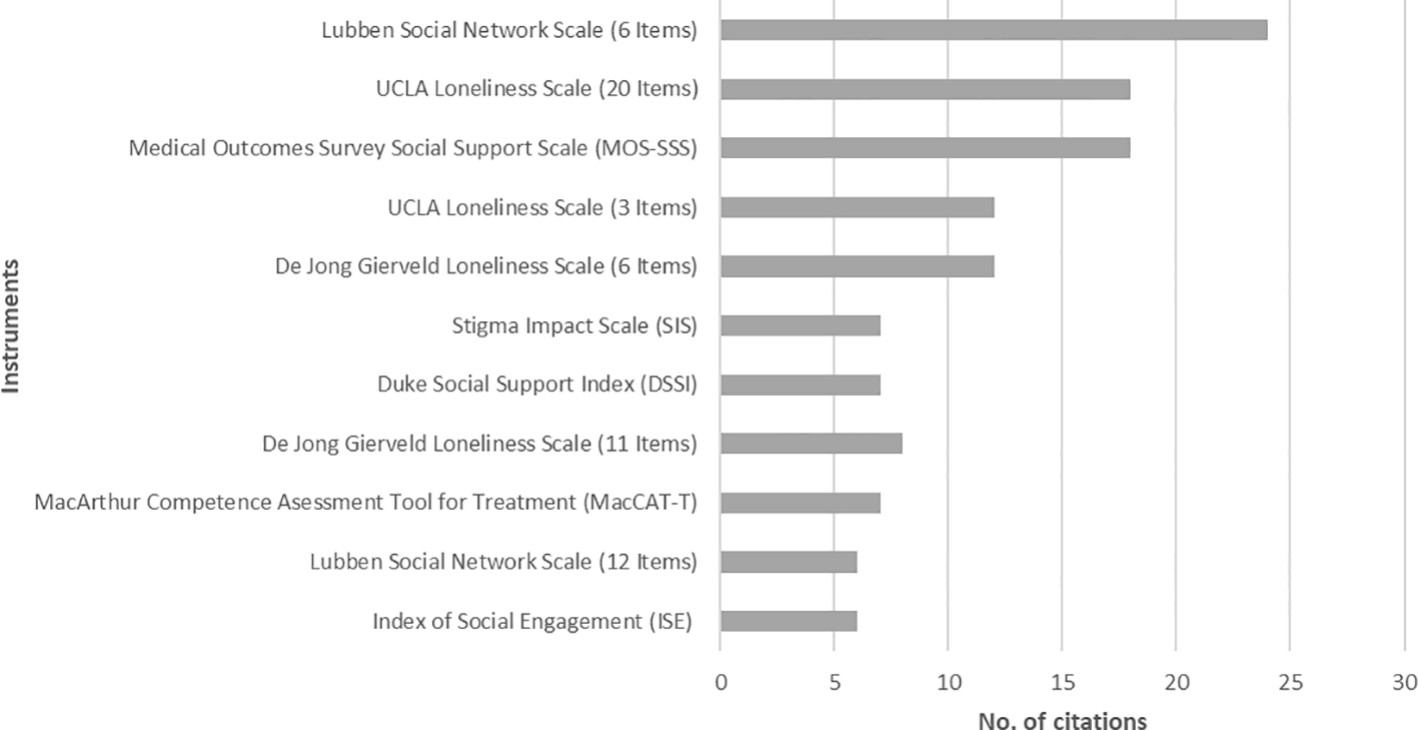
Most frequently cited instruments (*n* = 11).

The three most frequently reported instruments were the Lubben Social Network Scale with 6 items, the Medical Outcomes Survey Social Support Scale (MOS-SS), and the 20-item version of the UCLA Loneliness Scale. **Figure 3** shows all instruments that were cited in at least five of the studies included.

### 3.3 Overview of the characteristics of identified instruments

Our sample of instruments consists predominantly of self-report measures in which respondents are asked to report directly on their social health (*n* = 82; 80.4%). Thirteen instruments (12.7%) were developed as observation tools to be used by significant others (proxies), like formal or informal caregivers. Furthermore, we found a hybrid instrument type, for which both self-report and observation/proxy versions are available (*n* = 6; 5.8%).

Regarding the length of instruments, measured by the number of items, the sample shows a range from 3 to 126 items to be answered, with a median of 12 items. The shortest scales, all with three items, are the UCLA-3 Loneliness Scale (UCLA-3-LS), the Oslo Social Support Scale (OSSS), the Linguistic Instrument for Medical Decision-Making (LIMD), and the CollaboRATE Scale to determine shared decision-making. The most extensive scale is the Social Observation Behaviors Residents Index (SOBRI) with 126 items, followed by the Social Performance Survey (SPS) with 100 items.

**Figure 4:**
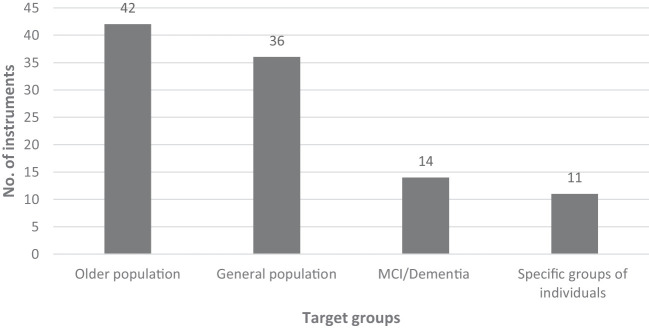
Target groups the social health instruments were designed for.

As studies either on dementia or Alzheimer’s populations or on cognitive decline and cognitive impairment in older adults were eligible to be included in this review, the target populations show a broader spectrum (**Figure 4**). Based on the authors’ reporting, classification into multiple population categories was possible. Most of the included instruments were developed for older populations (with different information on age cut-offs) without further specification (39; 38.2%). The second largest group of instruments consists of tools designed for the general population, free of targeting a certain older age group or a medical condition (34; 33.3%). Even if the tools are designed for the general population, they can be significantly relevant to PlwD or people with cognitive disorders. Especially by collecting data on proxies, such as family carers, these instruments can provide valuable insights into the social health and well-being of those affected. Seventeen instruments (16.7%) are specifically aimed at MCI or dementia populations. Three instruments (LIMD, PES-AD short form, ADOC) focus specifically on predementia MCI, and four tools on mild dementia (CCTI, CAT-V, EID-Q, ISE), three on moderate forms of dementia (CCTI, CAT-V, ISE), and two are designed for populations with severe dementia (CAT-V, ISE). Furthermore, nine instruments do not specify the stage of dementia for which they are designed (DMI, LIMD, PES-AD short form, SF-DEM, SOBRI, SDS, SIS, FS-ADS, ASDS). Twelve instruments target specific groups of individuals, e.g., nursing home residents, mental health patients, brain injury patients, psychiatric inpatients, schizophrenia patients, or palliative care patients.

### 3.4 Psychometrics

Reporting of the psychometric quality of the instruments varies considerably (**Supplementary Table 4**). Only a few studies reported detailed data, and for many studies, additional research was needed. The availability of psychometric information differed. Reliability was accessible almost for all instruments (*n* = 101), and information on validity for *n* = 75 (73.5%). For 69 tools (67.6%) both data on reliability and validity are available. In addition to information on validity and reliability, information on responsiveness to change was only available for four (3.9%) of the included tools (LLFDI, PES-AD, RNLI, SASS). For all these four instruments prospective data indicated the sensitivity to disease progression. In PES-AD dependence levels increased significantly over time. Investigating and reporting responsiveness is essential for advancing our understanding of social health, particularly in the context of dementia. While challenges exist, especially with self-report measures and the need for longitudinal data, the benefits of having responsive tools outweigh these difficulties.

### 3.5 Classification of instruments using the six social health domains

**Figure 5:**
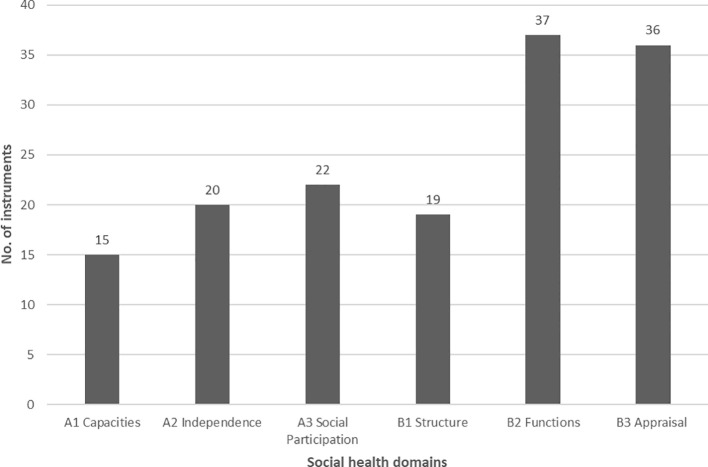
The number of instruments categorized in social health domains (**(A)** individual, **(B)** social environment).

The categorization of instruments into social health domains (**Table 2**) was based on their key thematic concepts. These key thematic concepts are reflected by subscales or groups of items (**Figure 5**; **Supplementary Table 4**). Categorization of a whole instrument into more than one social health domain was allowed. For instance, if a tool has multiple subscales, each representing a different social health domain (i.e., subscale a measures structural aspects, subscale b functional aspects, and subscale c the appraisal of social health).

**Table 2:** Six social health domains attributed to instruments (*n* = 102).

Name of instrument	Name of domains					
	A1. Capacities	A2. Independence	A3. Social participation	B1. Structure	B2. Functions	B3. Appraisal
Engagement and Independence in Dementia Questionnaire (EID-Q)	X	X	X		X	X
Personal Resource Questionnaire (PRQ2000)	X		X	X	X	X
Social role subscale of the Late Life Function and Disability Instrument (LLFDI)	X		X	X		
Patient Dignity Inventory (PDI)	X	X				X
Aid for Decision-Making in Occupation Choice (ADOC)	X		X		X	
Arizona Social Support Interview Survey (ASSIS)				X	X	X
Oslo Social Support Scale (OSSS), Duke Social Support Index (DSSI-23)				X	X	X
the German Version of the Maastricht Electronic Daily Life Observation Tool (MEDLO-tool), social isolation using a 15-item scale			X	X	X	
Social Adaptation Self-Evaluation Scale (SASS)	X	X				
Inventory of Interpersonal Situations (IIS)	X				X	
Social Distance Scale (SDS)	X					X
Ability Assessment of Older Adults		X	X			
Social functioning Scale specific to PD (PDSFS)		X			X	
Apathy Motivation Index (AMI)		X	X			
Social Functioning in Dementia Scale (SF-DEM), Social Interaction Scale (SIS), Social Interaction Scale (SIS)			X	X		
Revised Index for Social Engagement (RISE)			X			X
Collaborative Research on Ageing in Europe Social Network Index (COURAGE-SNI)				X	X	
International Mobility in Aging Study - Social Network Support Scale (IMIAS SNSS), Berkman Syme Network Index (SNI), Social Support Rating Scale (SSRC), Social Capital Scale to cover the two dimensions of social capital (social cohesion and social interaction)				X	X	
Social Disconnectedness and Perceived Isolation Scale (SDPI), IMIAS social network scale				X		X
Interview Schedule for Social Interaction (ISSI), Social Provisions Scale, Social Support Questionnaire Short-Form (SSQ6), ten-item Family Orientation sub-scale of the Chinese Personality Assessment Inventory (CPAI-2), Perceived Social Isolation Scale, 13-item version of the Marlowe-Crowne Social Desirability Scale					X	X
Marlowe-Crowne Social Desirability Scale - Short Form, Social-Adaptive Functioning Evaluation (SAFE), Social Norms Questionnaire (SNQ), Social Performance Survey (SPS), Social Problem-Solving Inventory-Revised: long version (SPSI-R:L), Social Vulnerability Scale (SVS-15), Socioemotional Dysfunction Scale (SDS), Socio-Emotional Questionnaire (SEQ)	X					
CollaboRATE Scale, Capacity to Consent to Treatment Instrument (CCTI), Competence Assessment Tool for Voting (CAT-V), Decisional Conflict Scale (DCS), Engagement and Independence in Dementia Questionnaire (EID-Q), Evaluation to Sign Consent (ESC), Health Care Empowerment Questionnaire (HCEQ), Linguistic Instrument for Medical Decision-Making (LIMD), MacArthur Competence Assessment Tool for Clinical Research (MacCAT-CR), Melbourne Decision Making Questionnaire (MDMQ), Modified Healthcare and Financial Decision-Making Measure, Satisfaction with Decision Scale (SWD)		X				
Social role subscale of the Late Life Function and Disability Instrument (LLFDI), Index of Social Engagement (ISE), Minimum Data Set Social Engagement Measure (SocE), Passivity in Dementia Scale (PDS), Reintegration to Normal Living Index (RNLI), Restorative Activity Questionnaire (RAQ), Pleasant Events Schedule-AD short form (PES-AD), Personal Resource Questionnaire (PRQ2000), Victoria Longitudinal Study Activity Questionnaire (VLS), social engagement (SE) index, Florida Cognitive Activities Scale (FCAS), 12 items from—Healthy Aging Questionnaire, and the Leisure Activities Questionnaire (LAQ)			X			
Personal Resource Questionnaire (PRQ2000), Social role subscale of the Late Life Function and Disability Instrument (LLFDI), Lubben Social Network Scale (LSNS-6), Practitioner Assessment of Network Type (PANT), “name generator” approach				X		
2-Way Social Support Scale (2-Way SSS), Duke/UNC Functional Social Support Questionnaire (DUFSSQ), ENRICHD Social Support Inventory (ESSI), German Social Support Questionnaire—Short version, Interpersonal Support Evaluation List (ISEL-40), Lubben Social Network Scale (LSNS-6), Medical Outcomes Study Social Support Survey (MOS-SSS), Multidimensional Scale of Perceived Social Support (MSPSS), Personal Resource Questionnaire (PRQ2000), Observed Emotion Rating Scale (OERS), IMIAS’s Social Support Scale, Social Network Questionnaire 8SNQ) 27 items, the social support and strain scales Apathy Evaluation Scale-informant version (AES-I)					X	
Engagement and Independence in Dementia Questionnaire (EID-Q), Anticipated Cost of Stigma Scale (ACSS), De Jong Gierveld Loneliness Scale (6-item), Dyadic Trust Scale (DTS), Family Emotional Involvement and Criticism Scale (FEICS), Friendship Scale (FS), Partnership Questionnaire (PQ), Patient Dignity Inventory (PDI), Positive Affect Index (PAI), Scale for Quality of the Current Relationship in Caregiving (SQCRC), Stigma Experience Scales (SES), Stigma Impact Scale (SIS), UCLA-3 Loneliness Scale (UCLA-3-LS), perceived stigma assessment tool, Inventory of Interpersonal Situations (IIS), Family Stigma in Alzheimer’s Disease Scale (FS-ADS), The Positive Affect Index (PAI), dementia-related stigma scale, Japanese version of the assessment scale of dementia stigma (ASDS), Japanese version of the Rosenberg Self-Esteem Scale (RSES-J), 6-item Life Engagement Test						X

The domains represent a mixture of social and psychological health. However, the focus is on social health, but social and psychosocial health are often closely intertwined.

In total, on the individual social health level (level A), we classified 58 instruments, while on the social environment level (level B), 92 instruments. The greatest number of instruments (*n* = 37) was identified in the B2 domain “Functions”, while the least in the domain A1 “Capacities” (*n* = 16). The majority of instruments (*n* = 69) were designed to measure one social health domain, 22 instruments measured two domains, and 10 instruments measured three domains. Two instruments, EID-Q and PRQ2000 cover five out of six social health domains (EID-Q without domain B1 “Structure”, PRQ200 without domain A2 “Independence”). The EID-Q self-report tool includes 26 items across five subscales, possible to be categorized into the following social health domains (with items examples): (A1) social role (“I have a role in my social circle”, Reciprocity subscale), (A2) decision-making (“I can make my own decisions as much as I’d like to”), autonomy (“I can get myself food if I need to”, Activity of daily living subscale), (A3) activity engagement (“I can do activities that are important to me”), (B2) social support (“There are people I could ask for help if I need to”), (B3) reciprocity (“I feel ignored by those around me”, “I feel connected to others”). Answers refer to the feelings over the past month only. The target population of the instrument is PlwD (mild dementia), it is validated and shows good reliability. The EID-Q does not include items that could measure structural aspects of social health (social network size, frequency of interaction, or participation and has yet to demonstrate responsiveness to a relevant psychosocial intervention. Arising from the discipline of positive psychology it nevertheless represents a first step in developing a tool for assessing both the abilities of an individual person with dementia within his or her immediate social environment as well as the influence the social environment may have on the person’s functioning. The PRQ2000 self-report tool includes 15 items and is separated into two parts. The first part is about descriptive information on social networks and the second part comprises the assessment of the perceived level of social support. The tool can be classified into the following social health domains (with items examples): (A1) social support (“I have the opportunity to encourage others to develop their interests and skills”), (A3) social activities (“I spend time with others who have the same interests that I do”), (B1) social security (“There is someone I feel close to who makes me feel secure”), (B2) feeling of belonging (“I belong to a group in which I feel important”), (B3) reciprocity (“Among my group of friends we do favors for each other”. The PRQ2000 does not include items that could measure the degree of independence and also has yet to demonstrate responsiveness to a relevant psychosocial intervention. The instrument can be used for the general population and shows good validity and reliability.

#### 3.5.1 Domain: the capacity to fulfill one’s potential and obligations

Sixteen instruments cover the first domain, “Capacities”, on the individual level (**Table 2**). Based on the categorization, the markers of this social health domain include emotion recognition, empathy, social conformity, antisocial behavior, sociability, extraversion, openness, appropriateness, maladjustment, tendency to unquestioningly believe things that are unlikely to be true (credulity), and susceptibility to exploitation (gullibility), self-perception of one’s ability to manage and control his environment (social adjustment), self-evaluation of social distancing, social problem orientation, styles of social problem-solving, social skills, and communication skills, knowledge of social norms, social rigidity/endorsement of socially appropriate behavior, social competence, cooperativeness, and life skill functioning, social role (provide care to others, voluntary work), dignity-related social distress (not being able to carry out important social roles), the tendency to act socially desirable.

#### 3.5.2 Domain: the ability to manage life with some degree of independence (despite cognitive decline and/or dementia)

Twenty instruments were classified into the second domain, “Independence”. Based on the categorization, the markers of this social health domain include (shared) decision-making (financial, healthcare), consent abilities (understanding, reasoning, expressing choice), capacity to vote, level of comfort with a decision, level of involvement in a variety of daily decisions, empowerment, voluntariness, individuality, self-direction, the ability to evaluate risks and benefits of a medical decision, expressing decision, appreciation of shared decision-making, decision-making style (vigilance, buck-passing, procrastination, hypervigilance), involvement and satisfaction with decision-making, dignity-related social distress (not being able to attend to own bodily functions independently—needing assistance), self-perception of one’s ability to manage and control his environment.

#### 3.5.3 Domain: the ability to participate in social activities

Twenty-three instruments cover the third domain “Social participation”. Based on the categorization, the markers of this social health domain include social engagement, interacting (spending time) with others, sensitivity to other people, self-initiated activities, involvement in the life of a facility, participation in daily social activities, group involvement, family/community interaction, institutional interaction, participation in social-private, social-public activities, inviting people, being in touch with others.

#### 3.5.4 Domain: structure/infrastructure of social network

Twenty instruments cover the first domain, “Structure”, on the social environment level. Based on the categorization, the markers of this social health domain include structural (size) aspects of individuals’ social network, no. of friends, social isolation, frequency of family/community interaction, and frequency of social participation.

#### 3.5.5 Domain: functions served by the immediate social network

Thirty-seven instruments cover the second domain, “Functions”, on the social environment level. Based on the categorization, the markers of this social health domain include social support (instrumental, emotional, tangible, affectionate, appraisal, self-esteem, belonging, informational, and confidant), social integration, reassurance of worth, reliable alliance, guidance, and nurturance, availability of assistance, easiness getting help from neighbors, help from and discussing private matters with family or friends, availability of social interaction, availability of attachment, physical assistance, and feedback.

#### 3.5.6 Domain: appraisal of the quality of the relationship and interaction

Thirty-six instruments cover the third domain, “Appraisal”, on the social environment level. Based on the categorization, the markers of this social health domain include: stigmatization (being treated differently, excluded, criticized, having lower expectations, being judged, distrusted, viewed as weak, social rejection, financial insecurity, internalized shame, social isolation), satisfaction with social support, intimate relationship, negatively perceived social interactions (neglect/rejection by others, unwanted intrusion/advice, failure by others to provide help, unsympathetic/insensitive behavior by others), emotional loneliness, social loneliness, dyadic trust (feelings of interpersonal trust in a relationship), reciprocity, perceived criticism and the intensity of emotional involvement from family members, feelings of loneliness, the importance of actual social contacts, adequacy of social interaction, adequacy of attachment, sense of concern/interest from others, perceived partnership satisfaction (conflict behavior, tenderness, commonality/communication), dignity-related social distress (“Feeling that how I look to others has changed significantly”, not being treated with respect or understanding by others), attachment/intimacy, quality of social interaction, relationship quality (closeness, communication, the similarity of views, shared activities, generally getting along), warmth/affection, conflict/criticism, overall satisfaction with the support received.

## 4 Discussion

In this paper, we systematically searched and synthesized the literature on general instruments that measure social health—classified into six domains according to Vernooij-Dassen et al. (26)—in the context of cognitive function and dementia in older people. The key findings are as follows: (1) Most tools are self-reported. (2) Multidomain measures are lacking. (3) Two comprehensive instruments were identified. (4) There are challenges in psychometric reporting, categorization of social health markers, and tool inconsistencies. (5) Longitudinal responsiveness data were limited.

Most of the identified tools are self-reported, indicating the degree of subjectivity, which is a strength when considering the perspectives of PlwD. This type of data collection corresponds with the individual level of social health (three domains: “Capacities”, “Autonomy”, and “Social participation”), emphasizing the importance of limitations and possibilities for the person with dementia. On the other hand, self-reporting imposes subjectivism in defining the social network (level B: social environment). As with the domain Appraisal of the quality of the relationship and interaction, individual evaluation of the quality is necessary, the Structure and Functions domains might require also an external assessment of the social network to get a more differentiated picture. Based on previous studies emphasizing that subjective measures refer to other health-related outcomes than objective measures (15–17), the most accurate and complex (with all the challenges this implies) are the tools with both available assessment versions—self-report and observation/proxy. These, however, constitute a definite minority in our review.

We classified 102 tools across six domains of the applied social health framework (26). The fact that all domains are well covered validates the framework. Our results highlight the shortage of multidomain measures (33 tools, 32.4%) in comparison to single-domain measures (69 tools, 67.6%). This disproportion shows the still present lack of investigating social health as a multidimensional concept, but rather its division into single components, observed in the available studies, somewhat reminiscent of an analytical and medical approach to the function of an organ rather than the whole interconnected organism. Considering the specific nature of social health, understood in the new concept precisely as the interdependence of the individual and the immediate social environment, a holistic approach with domain-specific measures might be of great importance. In our review, we have identified two instruments that cover almost all social health domains, the Engagement and Independence in Dementia Questionnaire (EID-Q (31)), and the Personal Resource Questionnaire (PRQ2000 (32)).

During data synthesis, we faced several difficulties with the identification, extraction, and classification of the instruments. First was poor reporting of psychometrics, scoring, or the number of items—missing information or inconsistent data when referring to the same tool. One potential reason for this was that there were different versions of the same tool. To gather a complete set of the characteristics, we searched for available revised, short or long versions, and cultural adaptations of identified instruments. We did, however, only examine standardized instruments with accessible validity and/or reliability as per our inclusion criteria.

A second difficulty related to responsiveness is known as sensitivity to change over time. This information was only available for four tools, which may reflect either a lack of related longitudinal or intervention studies in cognitive decline and dementia or a problem with the use of self-report measures in tracking the decline of social health aspects in the context of disease progression. This feature becomes especially important when we consider the role of social health from longitudinal studies starting with prevention and continuing across the entire trajectory—from healthy cognitive functioning to severe dementia.

The third difficulty we encountered is related to the categorization of specific markers into social health domains. For example, reciprocity, recognized as the rule of exchanging activities or feelings for mutual benefit, refers to both—reciprocal individual and reciprocal social networks (e.g., family members, carer); thus, we decided to classify it into two domains: Capacity of the individual level (level A) and Appraisal on the social environment level (level B) of the proposed social health framework. The concept of reciprocity has been widely investigated in the dementia literature, pointing to its similarity with social support (reciprocal in nature) but also emphasizing its dynamic nature across the lifetime, especially for PlwD, when the need for being useful increases within the loss of autonomy (31, 33, 34). Examining instrument items was an important task during the classification.

A fourth issue concerned the names of the subscales given by the authors, which were often quite generalized. For example, “social interaction” required careful reading of the items and double checking to verify whether the measurement concerned the frequency of contacts (B1 Structure), accessibility (B2 Functions), or quality (B3 Appraisal). Another example is dignity-related distress, measured by PDI (35), which contains phrases related to both social role (A1 Capacity), independence (A2 Autonomy), and relationship assessment (B3 Appraisal).

Another important element of selection, data extraction, and categorization was the separation of social and psychological constructs, for instance, self-efficacy, which is often understood as an exponent of positive self-understanding (Positive Sense of Self (36);), or a marker of overall psychological well-being in PlwD (31, 37). After multiple discussions with experts (other co-authors), we decided to draw the line between social and psychological health and exclude instruments that measure this concept. On the other hand, we found other constructs such as empathy, antisocial behavior (SEQ (38)), extraversion, and openness (SDS (39)) eligible for inclusion in the domain Capacity, as they refer to the social competencies and capacity to meet social demands. The tools for measuring the sense of loneliness, due to their social dimension, were also included, thus emphasizing the difference between subjective and objective assessment (recognized by social isolation). Therefore, this *a priori* categorization should be noted as one of the review’s limitations. Another challenge in categorizing social health markers was the recognition of the sense of independence (through the A2 Autonomy domain), often measured in older populations or among people with dementia using (I)ADL tools. However, as pointed out by Stoner et al. (31), these tools simplify independence to physical or cognitive abilities without reflecting the essence of the subjective sense of loss of independence in PlwD. A good response to this gap is the Engagement and Independence in Dementia Questionnaire (EID-Q (31)). EID-Q defines the activities of daily life in the context of defining independence as the ability of the PlwD to preserve autonomy despite a medical condition (13, 26). Therefore, we decided to exclude the (I)ADL tools from our review. In contrast, through viewing the autonomy of a person with cognitive deficits as maintaining the ability to make decisions, determined inclusion of important tools such as the CollaboRATE Scale (measuring shared decision-making (40),) or the CCTI (developed for the mild dementia population and capturing the elements of consent abilities, e.g., decisional capacity (33),). Important to note is that marital status was not listed among the markers of domain B1 Structure as it is usually included as a one-item measurement in the metrics (sociodemographic questions) as the basic element in most of the questionnaires. However, only multi-item tools were included in this study.

Overall, the six social health domains are represented in this study by existing instruments. A variety and distribution of instruments are evident, but there is a lack of multidomain measures compared to single-domain tools. Difficulties included poor reporting of psychometric properties, inconsistent data across different versions of instruments, and the categorization of specific markers into appropriate social health domains. Instruments such as the EID-Q and the PRQ2000 represent the first steps toward comprehensive social health assessment, but they still have limitations.

### 4.1 Strengths and limitations

Our study is the first to systematically categorize standardized tools for assessing social health captured in two dimensions—individual social abilities and social network features. Our inclusion of psychometric properties of instruments will facilitate practical and scientific quality comparisons between studies. However, we demonstrate weakness in reported psychometric data and the need for consistent methodological reporting. Our review offers scope for wide-ranging investigation of studies on dementia, cognitive decline, cognitive functioning, and diverse living and care settings. A particular strength of our work is an examination of the responsiveness of the instruments, recognizing the importance of sensitivity to time when applied in the dynamically changing social lives of people with dementia. Overall, the systematic categorization of tools can help social health researchers choose the most appropriate instrument through information on populations and contexts where the tool has been previously used. In addition, the classification given here can be used to combine different instruments to measure social health as a comprehensive concept.

Despite these advantages, we acknowledge several limitations of our review. The first concerns the methodological limitations of the search strategy—including three databases and the use of specific keywords that may result in the omission of relevant articles. For this reason, we decided to consult a group of experts (from the INTERDEM network) that equipped the review team with five literature reviews published in recent years on instruments measuring social or psychosocial aspects within relevant epidemiological and/or dementia research (10, 22–24, 27). These publications were suggested for comparison with the results from the database search and resulted in the inclusion of ten additional instruments into the data set. We also did not consider the study quality, which comprises a limitation and can be valuable in future studies to enable a more robust analysis of the instruments and their suitability for different populations.

The second limitation may be an insufficient representation of tools from diverse world regions due to the inclusion of only English publications (but a significant amount of studies were conducted in Asia) or the exclusion of unpublished instruments for the study of social health. Likely, the understanding and therefore the measurement of social health are different in other cultures than in European countries. This fact could not be fully taken into account in this review. In this sense, the conceptualization of social health could be adapted or possibly expanded for other cultural groups. However, we tried to address this by reporting on available versions (and cultural adaptations) of the instruments, which might show their global popularity. Moreover, for this review, the age limit for older adults was generally defined as 65 years and older, as this is a common standard in mental health research on dementia. This definition may have excluded measures developed specifically for younger people with early-onset dementia. Future studies could include a broader age range to ensure that tools relevant to younger populations are also considered.

Nevertheless, while the psychometric properties differ across countries, it is crucial to conduct further reliability and validation studies. The third limitation concerns the already mentioned *a priori* categorization of the markers, determining the overall operationalization of social health. However, we concede that other ways of categorizing are certainly possible, but rather take our review as an invitation to a deeper discussion in order to better understand the umbrella concept of social health, its key domains, as well as high-quality measures.

### 4.2 Recommendations for the future use and development of social health measures

The categorization of 102 instruments to the novel for dementia care research shows a relatively even and consistent measurement of the six social health domains, with a slight predominance of the Functions domain. The results do not indicate that any domain has an insufficient number of measurement tools; however, the shortage of multidomain instruments is evident. To sufficiently assess social health in its holistic and relational nature, it is essential that tools incorporate multiple aspects of the individual’s social abilities and social network characteristics (26). Moreover, as previous research highlights various health-related outcomes when using subjective and objective measures, introducing alternative versions (self-report or observation/proxy) could benefit by taking a broader perspective. Additionally, tools containing a version to be assessed by, for example, a dementia caregiver are an adequate response to the self-report limitations present in the later stages of the dementia trajectory. The huge range of existing terminology for different social markers (social contacts, networks, interactions, etc.) underlines the urgent need to harmonize the terminology when applying and creating new tools. Also, further research must conduct a specific adaption and validation of existing tools for the general population for PlwD or people with cognitive impairment. In addition to this, the EID-Q and the PRQ2000 instrument have the potential to be further developed in terms of covering all domains.

**Figure 6:**
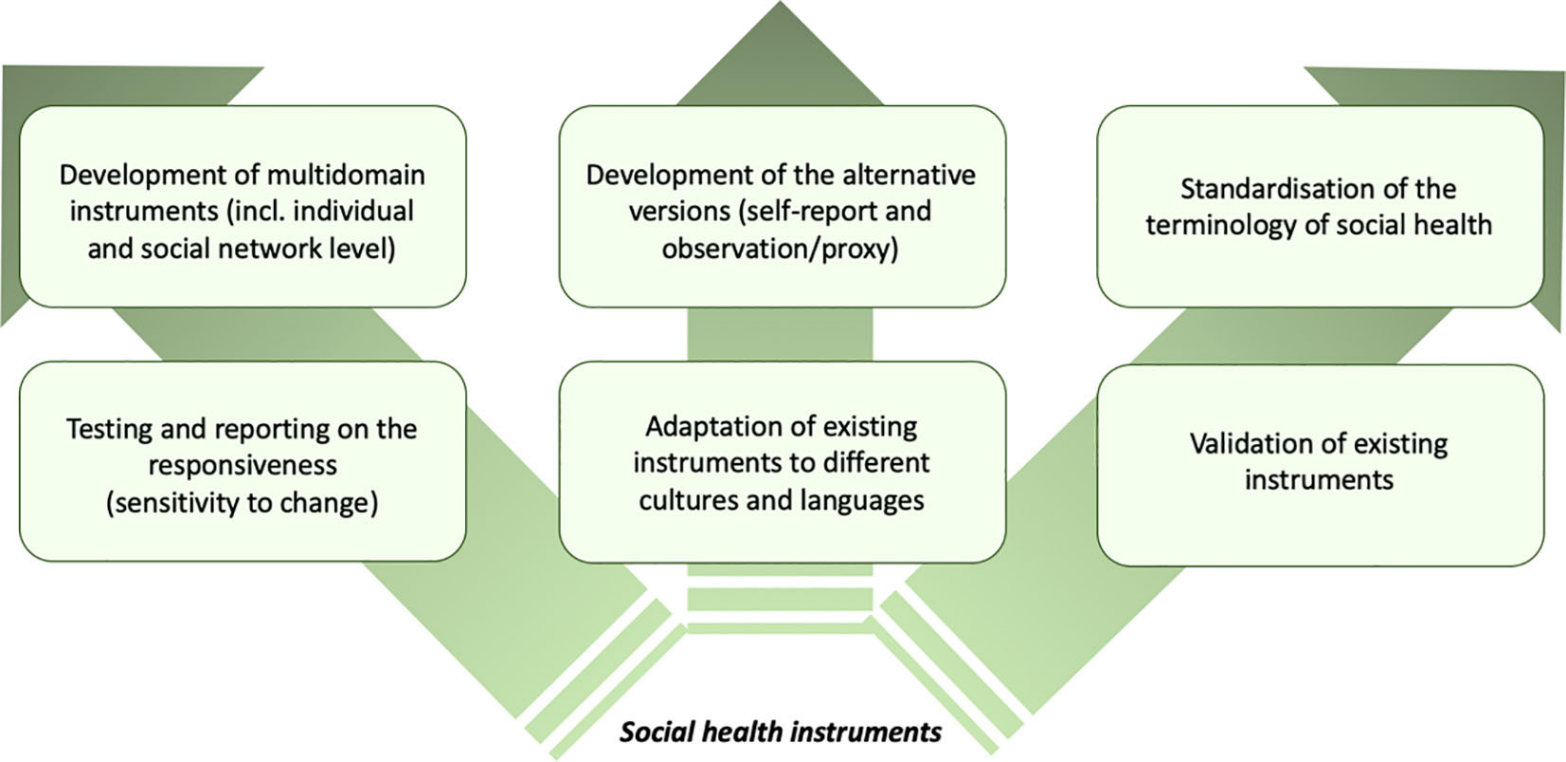
Summary of recommendations for future use and development of social health measures.

However, based on the reported psychometrics of the tools, there is a lack of information about the responsiveness, i.e., the sensitivity to change, which is particularly important when trying to assess social health in longitudinal studies. This, in turn, can help identify tools that are more relevant to a given stage of the dementia trajectory. Valid and reliable instruments that measure social health in individuals with dementia should also be available in culturally different populations by adapting original instruments with promising psychometric properties for different languages. **Figure 6** shows the recommendations for future use and development of social health measures.

## 5 Conclusion

Dementia must be understood as a multidimensional and dynamic phenomenon featuring biological, psychological, and social health aspects (41). Social health is important for the prevention and treatment of dementia, and there is therefore a need for a consistent understanding of the measurement of social health (42).

This study contributes to the discussion about the measurement of social health and shows the potential of developing new instruments to cover the whole social health domain. The analysis emphasizes that only a clear definition of measured areas of social health can help develop appropriate prevention, treatment, and care strategies for PlwD and improve the conditions for living well with dementia. While there are many tools available, there is still a lot of work to be done in the area of their application and the creation of new standardized and terminologically consistent measures of social health and starting a discussion on a core dataset of social health instruments. Moreover, based on the findings of this review, it is recommended that future research and clinical practice adopt a multidimensional approach to assessing the social health of PlwD, highlighting the impact of culture on the measurement of social health in parallel with exploring the use of multidimensional measures. Rather than focusing solely on individual capacities or social network structures, efforts should aim to capture the interplay between these domains as well as the cultural impact to provide a comprehensive understanding.
