# Supplementary material for: Instruments for assessing social health in the context of cognitive decline and dementia: a systematic review
Source: Front Psychiatry. 2024 Nov 13;15:1387192. doi: 10.3389/fpsyt.2024.1387192 (PMC11599264; doi:10.3389/fpsyt.2024.1387192)
Supplement: Supplementary file 2 [file Table2.docx]

Supplementary Table S2: Example of search strategy for Psycinfo via Ovid (Access via University Of Bremen)

| 1. dement*.ti,ab. 2. alzheimer*.ti,ab. 3. MCI.ti,ab. 4. "cognitive impairment".ti,ab. 5. "cognitive decline".ti,ab. 6. 1 or 2 or 3 or 4 or 5 7. ((capacity or reciprocity or "social awareness" or dignity or independence or autonomy or "independent living" or "activities of daily living" or "decision-making" or "self-management" or "social participation" or "social engagement" or "leisure activities" or "meaningful activities" or "social network" or "social environment" or "relationship quality" or "social relationship" or "social isolation" or "community networks" or "social involvement" or "social influence" or stigma* or "social interaction" or "social interaction*" or "social control" or "social support" or "social role*" or "loneliness" or "social contact*" or "social functioning" or "interpersonal relations" or “social health”) adj8 ("survey" or "questionnaire*" or "outcome assessment*" or instrument* or measure* or "measuring*" or inventory or tool or tools or score* or scale* or assessment*)).ti,ab. 8. 6 and 7 9. limit 8 to (abstracts and english language and journal article) 10. limit 9 to yr="2000 - 2020" |
| --- |
