# Supplementary material for: Instruments for assessing social health in the context of cognitive decline and dementia: a systematic review
Source: Front Psychiatry. 2024 Nov 13;15:1387192. doi: 10.3389/fpsyt.2024.1387192 (PMC11599264; doi:10.3389/fpsyt.2024.1387192)
Supplement: Supplementary file 3 [file Table3.docx]

Supplementary Table S3: References of included studies (n=227)

| **No.** | **Study title** | **Reference** |
| --- | --- | --- |
| 1 | Social Support Buffers the Impact of Depressive Symptoms on Life Satisfaction in Old Age | Adams TR, Rabin LA, Da Silva VG, Katz MJ, Fogel J, Lipton RB. Social Support Buffers the Impact of Depressive Symptoms on Life Satisfaction in Old Age. Clin Gerontol. 2016;39(2):139-157. doi: 10.1080/07317115.2015.1073823 |
| 2 | Engagement in purposeful activities and social interactions amongst persons with dementia in special care units compared to traditional nursing homes: An observational study | Adlbrecht L, Nemeth T, Frommlet F, Bartholomeyczik S, Mayer H: Engagement in purposeful activities and social interactions amongst persons with dementia in special care units compared to traditional nursing homes: An observational study. Scand J Caring Sci 2022, 36(3):650-662. |
| 3 | The capacity to vote of persons with Alzheimer’s disease | Appelbaum PS, Bonnie RJ, Karlawish JH. The capacity to vote of persons with Alzheimer's disease. Am J Psychiatry. 2005 Nov;162(11):2094-100. doi: 10.1176/appi.ajp.162.11.2094 |
| 4 | Perceptions of stigma among people affected by early- and late-onset Alzheimer’s disease | Ashworth R. Perceptions of stigma among people affected by early- and late-onset Alzheimer's disease. J Health Psychol. 2020 Mar;25(4):490-510. doi: 10.1177/1359105317720818 |
| 5 | Loneliness and Depression among Community Older Adults during the COVID-19 Pandemic: A cross-sectional study | Alhalaseh L, Kasasbeh F, Al-Bayati M, Haikal L, Obeidat K, Abuleil A, Wilkinson I: Loneliness and Depression among Community Older Adults during the COVID-19 Pandemic: A cross-sectional study. *Psychogeriatrics* 2022, 22(4):493-501. |
| 6 | Perceptions of stigma among people affected by early- and late-onset Alzheimer’s disease | Ashworth R: Perceptions of stigma among people affected by early- and late-onset Alzheimer's disease. J Health Psychol 2020, 25(4):490-510.  Barrenetxea J, Yang Y, Pan A, Feng Q, Koh WP: Social Disconnection and Living Arrangements among Older Adults: The Singapore Chinese Health Study. Gerontology 2022, 68(3):330-338. |
| 7 | Social networks and loneliness in people with Alzheimer's dementia | Balouch S, Rifaat E, Chen HL, Tabet N. Social networks and loneliness in people with Alzheimer's dementia. Int J Geriatr Psychiatry. 2019 May;34(5):666-673. doi: 10.1002/gps.5065 |
| 8 | Supporting autonomy of nursing home residents with dementia in the informed consent process | Beattie E, O'Reilly M, Fetherstonhaugh D, McMaster M, Moyle W, Fielding E. Supporting autonomy of nursing home residents with dementia in the informed consent process. Dementia (London). 2019 Oct-Nov;18(7-8):2821-2835. doi: 10.1177/1471301218761240 |
| 9 | Behavioral and psychological symptoms impact clinical competence in Alzheimer’s disease | Bertrand E, van Duinkerken E, Landeira-Fernandez J, Dourado MCN, Santos RL, Laks J, Mograbi DC. Behavioral and Psychological Symptoms Impact Clinical Competence in Alzheimer's Disease. Front Aging Neurosci. 2017 Jun 16;9:182. doi: 10.3389/fnagi.2017.00182 |
| 10 | Decision-making profile in older adults: the influence of cognitive impairment, premorbid intelligence and depressive symptoms | Biella MM, de Siqueira ASS, Borges MK, Ribeiro ES, Magaldi RM, Busse AL, Apolinario D, Aprahamian I. Decision-making profile in older adults: the influence of cognitive impairment, premorbid intelligence and depressive symptoms. Int Psychogeriatr. 2020 Jun;32(6):697-703. doi: 10.1017/S1041610219001029 |
| 12 | Activities of Daily Living Assessment among Nursing Home Residents with Advanced Dementia: Psychometric Reevaluation of the Bristol Activities of Daily Living Scale | Boyd PA, Wilks SE, Geiger JR. Activities of Daily Living Assessment among Nursing Home Residents with Advanced Dementia: Psychometric Reevaluation of the Bristol Activities of Daily Living Scale. Health Soc Work. 2018 May 1;43(2):101-108. doi: 10.1093/hsw/hly010 |
| 13 | Biopsychosocial Factors and Cognitive Function in Cat Ownership and Attachment in Community-dwelling Older Adults | Branson SM, Boss L, Padhye NS, Gee NR, Trötscher TT (2019) Biopsychosocial Factors and Cognitive Function in Cat Ownership and Attachment in Community-dwelling Older Adults. Anthrozoös. 2019. 32:2, 267-282, DOI: 10.1080/08927936.2019.1569908 |
| 14 | Measuring perceived stigma in persons with progressive neurological disease: Alzheimer’s dementia and Parkinson’s Disease | Burgener SC, Berger B. Measuring perceived stigma in persons with progressive neurological disease: Alzheimer’s dementia and Parkinson’s disease. Dementia. 2008;7(1):31-53. doi:10.1177/1471301207085366 |
| 15 | Perceived stigma in persons with early-stage dementia: Longitudinal findings: Part 1 | Burgener SC, Buckwalter K, Perkhounkova Y, Liu MF, Riley R, Einhorn CJ, Fitzsimmons S, Hahn-Swanson C. Perceived stigma in persons with early-stage dementia: Longitudinal findings: Part 1. Dementia (London). 2015 Sep;14(5):589-608. doi: 10.1177/1471301213508399 |
| 16 | The effects of perceived stigma on quality of life outcomes in persons with early-stage dementia: Longitudinal findings: Part 2 | Burgener SC, Buckwalter K, Perkhounkova Y, Liu MF. The effects of perceived stigma on quality of life outcomes in persons with early-stage dementia: Longitudinal findings: Part 2. Dementia (London). 2015 Sep;14(5):609-32. doi: 10.1177/1471301213504202 |
| 17 | A social model of loneliness: The roles of disability, social resources, and cognitive impairment | Burholt V, Windle G, Morgan DJ; CFAS Wales team. A Social Model of Loneliness: The Roles of Disability, Social Resources, and Cognitive Impairment. Gerontologist. 2017 Nov 10;57(6):1020-1030. doi: 10.1093/geront/gnw125 |
| 18 | Social Disconnection and Living Arrangements among Older Adults: The Singapore Chinese Health Study | Barrenetxea J, Yang Y, Pan A, Feng Q, Koh WP: Social Disconnection and Living Arrangements among Older Adults: The Singapore Chinese Health Study. *Gerontology* 2022, 68(3):330-338. |
| 19 | Adaptation and preliminary psychometric properties of three self-stigma outcome measures for people living with dementia | Bhatt J, Stoner CR, Scior K, Charlesworth G: Adaptation and preliminary psychometric properties of three self-stigma outcome measures for people living with dementia. *BMC Geriatr* 2021, 21(1):34. |
| 20 | Prevalence and correlates of well-being in a cross-sectional survey of older people in Romania attending community day facilities | Caciula I, Boscaiu V, Cooper C. Prevalence and correlates of well-being in a cross-sectional survey of older people in Romania attending community day facilities. The European Journal of Psychiatry. 2019. 33(3). 129-134. doi: 10.1016/j.ejpsy.2019.06.002 |
| 21 | Cognitive stimulation therapy in the Italian context: its efficacy in cognitive and non-cognitive measures in older adults with dementia | Capotosto E, Belacchi C, Gardini S, Faggian S, Piras F, Mantoan V, Salvalaio E, Pradelli S, Borella E. Cognitive stimulation therapy in the Italian context: its efficacy in cognitive and non-cognitive measures in older adults with dementia. Int J Geriatr Psychiatry. 2017 Mar;32(3):331-340. doi: 10.1002/gps.4521 |
| 22 | Qualitative analysis of the capacity to consent to treatment in patients with a chronic neurodegenerative disease: Alzheimer’s disease / Analisi qualitativa sulla capacità a prestare consenso al trattamento in pazienti con malattie cronico degenerative neuropsicoorganiche: Demenza di Alzheimer | Carabellese F, Felthous AR, La Tegola D, Piazzolla G, Distaso S, Logroscino G, Leo A, Ventriglio A, Catanesi R. Qualitative analysis of the capacity to consent to treatment in patients with a chronic neurodegenerative disease: Alzheimer's disease / Analisi qualitativa sulla capacità a prestare consenso al trattamento in pazienti con malattie cronico degenerative neuropsicoorganiche: Demenza di Alzheimer. Int J Soc Psychiatry. 2018 Feb;64(1):26-36. doi: 10.1177/0020764017739642 |
| 23 | Individual differences among older adults with mild and moderate dementia in social and emotional loneliness and their associations with cognitive and psychological functioning | Carbone E, Piras F, Pellegrini FF, Caffarra P, Borella E: Individual differences among older adults with mild and moderate dementia in social and emotional loneliness and their associations with cognitive and psychological functioning. *BMC Geriatr* 2022, 22(1):859 |
| 24 | Predicting functional decline of older men living in veteran homes by minimum data set: implications for disability prevention programs in long term care settings | Chen LY, Liu LK, Liu CL, Peng LN, Lin MH, Chen LK, Lan CF, Chang PL. Predicting functional decline of older men living in veteran homes by minimum data set: implications for disability prevention programs in long term care settings. J Am Med Dir Assoc. 2013 Apr;14(4):309.e9-13. doi: 10.1016/j.jamda.2013.01.017 |
| 25 | Impact of Malnutrition on Physical, Cognitive Function and Mortality among Older Men Living in Veteran Homes by Minimum Data Set: A Prospective Cohort Study in Taiwan | Chen LY, Liu LK, Hwang AC, Lin MH, Peng LN, Chen LK, Lan CF, Chang PL. Impact of Malnutrition on Physical, Cognitive Function and Mortality among Older Men Living in Veteran Homes by Minimum Data Set: A Prospective Cohort Study in Taiwan. J Nutr Health Aging. 2016 Jan;20(1):41-7. doi: 10.1007/s12603-016-0674-5 |
| 26 | The relationship between social interaction and characteristics of aggressive, cognitively impaired nursing home residents | Chen Y-L, Ryden MB, Feldt K, Savik K. The relationship between social interaction and characteristics of aggressive, cognitively impaired nursing home residents. *American Journal of Alzheimer’s Disease*. 2000;15(1):10-17. doi:10.1177/153331750001500108 |
| 27 | Factors influencing the social participation ability of rural older adults in China: A cross-sectional study | Cheng M, Su W, Li H, Li L, Xu M, Zhao X, Han M, Yang L: Factors influencing the social participation ability of rural older adults in China: A cross-sectional study. *Front Public Health* 2022, 10:1001948. |
| 28 | Importance of social relationships in the association between sleep duration and cognitive function: data from community-dwelling older Singaporeans | Cheng GH, Chan A, Lo JC. Importance of social relationships in the association between sleep duration and cognitive function: data from community-dwelling older Singaporeans. Int Psychogeriatr. 2018 Jun;30(6):893-901. doi: 10.1017/S1041610217001041 |
| 29 | Cognitive aids for people with early stage dementia versus treatment as usual (Dementia Early Stage Cognitive Aids New Trial (DESCANT)): study protocol for a randomised controlled trial | Chester H, Clarkson P, Davies L, Hughes J, Islam MS, Kapur N, Orrell M, Peconi J, Pitts R, Poland F, Russell I, Challis D; Members of the HoSt-D (Home Support in Dementia) Programme Management Group. Cognitive aids for people with early stage dementia versus treatment as usual (Dementia Early Stage Cognitive Aids New Trial (DESCANT)): study protocol for a randomised controlled trial. Trials. 2018 Oct 10;19(1):546. doi: 10.1186/s13063-018-2933-8 |
| 30 | The effects of participation in leisure activities on neuropsychiatric symptoms of persons with cognitive impairment: a cross-sectional study | Chiu YC, Huang CY, Kolanowski AM, Huang HL, Shyu YL, Lee SH, Lin CR, Hsu WC. The effects of participation in leisure activities on neuropsychiatric symptoms of persons with cognitive impairment: a cross-sectional study. Int J Nurs Stud. 2013 Oct;50(10):1314-25. doi: 10.1016/j.ijnurstu.2013.01.002 |
| 31 | Can social support and physical activity buffer cognitive impairment in individuals with depressive symptoms? Results from a representative sample of young to older adults | Cohrdes C, Bretschneider J. Can social support and physical activity buffer cognitive impairment in individuals with depressive symptoms? Results from a representative sample of young to older adults. J Affect Disord. 2018 Oct 15;239:102-106. doi: 10.1016/j.jad.2018.07.003 |
| 32 | Altruistic behaviour, but not volunteering, has been associated with cognitive performance in community‐dwelling older persons | Corrêa JC, Ávila MPW, Lucchetti ALG, Lucchetti G. Altruistic behaviour, but not volunteering, has been associated with cognitive performance in community-dwelling older persons. Psychogeriatrics. 2019 Mar;19(2):117-125. doi: 10.1111/psyg.12372 |
| 33 | Influence of hope, social support, and self-esteem in early stage dementia | Cotter VT, Gonzalez EW, Fisher K, Richards KC. Influence of hope, social support, and self-esteem in early stage dementia. Dementia (London). 2018 Feb;17(2):214-224. doi: 10.1177/1471301217741744 |
| 34 | Gray Matter Volume Covariance Networks, Social Support, and Cognition in Older Adults | Cotton K, Verghese J, Blumen HM. Gray Matter Volume Covariance Networks, Social Support, and Cognition in Older Adults. J Gerontol B Psychol Sci Soc Sci. 2020 Jun 2;75(6):1219-1229. doi: 10.1093/geronb/gbz023 |
| 35 | Social network, cognitive function, and dementia incidence among elderly women | Crooks VC, Lubben J, Petitti DB, Little D, Chiu V. Social network, cognitive function, and dementia incidence among elderly women. Am J Public Health. 2008 Jul;98(7):1221-7. doi: 10.2105/AJPH.2007.115923 |
| 36 | Validation of the multidimensional scale of perceived social support (MSPSS) for family caregivers of people with dementia | Cartwright AV, Pione RD, Stoner CR, Spector A: Validation of the multidimensional scale of perceived social support (MSPSS) for family caregivers of people with dementia. *Aging Ment Health* 2022, 26(2):286-293. |
| 37 | Gray Matter Volume Covariance Networks, Social Support and Cognition in Older Adults | Cotton K, Verghese J, Blumen HM: Gray Matter Volume Covariance Networks, Social Support, and Cognition in Older Adults. *J Gerontol B Psychol Sci Soc Sci* 2020, 75(6):1219-1229. |
| 38 | The serial mediating effects of social isolation and resilience on the relationship between fear of dementia and insomnia in community-dwelling older adults | Dai H, Qin J, Huang R, Sun D, Zhang Q: The serial mediating effects of social isolation and resilience on the relationship between fear of dementia and insomnia in community-dwelling older adults. J Adv Nurs 2023, 79(5):1994-2003. |
| 39 | Quality of care and quality of life of people with dementia living at green care farms: a cross-sectional study | de Boer B, Hamers JPH, Zwakhalen SMG, Tan FES, Verbeek H. Quality of care and quality of life of people with dementia living at green care farms: a cross-sectional study. BMC Geriatr. 2017 Jul 19;17(1):155. doi: 10.1186/s12877-017-0550-0 |
| 40 | Regional tau pathology and loneliness in cognitively normal older adults | d'Oleire Uquillas F, Jacobs HIL, Biddle KD, Properzi M, Hanseeuw B, Schultz AP, Rentz DM, Johnson KA, Sperling RA, Donovan NJ. Regional tau pathology and loneliness in cognitively normal older adults. Transl Psychiatry. 2018 Dec 18;8(1):282. doi: 10.1038/s41398-018-0345-x |
| 41 | MARIO Project: Validation and Evidence of Service Robots for Older People with Dementia | D'Onofrio G, Sancarlo D, Raciti M, Burke M, Teare A, Kovacic T, Cortis K, Murphy K, Barrett E, Whelan S, Dolan A, Russo A, Ricciardi F, Pegman G, Presutti V, Messervey T, Cavallo F, Giuliani F, Bleaden A, Casey D, Greco A. MARIO Project: Validation and Evidence of Service Robots for Older People with Dementia. J Alzheimers Dis. 2019;68(4):1587-1601. doi: 10.3233/JAD-181165 |
| 42 | Evaluation of a Digital Companion for Older Adults with Mild Cognitive Impairment | Demiris G, Thompson HJ, Lazar A, Lin SY. Evaluation of a Digital Companion for Older Adults with Mild Cognitive Impairment. AMIA Annu Symp Proc. 2017 Feb 10;2016:496-503. PMID: 28269845 |
| 43 | Association of Higher Cortical Amyloid Burden With Loneliness in Cognitively Normal Older Adults | Donovan NJ, Okereke OI, Vannini P, Amariglio RE, Rentz DM, Marshall GA, Johnson KA, Sperling RA. Association of Higher Cortical Amyloid Burden With Loneliness in Cognitively Normal Older Adults. JAMA Psychiatry. 2016 Dec 1;73(12):1230-1237. doi: 10.1001/jamapsychiatry.2016.2657 |
| 44 | Association of lower spiritual well-being, social support, self-esteem, subjective well-being, optimism and hope scores with mild cognitive impairment and mild dementia | Dos Santos SB, Rocha GP, Fernandez LL, de Padua AC, Reppold CT. Association of Lower Spiritual Well-Being, Social Support, Self-Esteem, Subjective Well-Being, Optimism and Hope Scores With Mild Cognitive Impairment and Mild Dementia. Front Psychol. 2018 Apr 3;9:371. doi: 10.3389/fpsyg.2018.00371 |
| 45 | Empty-nest-related psychological distress is associated with progression of brain white matter lesions and cognitive impairment in the elderly | Duan D, Dong Y, Zhang H, Zhao Y, Diao Y, Cui Y, Wang J, Chai Q, Liu Z. Empty-nest-related psychological distress is associated with progression of brain white matter lesions and cognitive impairment in the elderly. Sci Rep. 2017 Mar 3;7:43816. doi: 10.1038/srep43816. |
| 46 | Social networks in mild-to-moderate Alzheimer disease: longitudinal relationships with dementia severity, cognitive function, and adverse events | Dyer AH, Murphy C, Lawlor B, Kennelly SP, Study Group FTN: Social networks in mild-to-moderate Alzheimer disease: longitudinal relationships with dementia severity, cognitive function, and adverse events. *Aging Ment Health* 2021, 25(10):1923-1929 |
| 47 | Influence of social support on cognitive change and mortality in old age: results from the prospective multicentre cohort study AgeCoDe | Eisele M, Zimmermann T, Köhler M, Wiese B, Heser K, Tebarth F, Weeg D, Olbrich J, Pentzek M, Fuchs A, Weyerer S, Werle J, Leicht H, König HH, Luppa M, Riedel-Heller S, Maier W, Scherer M; AgeCoDe Study Group. Influence of social support on cognitive change and mortality in old age: results from the prospective multicentre cohort study AgeCoDe. BMC Geriatr. 2012 Mar 20;12:9. doi: 10.1186/1471-2318-12-9 |
| 48 | Hallucinations, loneliness, and social isolation in Alzheimer’s disease | El Haj M, Jardri R, Larøi F, Antoine P. Hallucinations, loneliness, and social isolation in Alzheimer's disease. Cogn Neuropsychiatry. 2016;21(1):1-13. doi: 10.1080/13546805.2015.1121139 |
| 49 | Does loneliness mediate the relation between social support and cognitive functioning in later life? | Ellwardt L, Aartsen M, Deeg D, Steverink N. Does loneliness mediate the relation between social support and cognitive functioning in later life? Soc Sci Med. 2013 Dec;98:116-24. doi: 10.1016/j.socscimed.2013.09.002 |
| 50 | Social Cognition and Social Functioning in MCI and Dementia in an Epidemiological Sample | Eramudugolla R, Huynh K, Zhou S, Amos JG, Anstey KJ: Social Cognition and Social Functioning in MCI and Dementia in an Epidemiological Sample. J Int Neuropsychol Soc 2022, 28(7):661-672. |
| 51 | Profiles in emotional aging: does age matter? | Etxeberria I, Etxebarria I, Urdaneta E. Profiles in emotional aging: does age matter? Aging Ment Health. 2018 Oct;22(10):1304-1312. doi: 10.1080/13607863.2017.1286450 |
| 52 | Depressive symptoms in later life: Differential impact of social support and motivational processes on depression in individuals with and without cognitive impairment | Fankhauser S, Drobetz R, Mortby M, Maercker A, Forstmeier S. Depressive symptoms in later life: differential impact of social support and motivational processes on depression in individuals with and without cognitive impairment. Eur J Ageing. 2014 Mar 15;11(4):321-332. doi: 10.1007/s10433-014-0311-2 |
| 53 | Psychosocial Predictors of Cognitive Impairment in the Elderly: A Cross-Sectional Study | Faramarzi M, Zarin Kamar M, Kheirkhah F, Karkhah A, Bijani A, Hosseini SR. Psychosocial Predictors of Cognitive Impairment in the Elderly: A Cross-Sectional Study. Iran J Psychiatry. 2018 Jul;13(3):207-214. PMID: 30319704 |
| 54 | Subjective word-finding difficulty reduces engagement in social leisure activities in Alzheimer’s disease | Farrell MT, Zahodne LB, Stern Y, Dorrejo J, Yeung P, Cosentino S. Subjective word-finding difficulty reduces engagement in social leisure activities in Alzheimer's disease. J Am Geriatr Soc. 2014 Jun;62(6):1056-63. doi: 10.1111/jgs.12850 |
| 55 | Greater Social Engagement and Greater Gray Matter Microstructural Integrity in Brain Regions Relevant to Dementia | Felix C, Rosano C, Zhu X, Flatt JD, Rosso AL: Greater Social Engagement and Greater Gray Matter Microstructural Integrity in Brain Regions Relevant to Dementia. *J Gerontol B Psychol Sci Soc Sci* 2021, 76(6):1027-1035. |
| 56 | Loneliness and depression among elderly nursing home patients | Fessman N, Lester D. Loneliness and depression among elderly nursing home patients. Int J Aging Hum Dev. 2000;51(2):137-41. doi: 10.2190/5VY9-N1VT-VBFX-50RG |
| 57 | Cognition and Perceived Social Support Among Live-Alone Urban Elders | Ficker LJ, MacNeill SE, Bank AL, Lichtenberg PA (2002). Cognition and Perceived Social Support Among Live-Alone Urban Elders. Journal of Applied Gerontology, 21(4), 437–451. doi: 10.1177/073346402237631 |
| 58 | The Impact of Robotic Companion Pets on Depression and Loneliness for Older Adults with Dementia During the COVID-19 Pandemic | Fogelson DM, Rutledge C, Zimbro KS: The Impact of Robotic Companion Pets on Depression and Loneliness for Older Adults with Dementia During the COVID-19 Pandemic. *J Holist Nurs* 2022, 40(4):397-409. |
| 59 | Social networks moderate the association between physical fitness and cognitive function among community-dwelling older adults: a population-based study | Foong HF, Ibrahim R, Hamid TA, Haron SA: Social networks moderate the association between physical fitness and cognitive function among community-dwelling older adults: a population-based study. BMC Geriatr 2021, 21(1):679. |
| 60 | Information processing speed as a mediator between psychosocial stress and global cognition in older adults | Foong HF, Hamid TA, Ibrahim R, Haron SA. Information processing speed as a mediator between psychosocial stress and global cognition in older adults. Psychogeriatrics. 2018 Jan;18(1):21-29. doi: 10.1111/psyg.12279 |
| 61 | Social isolation, social support and loneliness as predictors of cardiovascular disease incidence and mortality | Freak-Poli R, Ryan J, Neumann JT, Tonkin A, Reid CM, Woods RL, Nelson M, Stocks N, Berk M, McNeil JJ et al: Social isolation, social support and loneliness as predictors of cardiovascular disease incidence and mortality. BMC Geriatr 2021, 21(1):711. |
| 62 | Loneliness interacts with family relationship in relation to cognitive function in Chinese older adults | Fung AWT, Lee ATC, Cheng ST, Lam LCW. Loneliness interacts with family relationship in relation to cognitive function in Chinese older adults. Int Psychogeriatr. 2019 Apr;31(4):467-475. doi: 10.1017/S1041610218001333 |
| 63 | Modulating factors that preserve cognitive function in healthy ageing | Fung AW, Leung GT, Lam LC. Modulating factors that preserve cognitive function in healthy ageing. East Asian Arch Psychiatry. 2011 Dec;21(4):152-6. PMID: 22215789 |
| 64 | Quality of life, social support and cognitive impairment in heart failure patients without diagnosed dementia | Gallagher R, Sullivan A, Burke R, Hales S, Sharpe P, Tofler G. Quality of life, social support and cognitive impairment in heart failure patients without diagnosed dementia. Int J Nurs Pract. 2016 Apr;22(2):179-88. doi: 10.1111/ijn.12402 |
| 65 | Perceived stigma towards Alzheimer’s disease and related dementia among Chinese older adults: do social networks matter? | Gao X, Sun F, Prieto L, Iyengar V: Perceived stigma towards Alzheimer's disease and related dementia among Chinese older adults: do social networks matter? *Ageing and Society* 2020, 42(5):1100-1116. |
| 66 | Study of Mental Activity and Regular Training (SMART) in at risk individuals: a randomised double blind, sham controlled, longitudinal trial | Gates NJ, Valenzuela M, Sachdev PS, Singh NA, Baune BT, Brodaty H, Suo C, Jain N, Wilson GC, Wang Y, Baker MK, Williamson D, Foroughi N, Fiatarone Singh MA. Study of Mental Activity and Regular Training (SMART) in at risk individuals: a randomised double blind, sham controlled, longitudinal trial. BMC Geriatr. 2011 Apr 21;11:19. doi: 10.1186/1471-2318-11-19 |
| 67 | A Revised Index for Social Engagement for long-term care | Gerritsen DL, Steverink N, Frijters DH, Hirdes JP, Ooms ME, Ribbe MW. A revised Index for Social Engagement for long-term care. J Gerontol Nurs. 2008 Apr;34(4):40-8. doi: 10.3928/00989134-20080401-04 |
| 68 | Tailoring and evaluating an intervention to improve shared decision-making among seniors with dementia, their caregivers, and healthcare providers: study protocol for a randomized controlled trial | Giguere AMC, Lawani MA, Fortier-Brochu É, Carmichael PH, Légaré F, Kröger E, Witteman HO, Voyer P, Caron D, Rodríguez C. Tailoring and evaluating an intervention to improve shared decision-making among seniors with dementia, their caregivers, and healthcare providers: study protocol for a randomized controlled trial. Trials. 2018 Jun 25;19(1):332. doi: 10.1186/s13063-018-2697-1 |
| 69 | Predictors of community reintegration and quality of life after hip fracture among community-dwelling older adults | Gilboa Y, Maeir T, Weber A, Maeir A, Rotenberg S. Predictors of community reintegration and quality of life after hip fracture among community-dwelling older adults. Int J Rehabil Res. 2019 Sep;42(3):234-239. doi: 10.1097/MRR.0000000000000355 |
| 70 | Mild cognitive impairment predicts institutionalization among older men: a population-based cohort study | Gnjidic D, Stanaway FF, Cumming R, Waite L, Blyth F, Naganathan V, Handelsman DJ, Le Couteur DG. Mild cognitive impairment predicts institutionalization among older men: a population-based cohort study. PLoS One. 2012;7(9):e46061. doi: 10.1371/journal.pone.0046061 |
| 71 | Isolation, Not Loneliness or Cynical Hostility, Predicts Cognitive Decline in Older Americans | Griffin SC, Mezuk B, Williams AB, Perrin PB, Rybarczyk BD: Isolation, Not Loneliness or Cynical Hostility, Predicts Cognitive Decline in Older Americans. *J Aging Health* 2020, 32(1):52-60 |
| 72 | Isolation, not loneliness or cynical hostility, predicts cognitive decline in older Americans | Griffin SC, Mezuk B, Williams AB, Perrin PB, Rybarczyk BD. Isolation, Not Loneliness or Cynical Hostility, Predicts Cognitive Decline in Older Americans. J Aging Health. 2020 Jan-Feb;32(1):52-60. doi: 10.1177/0898264318800587 |
| 73 | Quality of life in community-dwelling older persons with apathy | Groeneweg-Koolhoven I, de Waal MW, van der Weele GM, Gussekloo J, van der Mast RC. Quality of life in community-dwelling older persons with apathy. Am J Geriatr Psychiatry. 2014 Feb;22(2):186-94. doi: 10.1016/j.jagp.2012.10.024 |
| 74 | Cognitive performance predicts treatment decisional abilities in mild to moderate dementia | Gurrera RJ, Moye J, Karel MJ, Azar AR, Armesto JC. Cognitive performance predicts treatment decisional abilities in mild to moderate dementia. Neurology. 2006 May 9;66(9):1367-72. doi: 10.1212/01.wnl.0000210527.13661.d1 |
| 75 | Social Support and Functional Decline in the Oldest Old | Hajek A, Brettschneider C, Eisele M, Mallon T, Oey A, Wiese B, Weyerer S, Werle J, Fuchs A, Pentzek M *et al*: Social Support and Functional Decline in the Oldest Old. *Gerontology* 2022, 68(2):200-208 |
| 76 | Longitudinal Analysis of Outpatient Physician Visits in the Oldest Old: Results of the AgeQualiDe Prospective Cohort Study | Hajek A, Brettschneider C, van den Bussche H, Kaduszkiewicz H, Oey A, Wiese B, Weyerer S, Werle J, Fuchs A, Pentzek M, Stein J, Luck T, Bickel H, Mösch E, Heser K, Bleckwenn M, Scherer M, Riedel-Heller SG, Maier W, König HH. Longitudinal Analysis of Outpatient Physician Visits in the Oldest Old: Results of the AgeQualiDe Prospective Cohort Study. J Nutr Health Aging. 2018;22(6):689-694. doi: 10.1007/s12603-018-0997-5 |
| 77 | Patient participation in medical and social decisions in Alzheimer’s disease | Hamann J, Bronner K, Margull J, Mendel R, Diehl-Schmid J, Bühner M, Klein R, Schneider A, Kurz A, Perneczky R. Patient participation in medical and social decisions in Alzheimer's disease. J Am Geriatr Soc. 2011 Nov;59(11):2045-52. doi: 10.1111/j.1532-5415.2011.03661.x |
| 78 | Discrepancies between cognition and decision making in older adults | Han SD, Boyle PA, James BD, Yu L, Barnes LL, Bennett DA. Discrepancies between cognition and decision making in older adults. Aging Clin Exp Res. 2016 Feb;28(1):99-108. doi: 10.1007/s40520-015-0375-7 |
| 79 | Loneliness as a risk factor for care home admission in the English Longitudinal Study of Ageing | Hanratty B, Stow D, Collingridge Moore D, Valtorta NK, Matthews F. Loneliness as a risk factor for care home admission in the English Longitudinal Study of Ageing. Age Ageing. 2018 Nov 1;47(6):896-900. doi: 10.1093/ageing/afy095 |
| 80 | Improving decision-making for feeding options in advanced dementia: a randomized, controlled trial | Hanson LC, Carey TS, Caprio AJ, Lee TJ, Ersek M, Garrett J, Jackman A, Gilliam R, Wessell K, Mitchell SL. Improving decision-making for feeding options in advanced dementia: a randomized, controlled trial. J Am Geriatr Soc. 2011 Nov;59(11):2009-16. doi: 10.1111/j.1532-5415.2011.03629.x |
| 81 | Environmental predictors of objectively measured out-of-home time among older adults with cognitive decline | Harada K, Lee S, Lee S, Bae S, Harada K, Shimada H. Environmental predictors of objectively measured out-of-home time among older adults with cognitive decline. Arch Gerontol Geriatr. 2019 May-Jun;82:259-265. doi: 10.1016/j.archger.2019.01.021 |
| 82 | Psychological and Environmental Correlates of Moderate-to-Vigorous Physical Activity and Step Counts Among Older Adults With Cognitive Decline | Harada K, Lee S, Lee S, Bae S, Harada K, Suzuki T, Shimada H. Psychological and Environmental Correlates of Moderate-to-Vigorous Physical Activity and Step Counts Among Older Adults With Cognitive Decline. Percept Mot Skills. 2019 Aug;126(4):639-655. doi: 10.1177/0031512519846026 |
| 83 | The theoretical and empirical basis of a BioPsychoSocial (BPS) risk screener for detection of older people’s health related needs, planning of community programs, and targeted care interventions | Hildon ZJ, Tan CS, Shiraz F, Ng WC, Deng X, Koh GCH, Tan KB, Philp I, Wiggins D, Aw S, Wu T, Vrijhoef HJM. The theoretical and empirical basis of a BioPsychoSocial (BPS) risk screener for detection of older people's health related needs, planning of community programs, and targeted care interventions. BMC Geriatr. 2018 Feb 17;18(1):49. doi: 10.1186/s12877-018-0739-x |
| 84 | Loneliness, sadness, and feelings of social disconnection in older adults during the COVID-19 pandemic | Holaday LW, Oladele CR, Miller SM, Duenas MI, Roy B, Ross JS: Loneliness, sadness, and feelings of social disconnection in older adults during the COVID-19 pandemic. *J Am Geriatr Soc* 2022, 70(2):329-340 |
| 85 | Informant-Reported Cognitive Decline and Activity Engagement across Four Years in a Community Sample | Hosking DE, Jiang D, Sargent-Cox KA, Anstey KJ. Informant-Reported Cognitive Decline and Activity Engagement across Four Years in a Community Sample. Gerontology. 2017;63(5):469-478. doi: 10.1159/000475594 |
| 86 | Social isolation and 9-year dementia risk in community-dwelling Medicare beneficiaries in the United States | Huang AR, Roth DL, Cidav T, Chung SE, Amjad H, Thorpe RJ, Jr., Boyd CM, Cudjoe TKM: Social isolation and 9-year dementia risk in community-dwelling Medicare beneficiaries in the United States. *J Am Geriatr Soc* 2023, 71(3):765-773 |
| 87 | Declining medical decision-making capacity in mild AD: a two-year longitudinal study | Huthwaite JS, Martin RC, Griffith HR, Anderson B, Harrell LE, Marson DC. Declining medical decision-making capacity in mild AD: a two-year longitudinal study. Behav Sci Law. 2006;24(4):453-63. doi: 10.1002/bsl.701 |
| 88 | The relationship between social isolation and anxiety in people with cognitive impairment in the UnitedStates | Hwang Y, Massimo L, Aryal S, Hodgson NA: The relationship between social isolation and anxiety in people with cognitive impairment in the United States. *Int J Geriatr Psychiatry* 2022, 37(2) |
| 89 | People with dementia attending farm‐based day care in Norway ‐ Individual and farm characteristics associated with participants’ quality of life | Ibsen TL, Kirkevold Ø, Patil GG, Eriksen S. People with dementia attending farm-based day care in Norway - Individual and farm characteristics associated with participants' quality of life. Health Soc Care Community. 2020 May;28(3):1038-1048. doi: 10.1111/hsc.12937 |
| 90 | Health risk appraisal in older people 2: the implications for clinicians and commissioners of social isolation risk in older people | Iliffe S, Kharicha K, Harari D, Swift C, Gillmann G, Stuck AE. Health risk appraisal in older people 2: the implications for clinicians and commissioners of social isolation risk in older people. Br J Gen Pract. 2007 Apr;57(537):277-82. PMID: 17394730 |
| 91 | Older people with severe loneliness have an atrophied thalamus,hippocampus,and entorhinal cortex | Imai A, Matsuoka T, Narumoto J: Older people with severe loneliness have an atrophied thalamus, hippocampus, and entorhinal cortex. *Int J Geriatr Psychiatry* 2022, 37(12) |
| 92 | Capacity to Vote in Persons with Dementia and the Elderly | Irastorza LJ, Corujo P, Bañuelos P. Capacity to vote in persons with dementia and the elderly. Int J Alzheimers Dis. 2011;2011:941041. doi: 10.4061/2011/941041 |
| 93 | Health risks posed by social and linguistic isolation in older Korean Americans | Jang Y, Yoon H, Park J, Park NS, Chiriboga DA, Kim MT: Health risks posed by social and linguistic isolation in older Korean Americans. *J Am Geriatr Soc* 2021, 69(11):3258-3266 |
| 94 | Living well with dementia: enhancing dignity and quality of life, using a novel intervention, Dignity Therapy | Johnston B, Lawton S, McCaw C, Law E, Murray J, Gibb J, Pringle J, Munro G, Rodriguez C. Living well with dementia: enhancing dignity and quality of life, using a novel intervention, Dignity Therapy. Int J Older People Nurs. 2016 Jun;11(2):107-20. doi: 10.1111/opn.12103 |
| 95 | Walk, Talk and Listen: a pilot randomised controlled trial targeting functional fitness and loneliness in older adults with hearing loss | Jones CA, Siever J, Knuff K, Van Bergen C, Mick P, Little J, Jones G, Murphy MA, Kurtz D, Miller H. Walk, Talk and Listen: a pilot randomised controlled trial targeting functional fitness and loneliness in older adults with hearing loss. BMJ Open. 2019 Apr 14;9(4):e026169. doi: 10.1136/bmjopen-2018-026169 |
| 96 | Social isolation,social support,and loneliness and their relationship with cognitive health and dementia | Joyce J, Ryan J, Owen A, Hu J, McHugh Power J, Shah R, Woods R, Storey E, Britt C, Freak-Poli R *et al*: Social isolation, social support, and loneliness and their relationship with cognitive health and dementia. *Int J Geriatr Psychiatry* 2021, 37(1) |
| 97 | Weak Social Networks in Late Life Predict Incident Alzheimer’s Disease: The Kuakini Honolulu-Asia Aging Study | Kallianpur KJ, Masaki KH, Chen R, Willcox BJ, Allsopp RC, Davy P, Dodge HH: Weak Social Networks in Late Life Predict Incident Alzheimer's Disease: The Kuakini Honolulu-Asia Aging Study. *J Gerontol A Biol Sci Med Sci* 2023, 78(4):663-672 |
| 98 | Alzheimer’s disease patients’ and caregivers’ capacity, competency, and reasons to enroll in an early-phase Alzheimer’s disease clinical trial | Karlawish JH, Casarett DJ, James BD. Alzheimer's disease patients' and caregivers' capacity, competency, and reasons to enroll in an early-phase Alzheimer's disease clinical trial. J Am Geriatr Soc. 2002 Dec;50(12):2019-24. doi: 10.1046/j.1532-5415.2002.50615.x |
| 99 | Social support and cognition in a community-based cohort: the Atherosclerosis Risk in Communities (ARIC) study | Kats D, Patel MD, Palta P, Meyer ML, Gross AL, Whitsel EA, Knopman D, Alonso A, Mosley TH, Heiss G. Social support and cognition in a community-based cohort: the Atherosclerosis Risk in Communities (ARIC) study. Age Ageing. 2016 Jul;45(4):475-80. doi: 10.1093/ageing/afw060 |
| 100 | Trajectories of socio-emotional functioning in early-stage dementia: implications for the individual with dementia and their family carer | Kelly M, Nelis S, Martyr A, Gamble LD, Clare L: Trajectories of socio-emotional functioning in early-stage dementia: implications for the individual with dementia and their family carer. *Aging Ment Health* 2022, 26(5):1069-1077 |
| 101 | Social cognition and social functioning in patients with amnestic mild cognitive impairment or Alzheimer’s dementia | Kessels RPC, Waanders-Oude Elferink M, van Tilborg I: Social cognition and social functioning in patients with amnestic mild cognitive impairment or Alzheimer's dementia. *J Neuropsychol* 2021, 15(2):186-203 |
| 102 | Health factors as potential mediators of the longitudinal effect of loneliness on general cognitive ability | Kim AJ, Beam CR, Greenberg NE, Burke SL: Health Factors as Potential Mediators of the Longitudinal Effect of Loneliness on General Cognitive Ability. *Am J Geriatr Psychiatry* 2020, 28(12):1272-1283 |
| 103 | Assessing the competence of persons with Alzheimer’s disease in providing informed consent for participation in research | Kim SY, Caine ED, Currier GW, Leibovici A, Ryan JM. Assessing the competence of persons with Alzheimer's disease in providing informed consent for participation in research. Am J Psychiatry. 2001 May;158(5):712-7. doi: 10.1176/appi.ajp.158.5.712 |
| 104 | Hippocampus Mediates the Effect of Emotional Support on Cognitive Function in Older Adults | Kim GE, Han JW, Kim TH, Suh SW, Bae JB, Kim JH, Kim KW. Hippocampus Mediates the Effect of Emotional Support on Cognitive Function in Older Adults. J Gerontol A Biol Sci Med Sci. 2020 Jul 13;75(8):1502-1507. doi: 10.1093/gerona/glz183 |
| 105 | Assessment of apathy in neurological patients using the Apathy Motivation Index caregiver version | Klar VS, Ang YS, Lockwood P, Attaallah B, Dickson S, Drew D, Kienast A, Maio MR, Plant O, Slavkova E *et al*: Assessment of apathy in neurological patients using the Apathy Motivation Index caregiver version. *J Neuropsychol* 2022, 16(1):236-258 |
| 106 | The epidemiology of social isolation and loneliness among older adults during the last years of life | Kotwal AA, Cenzer IS, Waite LJ, Covinsky KE, Perissinotto CM, Boscardin WJ, Hawkley LC, Dale W, Smith AK: The epidemiology of social isolation and loneliness among older adults during the last years of life. *J Am Geriatr Soc* 2021, 69(11):3081-3091 |
| 107 | A longitudinal study of the impact of social network size and loneliness on cognitive performance in depressed older adults | Kuiper JS, Smidt N, Zuidema SU, Comijs HC, Oude Voshaar RC, Zuidersma M: A longitudinal study of the impact of social network size and loneliness on cognitive performance in depressed older adults. *Aging Ment Health* 2020, 24(6):889-897 |
| 108 | Examining public stigma of Alzheimer’s disease and its correlates among Korean Americans | Lee SE, Hong M, Casado BL: Examining public stigma of Alzheimer's disease and its correlates among Korean Americans. *Dementia (London)* 2021, 20(3):952-966. |
| 109 | Cognitive Impairment and the Trajectory of Loneliness in Older Adulthood: Evidence from the Health and Retirement Study | Lee JH, Luchetti M, Aschwanden D, Sesker AA, Strickhouser JE, Terracciano A, Sutin AR: Cognitive Impairment and the Trajectory of Loneliness in Older Adulthood: Evidence from the Health and Retirement Study. *J Aging Health* 2022, 34(1):3-13 |
| 110 | Validation of the Chinese translation of the 6-item De Jong Gierveld Loneliness Scale in elderly Chinese | Leung GT, de Jong Gierveld J, Lam LC. Validation of the Chinese translation of the 6-item De Jong Gierveld Loneliness Scale in elderly Chinese. Int Psychogeriatr. 2008 Dec;20(6):1262-72. doi: 10.1017/S1041610208007552 |
| 111 | Social support and family functioning on psychological symptoms in elderly Chinese | Leung KK, Chen CY, Lue BH, Hsu ST. Social support and family functioning on psychological symptoms in elderly Chinese. Arch Gerontol Geriatr. 2007 Mar-Apr;44(2):203-13. doi: 10.1016/j.archger.2006.05.001 |
| 112 | Minimum clinically important difference of the Social Functioning in Dementia Scale (SF-DEM): cross-sectional study and Delphi survey | Levene T, Livingston G, Banerjee S, Sommerlad A: Minimum clinically important difference of the Social Functioning in Dementia Scale (SF-DEM): cross-sectional study and Delphi survey. *BMJ Open* 2022, 12(3):e058252 |
| 113 | Association of social support with cognition among older adults in China: A cross-sectional study | Li B, Guo Y, Deng Y, Zhao S, Li C, Yang J, Li Q, Yan Y, Li F, Li X *et al*: Association of social support with cognition among older adults in China: A cross-sectional study. *Front Public Health* 2022, 10:947225 |
| 114 | Eight-Year Trajectories of Late-Life Loneliness and Incident Dementia: A Nationally Representative Cohort Study | Li Y, Wang X, Guo L, Zhu L, Shi J, Wang W, Lu C: Eight-Year Trajectories of Late-Life Loneliness and Incident Dementia: A Nationally Representative Cohort Study. *Am J Geriatr Psychiatry* 2023, 31(7):475-486 |
| 115 | Relationship of Cognitive and Social Engagement to Health and Psychological Outcomes in Community-Dwelling Older Adults | Liebzeit D, Kuo WC, Carlson B, Mueller K, Koscik RL, Smith M, Johnson S, Bratzke L: Relationship of Cognitive and Social Engagement to Health and Psychological Outcomes in Community-Dwelling Older Adults. *Nurs Res* 2022, 71(4):295-302 |
| 116 | Do people with dementia and mild cognitive impairments experience stigma? A cross-cultural investigation between Italy, Poland and the UK | Lion KM, Szcześniak D, Bulińska K, Evans SB, Evans SC, Saibene FL, d'Arma A, Farina E, Brooker DJ, Chattat R, Meiland FJM, Dröes RM, Rymaszewska J. Do people with dementia and mild cognitive impairments experience stigma? A cross-cultural investigation between Italy, Poland and the UK. Aging Ment Health. 2020 Jun;24(6):947-955. doi: 10.1080/13607863.2019.1577799 |
| 117 | Loneliness is associated with risk of cognitive impairment in the Survey of Health, Ageing and Retirement in Europe | Luchetti M, Terracciano A, Aschwanden D, Lee JH, Stephan Y, Sutin AR. Loneliness is associated with risk of cognitive impairment in the Survey of Health, Ageing and Retirement in Europe. Int J Geriatr Psychiatry. 2020 Jul;35(7):794-801. doi: 10.1002/gps.5304 |
| 118 | Inequality in Social Support Associated With Mild Cognitive Impairment: A Cross-Sectional Study of Older (≥60 Years) Residents in Shanghai, China | Lu Y, Liu C, Fawkes S, Ma J, Liu Y, Yu D: Inequality in Social Support Associated With Mild Cognitive Impairment: A Cross-Sectional Study of Older (>/=60 Years) Residents in Shanghai, China. *Front Public Health* 2021, 9:706322. |
| 119 | Social interactions between people with dementia: pilot evaluation of an observational instrument in a nursing home | Mabire JB, Gay MC, Vrignaud P, Garitte C, Vernooij-Dassen M. Social interactions between people with dementia: pilot evaluation of an observational instrument in a nursing home. Int Psychogeriatr. 2016 Jun;28(6):1005-15. doi: 10.1017/S1041610215002483 |
| 120 | Hearing Impairment, Loneliness, Social Isolation, and Cognitive Function: Longitudinal Analysis Using English Longitudinal Study on Ageing | Maharani A, Pendleton N, Leroi I. Hearing Impairment, Loneliness, Social Isolation, and Cognitive Function: Longitudinal Analysis Using English Longitudinal Study on Ageing. Am J Geriatr Psychiatry. 2019 Dec;27(12):1348-1356. doi: 10.1016/j.jagp.2019.07.010 |
| 121 | Relationship quality and sense of coherence in dementia: Results of a European cohort study | Marques MJ, Woods B, Hopper L, Jelley H, Irving K, Kerpershoek L, Meyer G, Bieber A, Stephan A, Sköldunger A, Sjölund BM, Selbaek G, Rosvik J, Zanetti O, Portolani E, de Vugt M, Verhey F, Gonçalves-Pereira M; Actifcare Consortium. Relationship quality and sense of coherence in dementia: Results of a European cohort study. Int J Geriatr Psychiatry. 2019 May;34(5):745-755. doi: 10.1002/gps.5082 |
| 122 | Trajectories of relationship quality in dementia: a longitudinal study in eight European countries | Marques MJ, Tan EYL, Woods B, Jelley H, Kerpershoek L, Hopper L, Irving K, Bieber A, Stephan A, Skoldunger A *et al*: Trajectories of relationship quality in dementia: a longitudinal study in eight European countries. *Aging Ment Health* 2022, 26(11):2307-2315 |
| 123 | Association between apathy and satisfaction with meaningful activities in older adults with mild cognitive impairment: A population‐based cross‐sectional study | Maruta M, Makizako H, Ikeda Y, Miyata H, Nakamura A, Han G, Shimokihara S, Tokuda K, Kubozono T, Ohishi M *et al*: Association between apathy and satisfaction with meaningful activities in older adults with mild cognitive impairment: A population-based cross-sectional study. *Int J Geriatr Psychiatry* 2021, 36(7):1065-1074 |
| 124 | The psychosocial health, experiences and needs of older adults and care partners during the first surge of the COVID-19 pandemic: a mixed-methods study | Marziliano A, Burns E, Pampanini T, Tom J, Ardito S, Ilyas A, Carney MT, Diefenbach MA, Makhnevich A, Sinvani L: The psychosocial health, experiences and needs of older adults and care partners during the first surge of the COVID-19 pandemic: a mixed-methods study. *BMC Geriatr* 2022, 22(1):752 |
| 125 | Anticipated Stigma and Dementia-Related Anxiety in Middle-Aged and Older Adults | Maxfield M, Greenberg J. Anticipated Stigma and Dementia-Related Anxiety in Middle-Aged and Older Adults. GeroPsych (Bern). 2021 Mar;34(1):13-22. doi: 10.1024/1662-9647/a000234 |
| 126 | The impact of subjective memory complaints on quality of life in community-dwelling older adults | Maki Y, Yamaguchi T, Yamagami T, Murai T, Hachisuka K, Miyamae F, Ito K, Awata S, Ura C, Takahashi R, Yamaguchi H. The impact of subjective memory complaints on quality of life in community-dwelling older adults. Psychogeriatrics. 2014 Sep;14(3):175-81. doi: 10.1111/psyg.12056 |
| 127 | Patient and companion shared decision making and satisfaction with decisions about starting cholinesterase medication at dementia diagnosis | McCabe R, Pavlickova H, Xanthopoulou P, Bass NJ, Livingston G, Dooley J. Patient and companion shared decision making and satisfaction with decisions about starting cholinesterase medication at dementia diagnosis. Age Ageing. 2019 Sep 1;48(5):711-718. doi: 10.1093/ageing/afz045 |
| 128 | Decision-making involvement scale for individuals with dementia and family caregivers | Menne HL, Tucke SS, Whitlatch CJ, Feinberg LF. Decision-making involvement scale for individuals with dementia and family caregivers. Am J Alzheimers Dis Other Demen. 2008 Feb-Mar;23(1):23-9. doi: 10.1177/1533317507308312 |
| 129 | Cognition and adaptive functioning in older people attending drug and alcohol services | Monds LA, Ridley NJ, Rivas C, Withall A, Draper B, Lintzeris N. Cognition and adaptive functioning in older people attending drug and alcohol services. Int Psychogeriatr. 2017 May;29(5):815-823. doi: 10.1017/S1041610216002428 |
| 130 | The relationship between loneliness and cognition in healthy older men and women: The role of cortisol | Montoliu T, Hidalgo V, Salvador A. The relationship between loneliness and cognition in healthy older men and women: The role of cortisol. Psychoneuroendocrinology. 2019 Sep;107:270-279. doi: 10.1016/j.psyneuen.2019.05.024 |
| 131 | Quality of the caregiving relationship and quality of life in mild Alzheimer’s dementia | Mortazavizadeh Z, Maercker A, Roth T, Savaskan E, Forstmeier S. Quality of the caregiving relationship and quality of life in mild Alzheimer's dementia. Psychogeriatrics. 2020 Sep;20(5):568-577. doi: 10.1111/psyg.12546 |
| 132 | Capacity to consent to treatment: Empirical comparison of three instruments in older adults with and without dementia | Moye J, Karel MJ, Azar AR, Gurrera RJ. Capacity to consent to treatment: empirical comparison of three instruments in older adults with and without dementia. Gerontologist. 2004 Apr;44(2):166-75. doi: 10.1093/geront/44.2.166 |
| 133 | Neuropsychological predictors of decision-making capacity over 9 months in mild-to-moderate dementia | Moye J, Karel MJ, Gurrera RJ, Azar AR. Neuropsychological predictors of decision-making capacity over 9 months in mild-to-moderate dementia. J Gen Intern Med. 2006 Jan;21(1):78-83. doi: 10.1111/j.1525-1497.2005.00288.x |
| 134 | Comparison of three different assessments of capacity to consent in dementia patients | Mueller T, Haberstroh J, Knebel M, Oswald F, Weygandt M, Schröder J, Markwort S, Pantel J. Comparison of three different assessments of capacity to consent in dementia patients. GeroPsych (2015), 28, pp. 21-29. doi: 10.1024/1662-9647/a000119 |
| 135 | Prevention of cognitive and physical decline by enjoyable walking-habituation program based on brain-activating rehabilitation | Murai T, Yamaguchi T, Maki Y, Isahai M, Kaiho Sato A, Yamagami T, Ura C, Miyamae F, Takahashi R, Yamaguchi H. Prevention of cognitive and physical decline by enjoyable walking-habituation program based on brain-activating rehabilitation. Geriatr Gerontol Int. 2016 Jun;16(6):701-8. doi: 10.1111/ggi.12541 |
| 136 | Frailty as a Moderator of the Relationship between Social Isolation and Health Outcomes in Community-Dwelling Older Adults | Mehrabi F, Beland F: Frailty as a Moderator of the Relationship between Social Isolation and Health Outcomes in Community-Dwelling Older Adults. *Int J Environ Res Public Health* 2021, 18(4) |
| 137 | The Relationship Between Social Support and Subjective Cognitive Functioning Across Adulthood | Mueller A, Zucchetto JM, Siedlecki KL: The Relationship Between Social Support and Subjective Cognitive Functioning Across Adulthood. Int J Aging Hum Dev 2023, 96(2):174-200. |
| 138 | Neighborhood Social Cohesion and Dementia-Related Stigma Among Mothers of Adolescents in the Pre- and Current COVID-19 Period: An Observational Study Using Population-Based Cohort Data | Nakanishi M, Yamasaki S, Ando S, Endo K, Richards M, Hiraiwa-Hasegawa M, Kasai K, Nishida A: Neighborhood Social Cohesion and Dementia-Related Stigma Among Mothers of Adolescents in the Pre- and Current COVID-19 Period: An Observational Study Using Population-Based Cohort Data. *J Alzheimers Dis* 2022, 88(2):493-502 |
| 139 | Awareness of social and emotional functioning in people with early-stage dementia and implications for carers | Nelis SM, Clare L, Martyr A, Markova I, Roth I, Woods RT, Whitaker CJ, Morris RG. Awareness of social and emotional functioning in people with early-stage dementia and implications for carers. Aging Ment Health. 2011 Nov;15(8):961-9. doi: 10.1080/13607863.2011.575350 |
| 140 | Depression and loneliness/social isolation among patients with cognitive impairment in nursing home | Nikmat AW, Hashim NA, Omar SA, Razali S. Depression And Loneliness/Social Isolation Among Patients With Cognitive Impairment In Nursing Home ASEAN Journal of Psychiatry, Vol. 16 (2), July - December 2015. 222-231. |
| 141 | The Association between Social Support Sources and Cognitive Function among Community-Dwelling Older Adults: A One-Year Prospective Study | Noguchi T, Nojima I, Inoue-Hirakawa T, Sugiura H. The Association between Social Support Sources and Cognitive Function among Community-Dwelling Older Adults: A One-Year Prospective Study. Int J Environ Res Public Health. 2019 Oct 31;16(21):4228. doi: 10.3390/ijerph16214228 |
| 142 | Different dimensions of social support differentially predict psychological well-being in late life: opposite effects of perceived emotional support and marital status on symptoms of anxiety and of depression in older outpatients in Italy | Nicolini P, Abbate C, Inglese S, Rossi PD, Mari D, Cesari M: Different dimensions of social support differentially predict psychological well-being in late life: opposite effects of perceived emotional support and marital status on symptoms of anxiety and of depression in older outpatients in Italy. *Psychogeriatrics* 2021, 21(1):42-53 |
| 143 | Establishment of the Japanese version of the dementia stigma assessment scale | Noguchi T, Shang E, Nakagawa T, Komatsu A, Murata C, Saito T: Establishment of the Japanese version of the dementia stigma assessment scale. *Geriatr Gerontol Int* 2022, 22(9):790-796. |
| 144 | Social adaptive functioning, apathy, and nondysphoric depression among nursing home-dwelling very old adults | Onwuameze OE, Paradiso S. Social adaptive functioning, apathy, and nondysphoric depression among nursing home-dwelling very old adults. Psychopathology. 2014;47(5):319-26. doi: 10.1159/000360823 |
| 145 | Social support availability is positively associated with memory in persons aged 45‐85 years: A cross-sectional analysis of the Canadian Longitudinal Study on Aging | Oremus M, Tyas SL, Maxwell CJ, Konnert C, O'Connell ME, Law J. Social support availability is positively associated with memory in persons aged 45-85 years: A cross-sectional analysis of the Canadian Longitudinal Study on Aging. Arch Gerontol Geriatr. 2020 Jan-Feb;86:103962. doi: 10.1016/j.archger.2019.103962 |
| 146 | Medical decision-making capacity in patients with mild cognitive impairment | Okonkwo O, Griffith HR, Belue K, Lanza S, Zamrini EY, Harrell LE, Brockington JC, Clark D, Raman R, Marson DC. Medical decision-making capacity in patients with mild cognitive impairment. Neurology. 2007 Oct 9;69(15):1528-35. doi: 10.1212/01.wnl.0000277639.90611.d9 |
| 147 | Association of Low Emotional and Tangible Support With Risk of Dementia Among Adults 60 Years and Older in South Korea | Oh DJ, Yang HW, Kim TH, Kwak KP, Kim BJ, Kim SG, Kim JL, Moon SW, Park JH, Ryu SH *et al*: Association of Low Emotional and Tangible Support With Risk of Dementia Among Adults 60 Years and Older in South Korea. *JAMA Netw Open* 2022, 5(8):e2226260 |
| 148 | Proxy evaluation of dignity expectations and satisfaction of older patients with dementia by family members and nurses | Otake E, Ota K, Ikegami C, Niimi Y, Yamada S, Maeda J, Matsuda M: Proxy evaluation of dignity expectations and satisfaction of older patients with dementia by family members and nurses. *Nurs Open* 2021, 8(6):3120-3134 |
| 149 | Predictors of financial capacity performance in older adults using the Financial Competence Assessment Inventory | Pachana NA, Byrne GJ, Wilson J, Tilse C, Pinsker DM, Massavelli B, Vearncombe KJ, Mitchell LK. Predictors of financial capacity performance in older adults using the Financial Competence Assessment Inventory. Int Psychogeriatr. 2014 Jun;26(6):921-7. doi: 10.1017/S1041610214000209 |
| 150 | Assessment of capacity to consent to research among older persons with schizophrenia, Alzheimer disease, or diabetes mellitus: comparison of a 3-item questionnaire with a comprehensive standardized capacity instrument | Palmer BW, Dunn LB, Appelbaum PS, Mudaliar S, Thal L, Henry R, Golshan S, Jeste DV. Assessment of capacity to consent to research among older persons with schizophrenia, Alzheimer disease, or diabetes mellitus: comparison of a 3-item questionnaire with a comprehensive standardized capacity instrument. Arch Gen Psychiatry. 2005 Jul;62(7):726-33. doi: 10.1001/archpsyc.62.7.726 |
| 151 | Predicting loneliness in old people living in the community | Paúl C, Ribeiro O. Predicting loneliness in old people living in the community. Reviews in Clinical Gerontology. 2009. 19(1). 53-60. doi:10.1017/S0959259809990074 |
| 152 | Predictors of loneliness during the Covid-19 pandemic in people with dementia and their carers in England: findings from the DETERMIND-C19 study | Perach R, Read S, Hicks B, Harris PR, Rusted J, Brayne C, Dangoor M, Miles E, Dixon J, Robinson L *et al*: Predictors of loneliness during the Covid-19 pandemic in people with dementia and their carers in England: findings from the DETERMIND-C19 study. *Aging Ment Health* 2023, 27(3):521-532 |
| 153 | Predictors of change over time in subjective daytime sleepiness among older adult recipients of long-term services and supports | Petrovsky DV, Hirschman KB, Varrasse McPhillips M, Sefcik JS, Hanlon AL, Huang L, Brewster GS, Hodgson NA, Naylor MD. Predictors of change over time in subjective daytime sleepiness among older adult recipients of long-term services and supports. Int Psychogeriatr. 2020 Jul;32(7):849-861. doi: 10.1017/S1041610220000782 |
| 154 | Association between recognition and help-seeking preferences and stigma towards people with mental illness | Picco L, Abdin E, Pang S, Vaingankar JA, Jeyagurunathan A, Chong SA, Subramaniam M. Association between recognition and help-seeking preferences and stigma towards people with mental illness. Epidemiol Psychiatr Sci. 2018 Feb;27(1):84-93. doi: 10.1017/S2045796016000998 |
| 155 | The differential relationships of dimensions of perceived social support with cognitive function among older adults | Pillemer SC, Holtzer R. The differential relationships of dimensions of perceived social support with cognitive function among older adults. Aging Ment Health. 2016 Jul;20(7):727-35. doi: 10.1080/13607863.2015.1033683 |
| 156 | Gender-stratified analyses reveal longitudinal associations between social support and cognitive decline in older men | Pillemer S, Ayers E, Holtzer R. Gender-stratified analyses reveal longitudinal associations between social support and cognitive decline in older men. Aging Ment Health. 2019 Oct;23(10):1326-1332. doi: 10.1080/13607863.2018.1495178 |
| 157 | Exploitation in older adults: personal competence correlates of social vulnerability | Pinsker DM, McFarland K. Exploitation in older adults: personal competence correlates of social vulnerability. Neuropsychol Dev Cogn B Aging Neuropsychol Cogn. 2010 Nov;17(6):673-708. doi: 10.1080/13825585.2010.501403 |
| 158 | Social Vulnerability Scale for older adults: Validation study | Pinsker DM, Stone V, Pachana N, Greenspan S. Social Vulnerability Scale for older adults: Validation study. Clinical Psychologist, Vol. 10, No. 3, November 2006, 109-119. doi: 10.1080/13284200600939918 |
| 159 | Sense of purpose as a potential buffer between mental health and subjective cognitive decline | Pfund GN, Spears I, Norton SA, Bogdan R, Oltmanns TF, Hill PL: Sense of purpose as a potential buffer between mental health and subjective cognitive decline. *Int Psychogeriatr* 2022, 34(12):1045-1055 |
| 160 | Associations Among Loneliness, Purpose in Life and Subjective Cognitive Decline in Ethnoracially Diverse Older Adults Living in the United States | Pluim CF, Anzai JAU, Martinez JE, Munera D, Garza-Naveda AP, Vila-Castelar C, Guzman-Velez E, Ramirez-Gomez L, Bustin J, Serrano CM *et al*: Associations Among Loneliness, Purpose in Life and Subjective Cognitive Decline in Ethnoracially Diverse Older Adults Living in the United States. *J Appl Gerontol* 2023, 42(3):376-386 |
| 161 | Association of social support with mild cognitive impairment and dementia among older women: the Women’s Health Initiative Memory Study | Posis AIB, Yarish NM, McEvoy LK, Jain P, Kroenke CH, Saquib N, Ikramuddin F, Schnatz PF, Bellettiere J, Rapp SR *et al*: Association of Social Support with Mild Cognitive Impairment and Dementia Among Older Women: The Women's Health Initiative Memory Study. *J Alzheimers Dis* 2023, 91(3):1107-1119. |
| 162 | Association between social functioning and prefrontal hemodynamic responses in elderly adults | Pu S, Nakagome K, Yamada T, Yokoyama K, Matsumura H, Mitani H, Adachi A, Kaneko K. Association between social functioning and prefrontal hemodynamic responses in elderly adults. Behav Brain Res. 2014 Oct 1;272:32-9. doi: 10.1016/j.bbr.2014.06.052 |
| 163 | Social factors that predict cognitive decline in older African American adults | Pugh E, De Vito A, Divers R, Robinson A, Weitzner DS, Calamia M: Social factors that predict cognitive decline in older African American adults. Int J Geriatr Psychiatry 2021, 36(3):403-410. |
| 164 | The Association Between Social Engagement, Mild Cognitive Impairment, and Falls Among Older Primary Care Patients | Quach LT, Ward RE, Pedersen MM, Leveille SG, Grande L, Gagnon DR, Bean JF. The Association Between Social Engagement, Mild Cognitive Impairment, and Falls Among Older Primary Care Patients. Arch Phys Med Rehabil. 2019 Aug;100(8):1499-1505. doi: 10.1016/j.apmr.2019.01.020 |
| 165 | Loneliness, Social Integration, and Incident Dementia Over 6 Years: Prospective Findings From the English Longitudinal Study of Ageing | Rafnsson SB, Orrell M, d'Orsi E, Hogervorst E, Steptoe A. Loneliness, Social Integration, and Incident Dementia Over 6 Years: Prospective Findings From the English Longitudinal Study of Ageing. J Gerontol B Psychol Sci Soc Sci. 2020 Jan 1;75(1):114-124. doi: 10.1093/geronb/gbx087 |
| 166 | Disability but not social support predicts cognitive deterioration in late-life depression | Riddle M, McQuoid DR, Potter GG, Steffens DC, Taylor WD. Disability but not social support predicts cognitive deterioration in late-life depression. Int Psychogeriatr. 2015 May;27(5):707-14. doi: 10.1017/S1041610214002543 |
| 167 | The impact of relationship quality on life satisfaction and well-being in dementia caregiving dyads: findings from the IDEAL study | Rippon I, Quinn C, Martyr A, Morris R, Nelis SM, Jones IR, Victor CR, Clare L. The impact of relationship quality on life satisfaction and well-being in dementia caregiving dyads: findings from the IDEAL study. Aging Ment Health. 2020 Sep;24(9):1411-1420. doi: 10.1080/13607863.2019.1617238 |
| 168 | The moderating effect of cognitive function on the association between social support and depressive symptoms among community-dwelling older adults: Cross-sectional and longitudinal analyses | Roh HW, Cho EJ, Son SJ, Hong CH: The moderating effect of cognitive function on the association between social support and depressive symptoms among community-dwelling older adults: Cross-sectional and longitudinal analyses. *J Affect Disord* 2022, 318:185-190 |
| 169 | Changes in Social Network Size Are Associated With Cognitive Changes in the Oldest-Old | Rohr S, Lobner M, Guhne U, Heser K, Kleineidam L, Pentzek M, Fuchs A, Eisele M, Kaduszkiewicz H, Konig HH *et al*: Changes in Social Network Size Are Associated With Cognitive Changes in the Oldest-Old. *Front Psychiatry* 2020, 11:330 |
| 170 | Association of Social Support with Brain Volume and Cognition | Salinas J, O'Donnell A, Kojis DJ, Pase MP, DeCarli C, Rentz DM, Berkman LF, Beiser A, Seshadri S: Association of Social Support With Brain Volume and Cognition. *JAMA Netw Open* 2021, 4(8):e2121122 |
| 171 | Promoting Independence in Dementia (PRIDE): protocol for a feasibility randomised controlled trial | Shafayat A, Csipke E, Bradshaw L, Charlesworth G, Day F, Leung P, Moniz-Cook E, Montgomery AA, Morris S, Mountain G, Ogollah R, Sprange K, Yates L, Orrell M. Promoting Independence in Dementia (PRIDE): protocol for a feasibility randomised controlled trial. Trials. 2019 Dec 11;20(1):709. doi: 10.1186/s13063-019-3838-x |
| 172 | Emotional loneliness is associated with a risk of dementia in a general Japanese older population: the Hisayama Study | Shibata M, Ohara T, Hosoi M, Hata J, Yoshida D, Hirabayashi N, Morisaki Y, Nakazawa T, Mihara A, Nagata T *et al*: Emotional Loneliness Is Associated With a Risk of Dementia in a General Japanese Older Population: The Hisayama Study. *J Gerontol B Psychol Sci Soc Sci* 2021, 76(9):1756-1766 |
| 173 | Social networks and cognitive function in older adults receiving home- and community-based aged care | Siette J, Georgiou A, Brayne C, Westbrook JI. Social networks and cognitive function in older adults receiving home- and community-based aged care. Arch Gerontol Geriatr. 2020 Jul-Aug;89:104083. doi: 10.1016/j.archger.2020.104083 |
| 174 | Distinct Functions of Social Support and Cognitive Function Among Older Adults | Sims RC, Hosey M, Levy SA, Whitfield KE, Katzel LI, Waldstein SR. Distinct functions of social support and cognitive function among older adults. Exp Aging Res. 2014;40(1):40-59. doi: 10.1080/0361073X.2014.857551 |
| 175 | The influence of functional social support on executive functioning in middle-aged African Americans | Sims RC, Levy SA, Mwendwa DT, Callender CO, Campbell AL Jr. The influence of functional social support on executive functioning in middle-aged African Americans. Neuropsychol Dev Cogn B Aging Neuropsychol Cogn. 2011 Jul;18(4):414-31. doi: 10.1080/13825585.2011.567325 |
| 176 | Self-reported pain in persons with dementia predicts subsequent decreased psychosocial functioning | Snow AL, Chandler JF, Kunik ME, Davila JA, Balasubramanyam V, Steele AB, Morgan RO. Self-reported pain in persons with dementia predicts subsequent decreased psychosocial functioning. Am J Geriatr Psychiatry. 2009 Oct;17(10):873-80. doi: 10.1097/JGP.0b013e3181ad4f73 |
| 177 | Evaluating the Influence of Social Engagement on Cognitive Impairment and Mobility Outcomes Within the Boston RISE Cohort Study | Steere HK, Quach L, Grande L, Bean JF. Evaluating the Influence of Social Engagement on Cognitive Impairment and Mobility Outcomes Within the Boston RISE Cohort Study. Am J Phys Med Rehabil. 2019 Aug;98(8):685-691. doi: 10.1097/PHM.0000000000001175 |
| 178 | Correlates of healthcare and financial decision making among older adults without dementia | Stewart CC, Yu L, Wilson RS, Bennett DA, Boyle PA. Correlates of healthcare and financial decision making among older adults without dementia. Health Psychol. 2018 Jul;37(7):618-626. doi: 10.1037/hea0000610 |
| 179 | Social determinants of memory change: A three-year follow-up of the Canadian Longitudinal Study on Aging (CLSA) | Stinchcombe A, Hammond NG: Social determinants of memory change: A three-year follow-up of the Canadian Longitudinal Study on Aging (CLSA). *Arch Gerontol Geriatr* 2023, 104:104830 |
| 180 | The relative contributions of biomarkers, disease modifying treatment, and dementia severity to Alzheimer’s stigma: A vignette-based experiment | Stites SD, Gill J, Largent EA, Harkins K, Sankar P, Krieger A, Karlawish J: The relative contributions of biomarkers, disease modifying treatment, and dementia severity to Alzheimer's stigma: A vignette-based experiment. *Soc Sci Med* 2022, 292:114620 |
| 181 | Psychometric Properties and Factor Analysis of the Engagement and Independence in Dementia Questionnaire (EID-Q) | Stoner CR, Orrell M, Spector A. Psychometric Properties and Factor Analysis of the Engagement and Independence in Dementia Questionnaire (EID-Q). Dement Geriatr Cogn Disord. 2018;46(3-4):119-127. doi: 10.1159/000488484 |
| 182 | Cognitive predictors of medical decision-making capacity in mild cognitive impairment and Alzheimer’s disease | Stormoen S, Almkvist O, Eriksdotter M, Sundström E, Tallberg IM. Cognitive predictors of medical decision-making capacity in mild cognitive impairment and Alzheimer's disease. Int J Geriatr Psychiatry. 2014 Dec;29(12):1304-11. doi: 10.1002/gps.4114 |
| 183 | Decisions and attitudes regarding participation and proxy in clinical trials among patients with impaired cognitive function | Stormoen S, Tallberg IM, Almkvist O, Eriksdotter M, Sundström E. Decisions and attitudes regarding participation and proxy in clinical trials among patients with impaired cognitive function. Dementia (London). 2019 Aug;18(6):2049-2061. doi: 10.1177/1471301217737413 |
| 184 | The Development of the Social Functioning Scale for Patients with Parkinson’s Disease | Su FT, Tai CH, Tan CH, Hwang WJ, Yu RL: The Development of the Social Functioning Scale for Patients with Parkinson's Disease. *J Parkinsons Dis* 2020, 10(3):1143-1151 |
| 185 | Prevalence, overlap, and interrelationships of physical, cognitive, psychological, and social frailty among community-dwelling older people in Japan | Sugie M, Harada K, Nara M, Kugimiya Y, Takahashi T, Kitagou M, Kim H, Kyo S, Ito H: Prevalence, overlap, and interrelationships of physical, cognitive, psychological, and social frailty among community-dwelling older people in Japan. *Arch Gerontol Geriatr* 2022, 100:104659 |
| 186 | Importance of loneliness in behavioral and psychological symptoms of dementia | Sun W, Matsuoka T, Oba H, Narumoto J: Importance of loneliness in behavioral and psychological symptoms of dementia. *Int J Geriatr Psychiatry* 2021, 36(4):540-546. |
| 187 | Loneliness and Risk of Dementia | Sutin AR, Stephan Y, Luchetti M, Terracciano A. Loneliness and Risk of Dementia. J Gerontol B Psychol Sci Soc Sci. 2020 Aug 13;75(7):1414-1422. doi: 10.1093/geronb/gby112 |
| 188 | Malnutrition risk and its association with appetite, functional and psychosocial status among elderly Malays in an agricultural settlement | Suzana S, Boon PC, Chan PP, Normah CD. Malnutrition risk and its association with appetite, functional and psychosocial status among elderly Malays in an agricultural settlement. Malays J Nutr. 2013 Apr;19(1):65-75. PMID: 24800385 |
| 189 | Handgrip strength as a predictor of functional, psychological and social health. A prospective population-based study among the oldest old | Taekema DG, Gussekloo J, Maier AB, Westendorp RG, de Craen AJ. Handgrip strength as a predictor of functional, psychological and social health. A prospective population-based study among the oldest old. Age Ageing. 2010 May;39(3):331-7. doi: 10.1093/ageing/afq022 |
| 190 | Investigating medical decision-making capacity in patients with cognitive impairment using a protocol based on linguistic features | Tallberg IM, Stormoen S, Almkvist O, Eriksdotter M, Sundström E. Investigating medical decision-making capacity in patients with cognitive impairment using a protocol based on linguistic features. Scand J Psychol. 2013 Oct;54(5):386-92. doi: 10.1111/sjop.12068 |
| 191 | Loneliness, depression, social support, and quality of life in older chronically ill Appalachians | Theeke LA, Goins RT, Moore J, Campbell H. Loneliness, depression, social support, and quality of life in older chronically ill Appalachians. J Psychol. 2012 Jan-Apr;146(1-2):155-71. doi: 10.1080/00223980.2011.609571 |
| 192 | Psychological distress and its correlates in older care-dependent persons living at home | Thygesen E, Saevareid HI, Lindstrom TC, Engedal K. Psychological distress and its correlates in older care-dependent persons living at home. Aging Ment Health. 2009 May;13(3):319-27. doi: 10.1080/13607860802534591 |
| 193 | Trends across 20 years in multiple indicators of functioning among older adults in the Netherlands | Timmermans EJ, Hoogendijk EO, Broese van Groenou MI, Comijs HC, van Schoor NM, Thomése FCF, Visser M, Deeg DJH, Huisman M. Trends across 20 years in multiple indicators of functioning among older adults in the Netherlands. Eur J Public Health. 2019 Dec 1;29(6):1096-1102. doi: 10.1093/eurpub/ckz065 |
| 194 | Association of depression and loneliness with specific cognitive performance in non-demented elderly males | Tzang RF, Yang AC, Yeh HL, Liu ME, Tsai SJ. Association of depression and loneliness with specific cognitive performance in non-demented elderly males. Med Sci Monit. 2015 Jan 9;21:100-4. doi: 10.12659/MSM.891086 |
| 195 | Social engagement and depressive symptoms of elderly residents with dementia: a cross-sectional study of 37 long-term care units | van Beek AP, Frijters DH, Wagner C, Groenewegen PP, Ribbe MW. Social engagement and depressive symptoms of elderly residents with dementia: a cross-sectional study of 37 long-term care units. Int Psychogeriatr. 2011 May;23(4):625-33. doi: 10.1017/S1041610210002061 |
| 196 | The effects of small-scale, homelike facilities for older people with dementia on residents, family caregivers and staff: design of a longitudinal, quasi-experimental study | Verbeek H, van Rossum E, Zwakhalen SM, Ambergen T, Kempen GI, Hamers JP. The effects of small-scale, homelike facilities for older people with dementia on residents, family caregivers and staff: design of a longitudinal, quasi-experimental study. BMC Geriatr. 2009 Jan 20;9:3. doi: 10.1186/1471-2318-9-3 |
| 197 | Effects of small-scale, home-like facilities in dementia care on residents’ behavior, and use of physical restraints and psychotropic drugs: a quasi-experimental study | Verbeek H, Zwakhalen SM, van Rossum E, Ambergen T, Kempen GI, Hamers JP. Effects of small-scale, home-like facilities in dementia care on residents' behavior, and use of physical restraints and psychotropic drugs: a quasi-experimental study. Int Psychogeriatr. 2014 Apr;26(4):657-68. doi: 10.1017/S1041610213002512 |
| 198 | Screening for cognitive impairment among the elderly attending the noncommunicable diseases clinics in a rural area of Punjab, North India | Verma M, Grover S, Singh T, Dahiya N, Nehra R: Screening for cognitive impairment among the elderly attending the noncommunicable diseases clinics in a rural area of Punjab, North India. *Asian J Psychiatr* 2020, 50:102001 |
| 199 | Prevalence and determinants of loneliness in people living with dementia: Findings from the IDEAL programme | Victor CR, Rippon I, Nelis SM, Martyr A, Litherland R, Pickett J, Hart N, Henley J, Matthews F, Clare L; IDEAL programme team. Prevalence and determinants of loneliness in people living with dementia: Findings from the IDEAL programme. Int J Geriatr Psychiatry. 2020 Aug;35(8):851-858. doi: 10.1002/gps.5305 |
| 200 | Fear of falling and cognitive impairment in elderly with different social support levels:findings from a community survey in Central Vietnam | Vo THM, Nakamura K, Seino K, Nguyen HTL, Van Vo T: Fear of falling and cognitive impairment in elderly with different social support levels: findings from a community survey in Central Vietnam. *BMC Geriatr* 2020, 20(1):141 |
| 201 | Association between loneliness and its components and cognitive function among older Chinese adults living in nursing homes: A mediation of depressive symptoms, anxiety symptoms, and sleep disturbances | Wang Q, Zan C, Jiang F, Shimpuku Y, Chen S: Association between loneliness and its components and cognitive function among older Chinese adults living in nursing homes: A mediation of depressive symptoms, anxiety symptoms, and sleep disturbances. *BMC Geriatr* 2022, 22(1):959 |
| 202 | Social capital and cognitive decline: Does sleep duration mediate the association? | Wang L, Li J, Wang Z, Du Y, Sun T, Na L, Niu Y: Social capital and cognitive decline: Does sleep duration mediate the association? *PLoS One* 2021, 16(5):e0252208 |
| 203 | Social support and subsequent cognitive frailty during a 1-year follow-up of older people: the mediating role of psychological distress | Wang Y, Li J, Fu P, Jing Z, Zhao D, Zhou C: Social support and subsequent cognitive frailty during a 1-year follow-up of older people: the mediating role of psychological distress. *BMC Geriatr* 2022, 22(1):162 |
| 204 | Interleukin-1 alpha (rs1800587) genetic polymorphism is associated with specific cognitive functions but not depression or loneliness in elderly males without dementia | Wang EH, Hong CJ, Yeh HL, Liou YJ, Yang AC, Liu ME, Tsai SJ. Interleukin-1 alpha (rs1800587) genetic polymorphism is associated with specific cognitive functions but not depression or loneliness in elderly males without dementia. Neurosci Lett. 2013 Nov 27;556:69-72. doi: 10.1016/j.neulet.2013.09.057 |
| 205 | Anxiety disorders and its risk factors among the Sichuan empty-nest older adults: a cross-sectional study | Wang Z, Shu D, Dong B, Luo L, Hao Q. Anxiety disorders and its risk factors among the Sichuan empty-nest older adults: a cross-sectional study. Arch Gerontol Geriatr. 2013 Mar-Apr;56(2):298-302. doi: 10.1016/j.archger.2012.08.016 |
| 206 | Montessori-based activities among persons with late-stage dementia: Evaluation of mental and behavioral health outcomes | Wilks SE, Boyd PA, Bates SM, Cain DS, Geiger JR. Montessori-Based Activities Among Persons with Late-Stage Dementia: Evaluation of Mental and Behavioral Health Outcomes. Dementia (London). 2019 May;18(4):1373-1392. doi: 10.1177/1471301217703242 |
| 207 | Constructing the social world: Impaired capacity for social simulation in dementia | Wilson NA, Ahmed RM, Hodges JR, Piguet O, Irish M. Constructing the social world: Impaired capacity for social simulation in dementia. Cognition. 2020 Sep;202:104321. doi: 10.1016/j.cognition.2020.104321 |
| 208 | Negative social interactions and risk of mild cognitive impairment in old age | Wilson RS, Boyle PA, James BD, Leurgans SE, Buchman AS, Bennett DA. Negative social interactions and risk of mild cognitive impairment in old age. Neuropsychology. 2015 Jul;29(4):561-70. doi: 10.1037/neu0000154 |
| 209 | A longitudinal exploration of mental health resilience, cognitive impairment and loneliness | Windle G, Hoare Z, Woods B, Huisman M, Burholt V: A longitudinal exploration of mental health resilience, cognitive impairment and loneliness. *Int J Geriatr Psychiatry* 2021, 36(7):1020-1028 |
| 210 | What contributes to a good quality of life in early dementia? Awareness and the QoL-AD: a cross-sectional study | Woods RT, Nelis SM, Martyr A, Roberts J, Whitaker CJ, Markova I, Roth I, Morris R, Clare L. What contributes to a good quality of life in early dementia? Awareness and the QoL-AD: a cross-sectional study. Health Qual Life Outcomes. 2014 Jun 11;12:94. doi: 10.1186/1477-7525-12-94 |
| 211 | The structure and functional correlates of social support networks of people in advanced old age living in chosen urban and rural areas in Poland: a cross‑sectional study | Wojszel ZB, Politynska B: The structure and functional correlates of social support networks of people in advanced old age living in chosen urban and rural areas in Poland: a cross-sectional study. *Eur J Ageing* 2021, 18(3):345-355 |
| 212 | Association of social isolation with health status among community-dwelling Chinese older adults living with homecare services: a cross-sectional survey in Hong Kong | Wong EL, Qiu H, Cheung AW, Leung HH, Chen FY, Yeoh EK: Association of social isolation with health status among community-dwelling Chinese older adults living with homecare services: a cross-sectional survey in Hong Kong. *Front Public Health* 2023, 11:1099734 |
| 213 | Perceived enactment of autonomy of nursing home residents: A German cross-sectional study | Wulff I, Kölzsch M, Kalinowski S, Kopke K, Fischer T, Kreutz R, Dräger D. Perceived enactment of autonomy of nursing home residents: A German cross-sectional study. Nurs Health Sci. 2013 Jun;15(2):186-93. doi: 10.1111/nhs.12016 |
| 214 | Loneliness, Sense of Control, and Risk of Dementia in Healthy Older Adults: A Moderated Mediation Analysis | Yang H, Tng GYQ, Ng WQ, Yang S: Loneliness, Sense of Control, and Risk of Dementia in Healthy Older Adults: A Moderated Mediation Analysis. *Clin Gerontol* 2021, 44(4):392-405 |
| 215 | The association among apathy, leisure activity participation, and severity of dementia in nursing home residents with Alzheimer’s disease: A cross-sectional study | Yang Y, Kwan RYC, Zhai HM, Xu XY, Huang CX, Liang SJ, Liu J: The association among apathy, leisure activity participation, and severity of dementia in nursing home residents with Alzheimer's disease: A cross-sectional study. *Geriatr Nurs* 2021, 42(6):1373-1378 |
| 216 | Exploring the bidirectional associations between loneliness and cognitive functioning over 10 years: the English longitudinal study of ageing | Yin J, Lassale C, Steptoe A, Cadar D. Exploring the bidirectional associations between loneliness and cognitive functioning over 10 years: the English longitudinal study of ageing. Int J Epidemiol. 2019 Dec 1;48(6):1937-1948. doi: 10.1093/ije/dyz085 |
| 217 | The Revised Index for Social Engagement in Long-Term Care Facilities: A Psychometric Study | Yoon JY, Kim H. The Revised Index for Social Engagement in Long-Term Care Facilities: A Psychometric Study. J Nurs Res. 2017 Jun;25(3):216-223. doi: 10.1097/JNR.0000000000000156 |
| 218 | Preferences for life-sustaining treatment in Korean adults: a cross-sectional study | Youn H, Lee SY, Jung HY, Kim SG, Kim SH, Jeong HG: Preferences for life-sustaining treatment in Korean adults: a cross-sectional study. *BMJ Open* 2021, 11(1):e039470 |
| 219 | Loneliness mediates the relationships between perceived neighborhood characteristics and cognition in middle‐aged and older adults | Yu X, Yang J, Yin Z, Jiang W, Zhang D: Loneliness mediates the relationships between perceived neighborhood characteristics and cognition in middle-aged and older adults. *Int J Geriatr Psychiatry* 2021, 36(12):1858-1866. |
| 220 | Longitudinal Assessment of the Relationships Between Geriatric Conditions and Loneliness | Yu K, Wu S, Jang Y, Chou CP, Wilber KH, Aranda MP, Chi I: Longitudinal Assessment of the Relationships Between Geriatric Conditions and Loneliness. *J Am Med Dir Assoc* 2021, 22(5):1107-1113 e1101 |
| 221 | A New Instrument Combines Cognitive and Social Functioning Items for Detecting Mild Cognitive Impairment and Dementia in Parkinson’s Disease | Yu YW, Tan CH, Su HC, Chien CY, Sung PS, Lin TY, Lee TL, Yu RL: A New Instrument Combines Cognitive and Social Functioning Items for Detecting Mild Cognitive Impairment and Dementia in Parkinson's Disease. *Front Aging Neurosci* 2022, 14:913958. |
| 222 | Perceived loneliness among older adults with mild cognitive impairment | Yu J, Lam CL, Lee TM. Perceived loneliness among older adults with mild cognitive impairment. Int Psychogeriatr. 2016 Oct;28(10):1681-5. doi: 10.1017/S1041610216000430 |
| 223 | Loneliness may mediate the relationship between depression and the quality of life among elderly with mild cognitive impairment | Zafar J, Malik NI, Atta M, Makhdoom IF, Ullah I, Manzar MD: Loneliness may mediate the relationship between depression and the quality of life among elderly with mild cognitive impairment. *Psychogeriatrics* 2021, 21(5):805-812 |
| 224 | The Characteristics of Social Network Structure in Later Life in Relation to Incidence of Mild Cognitive Impairment and Conversion to Probable Dementia | Zhang Y, Natale G, Clouston S: The Characteristics of Social Network Structure in Later Life in Relation to Incidence of Mild Cognitive Impairment and Conversion to Probable Dementia. *J Alzheimers Dis* 2021, 81(2):699-710 |
| 225 | Effects of exergaming on cognitive functions and loneliness of older adults with cognitive frailty | Zhu YZ, Lin CF, Yang HL, Jin G, Chiu HL: Effects of exergaming on cognitive functions and loneliness of older adults with cognitive frailty. *Int J Geriatr Psychiatry* 2023, 38(6):e5944 |
| 226 | Role of social support in cognitive function among elders | Zhu S, Hu J, Efird JT. Role of social support in cognitive function among elders. J Clin Nurs. 2012 Aug;21(15-16):2118-25. doi: 10.1111/j.1365-2702.2012.04178.x |
| 227 | Social support and verbal interaction are differentially associated with cognitive function in midlife and older age | Zuelsdorff ML, Koscik RL, Okonkwo OC, Peppard PE, Hermann BP, Sager MA, Johnson SC, Engelman CD. Social support and verbal interaction are differentially associated with cognitive function in midlife and older age. Neuropsychol Dev Cogn B Aging Neuropsychol Cogn. 2019 Mar;26(2):144-160. doi: 10.1080/13825585.2017.1414769 |
