# Supplementary material for: Instruments for assessing social health in the context of cognitive decline and dementia: a systematic review
Source: Front Psychiatry. 2024 Nov 13;15:1387192. doi: 10.3389/fpsyt.2024.1387192 (PMC11599264; doi:10.3389/fpsyt.2024.1387192)
Supplement: Supplementary file 4 [file Table4.docx]

Supplementary Table 4. Characteristics of included social health instruments (n=102), *(responsiveness=longitudinal validity, measurement of change).

| **No** | **Instrument**  **name** | **Author (First published)** | **Versions** | **No. items/data collection** | **Key**  **thematic concepts** | **Psycho-**  **metrics** | | | **Designed**  **for** | **Social health**  **domain(s)*** | | | | | | | | | | |
| --- | --- | --- | --- | --- | --- | --- | --- | --- | --- | --- | --- | --- | --- | --- | --- | --- | --- | --- | --- | --- |
|  |  |  |  |  |  | Reliability | Validity | Responsiveness* |  |  |  |  |  |  |  |  |  |  |  |  |
|  |  |  |  |  |  |  |  |  |  | Cap. (A1) | | Ind. (A2) | | SocP. (A3) | | Str. (B1) | | Func. (B2) | | Appr. (B3) |
| 1 | 2-Way Social Support Scale (2-Way SSS) | Shakespeare-Finch J & Obst PL (2011) [1] | - | 20/self-report | emotional support (receiving, giving), instrumental support (receiving, giving) | + | + | n.a. | General  population |  | |  | |  | |  | | X | |  |
| 2 | Anticipated Cost of Stigma Scale (ACSS) | Maxfield M & Green-berg J (2020) [2] | - | 7/self-report | being treated differently, excluded, criticized, having lower expectations, being judged, distrusted, viewed as weak | + | n.a. | n.a. | Older  population |  | |  | |  | |  | |  | | X |
| 3 | Arizona Social Support Interview Survey (ASSIS)  -expert group- | BarreraM (1980)[3] | - | 27/self-report | material aid, physical assistance, intimate interaction, guidance, feedback, social participation | + | n.a. | n.a. | General population |  | |  | |  | | X | | X | | X |
| 4 | Assessment of Negative Social Interactions | Krause N & Rook KS (2003) [4] | - | 12/self-report | neglect/rejection by others, unwanted intrusion/advice, failure by others to provide help, unsympathetic/insensitive behavior by others | + | n.a. | n.a. | Older population |  | |  | |  | |  | |  | | X |
| 5 | CollaboRATE  Scale | Elwyn G et al. (2013) [5] | - | 3/self-report | shared decision making: explanation of the health issue, elicitation of patient preferences, integration of patient preferences | + | + | n.a. | General population |  | | X | |  | |  | |  | |  |
| **No** | **Instrument**  **name** | **Author (First published)** | **Versions** | **No. items/data collec-tion** | **Key**  **thematic concepts** | **Psycho-**  **metrics** | | | **Designed**  **for** | **Social health**  **domain(s)*** | | | | | | | | | | |
|  |  |  |  |  |  | Reliability | Validity | Responsiveness |  | Cap. (A1) | | Ind. (A2) | | SocP. (A3) | | Str. (B1) | | Func. (B2) | | Appr. (B3) |
| 6 | Collaborative Research on Ageing in Europe Social Network Index (COURAGE-SNI)  -expert group- | Zawisza K et al. (2014)[6] | - | 5/self-report | structural and functional aspects of individuals’ social network | + | + | n.a. | Older population |  | |  | |  | | X | | X | |  |
| 7 | Capacity to Consent to Treatment Instrument (CCTI) | Marson DC et al. (1995)[7] | - | 5/self-report | consent abilities: decisional capacity, choice, personal consequences, reasoning, understanding | + | + | n.a. | Older population, dementia (mild, moderate) |  | | X | |  | |  | |  | |  |
| 8 | Competence Assessment Tool for Voting (CAT-V) | Appelbaum, PS et al. (2005)[8] | - | 6/self-report | capacity to vote: understanding, appreciation, reasoning, choice | + | + | n.a. | Dementia (mild, moderate, severe) |  | | X | |  | |  | |  | |  |
| 9 | Decisional Conflict Scale (DCS) | O’Connor, AM (1995)[9] | - | 4/self-report | level of comfort with a decision: uncertainty, factors contributing, effective-decision-making | + | + | n.a. | General population |  | | X | |  | |  | |  | |  |
| 10 | Decision-Making Involvement Scale (DMI) | Feinberg, LF et al. (2000)[10] | - | 15/self-report/ob-servation (proxy) | individual’s level of involvement in a variety of daily decisions (i.e. what to do with money, when to get up) | + | + | n.a. | Dementia |  | | X | |  | |  | |  | |  |
| 11 | De Jong Gierveld Loneliness Scale (6-item) | De Jong Giervel J & Van Tilburg TG (2006) [11] | 11-item version (De Jong Gierveld, 1985); Chinese version (Leung, 2008) | 6/self-report | emotional loneliness, social loneliness | + | + | n.a. | Older population |  | |  | |  | |  | |  | | X |
| **No** | **Instrument**  **name** | **Author (First published)** | **Versions** | **No. items/data collec-tion** | **Key**  **thematic concepts** | **Psycho-**  **metrics** | | | **Designed**  **for** | **Social health**  **domain(s)*** | | | | | | | | | | |
|  |  |  |  |  |  | Reliability | Validity | Responsiveness |  | Cap. (A1) | | Ind. (A2) | | SocP. (A3) | | Str. (B1) | | Func. (B2) | | Appr. (B3) |
| 12 | Dyadic Trust Scale (DTS) | Larzelere RE & Huston TL (1980) [12] | - | 8/self-report | feelings of interpersonal trust in a relationship | + | + | n.a. | General population |  | |  | |  | |  | |  | | X |
| 13 | Duke/UNC Functional Social Support Questionnaire (DUFSSQ)  -expert-group- | Broadhead WE (1988)[13] | - | 8/self-report | affective support, confidant support | + | + | n.a. | Older population |  | |  | |  | |  | | X | |  |
| 14 | Duke Social Support Index (DSSI-23) | Koenig HG (1993)[14] | 11-item version (Koenig, 1993); Persion version (Bayrami, M. et al. 2013) | 23/self-report | social interaction, subjective support, instrumental support | + | + | n.a. | Older population |  | |  | |  | | X | | X | | X |
| 15 | Engagement and In-dependence in Dementia Questionnaire (EID-Q) | Stoner CR et al. (2018)[15] | - | 26/self-report | activities of daily living, decision  making, activity engagement, support, reciprocity | + | + | n.a. | Dementia (mild) | X | | X | | X | |  | | X | | X |
| 16 | ENRICHD Social Support Inventory (ESSI)  -expert group- | ENRICHD investigators (2000) [16] | - | 7/self-report | emotional, instrumental, informational, appraisal | + | + | n.a. | Patients |  | |  | |  | |  | | X | |  |
| 17 | Evaluation to Sign Consent (ESC) | Resnick B et al. (2007)[17] | - | 5/self-report | potential risks, expectations, withdrawal, discomfort, treatment | + | n.a. | n.a. | Older population |  | | X | |  | |  | |  | |  |
| **No** | **Instrument**  **name** | **Author (First published)** | **Versions** | **No. items/data collec-tion** | **Key**  **thematic concepts** | **Psycho-**  **metrics** | | | **Designed**  **for** | **Social health**  **domain(s)*** | | | | | | | | | | |
|  |  |  |  |  |  | Reliability | Validity | Responsiveness |  | Cap. (A1) | | Ind. (A2) | | SocP. (A3) | | Str. (B1) | | Func. (B2) | | Appr. (B3) |
| 18 | Family Emotional Involvement and Criticism Scale (FEICS) | Shields CG et al. (1992)[18] | Chinese modification (Leung et al., 2007) | 14/self-report | perceived criticism,  intensity of emotional involvement | + | + | n.a. | General population |  | |  | |  | |  | |  | | X |
| 19 | Friendship Scale (FS) | Hawthorne G (2006) [19] | - | 6/self-report | feelings of loneliness, importance of actual social contacts | + | + | n.a. | Older population |  | |  | |  | |  | |  | | X |
| 20 | German Social Support Questionnaire-Short version | Frydrich T et al. (2009)[20] | - | 14/self-report | perceived social support | + | + | n.a. | Older population |  | |  | |  | |  | | X | |  |
| 21 | Health Care Empowerment Questionnaire (HCEQ) | Gagnon M et al. (2006) [21] | - | 10/self-report | patient information seeking, healthcare interaction results, degree of control | + | n.a. | n.a. | Older population |  | | X | |  | |  | |  | |  |
| 22 | Hertz Perceived Enactment of Autonomy Scale (HPEAS) | Hertz JE et al. (1991) [22] | - | 31/self-report | voluntariness, individuality, self-direction | + | + | n.a. | Older population |  | | X | |  | |  | |  | |  |
| 23 | International Mobility in Aging Study - Social Network Support Scale (IMIAS SNSS) | Phillips SP et al. (2016) [23] | - | 5/self-report | Social network, emotional support by social ties | + | + | n.a. | Older population |  | |  | |  | | X | | X | |  |
| 24 | Index of Social Engagement (ISE) | Mor V et al. (1995) [24] | - (look at 46. RISE) | 6/observation (proxy) | interacting with others, planned/structured activities, self-initiated activities, own goals, involvement in life of facility, invitations into group activities | + | + | n.a. | dementia (mild, moderate, severe) |  | |  | | X | |  | |  | |  |
| **No** | **Instrument**  **name** | **Author (First published)** | **Versions** | **No. items/data collec-tion** | **Key**  **thematic concepts** | **Psycho-**  **metrics** | | | **Designed**  **for** | **Social health**  **domain(s)*** | | | | | | | | | | |
|  |  |  |  |  |  | Reliability | Validity | Responsiveness |  | Cap. (A1) | Ind. (A2) | | SocP. (A3) | | Str. (B1) | | Func. (B2) | | Appr. (B3) | |
| 25 | Interpersonal Support Evaluation List (ISEL-40) | Cohen S & Hoberman H (1983)[25] | 12-item version (ISEL-12; Cohen & Hoberman, 1983)  -expert group- | 40/self-report | tangible support, appraisal support, self-esteem support, belonging support | + | + | n.a. | Older population |  | |  | |  | |  | | X | |  |
| 26 | Interview Schedule for Social Interaction (ISSI)  -expert group- | Hen-derson S et al. (1980) [26] | - | 50/self-report | availability of social interaction, adequacy of social interaction, availability of attachment, adequacy of attachment | + | + | n.a. | General population |  | |  | |  | |  | | X | | X |
| 27 | Linguistic Instrument for Medical Decision-Making (LIMD) | Tallberg IM et al. (2013)[27] | - | 3/obser-vation (proxy) | understanding the content, ability of evaluating risk and benefit, ability to express a decision | + | + | n.a. | Older popu-lation, MCI, dementia |  | | X | |  | |  | |  | |  |
| 28 | Lubben Social Network Scale (LSNS-6) | Lubben J et al. (2006) [28] | 12-item version (Lubben, 1988); Japanese Version (Kurimoto A. et al. 2011); Lubben Social Network Scale Revised (LSNS-R, Lubben, J. E., Gironda, M. W. 2003) | 6/self-report | family, friends (size, help, discussing private matters) | + | + | n.a. | Older population |  | |  | |  | | X | | X | |  |
| **No** | **Instrument**  **name** | **Author (First published)** | **Versions** | **No. items/data collec-tion** | **Key**  **thematic concepts** | **Psycho-**  **metrics** | | | **Designed**  **for** | **Social health**  **domain(s)*** | | | | | | | | | | |
|  |  |  |  |  |  | Reliability | Validity | Responsiveness |  | Cap. (A1) | | Ind. (A2) | | SocP. (A3) | | Str. (B1) | | Func. (B2) | | Appr. (B3) |
| 29 | MacArthur Competence Assessment Tool for Clinical Research (MacCAT-CR) | Appelbaum PS & Grisso T (2001) [29] | MacArthur Competence Assessment Tool for Treatment (MacCAT-T; Grisso & Appelbau, 1997) | 13/self-report | understanding information, reasoning about participation, appreciation of effects of participation, expressing choice | + | + | n.a. | General population |  | | X | |  | |  | |  | |  |
| 30 | Marlowe-Crowne Social Desirability Scale - Short Form | Rey-nolds WM et al. (1982) [30] | 33-item version (Marlowe & Crowne, 1960) | 13/self-report | tendency to act socially desirable | + | + | n.a. | General population | X | |  | |  | |  | |  | |  |
| 31 | Medical Outcomes Study Social Support Survey (MOS-SSS) | Sherbourne CD et al. (1991) [31] | Brazilian Portuguese Adaptation (dos Santos, SB. et al. 2018) | 19/self-report | emotional/informational, tangible, affectionate, positive social interaction | + | + | n.a. | Older population |  | |  | |  | |  | | X | |  |
| 32 | Melbourne Decision Making Questionnaire (MDMQ) | Mann L et al. (1997) [32] | - | 22/self-report | vigilance, buck-passing, procrastination, hypervigilance | + | n.a. | n.a. | General population |  | | X | |  | |  | |  | |  |
| 33 | Minimum Data Set Social Engagement (SocE) Measure | Morris JN (1990) [33] | - | 6/ob-servation (proxy) | interactions, planned acts, self-act, own-goal, involvement, group-acts | + | + | n.a. | Nursing home residents |  | |  | | X | |  | |  | |  |
| **No** | **Instrument**  **name** | **Author (First published)** | **Versions** | **No. items/data collec-tion** | **Key**  **thematic concepts** | **Psycho-**  **metrics** | | | **Designed**  **for** | **Social health**  **domain(s)*** | | | | | | | | | | |
|  |  |  |  |  |  | Reliability | Validity | Responsiveness |  | Cap. (A1) | | Ind. (A2) | | SocP. (A3) | | Str. (B1) | | Func. (B2) | | Appr. (B3) |
| 34 | Modified Healthcare and Financial Decision-Making Measure | Boyle PA et al. (2013) [34] | - | 12/self-report | financial decision making, healthcare decision making | + | + | n.a. | Older population |  | | X | |  | |  | |  | |  |
| 35 | Multidimensional Scale of Perceived Social Support (MSPSS) | Zimet GD et al. (1988) [35] | - | 12/self-report | family, friends, significant others | + | n.a. | n.a. | General population |  | |  | |  | |  | | X | |  |
| 36 | Oslo Social Support Scale (OSSS) | Dalgard OS et al. (2006) [36] | - | 3/self-report | number of close persons, sense of concern/interest from others, easiness getting help from neighbours | + | + | n.a. | Older population |  | |  | |  | | X | | X | | X |
| 37 | Partnership Questionnaire (PQ) | Hahlweg K (1996) [37] | Short form of PFB-K (German version; Kliem et al., 2012) | 30/self-report | conflict behaviour, tenderness, commonality/communication, and overall satisfaction (separate item) | + | + | n.a. | General population |  | |  | |  | |  | |  | | X |
| 38 | Passivity in Dementia Scale (PDS) | Colling KB (2000) [38] | - | 9/observation (proxy) | interacting with people, interactions with staff, family, or other residents | + | + | n.a. | Dementia |  | |  | | X | |  | |  | |  |
| 39 | Patient Dignity Inventory (PDI) | Chochinov HM et al. (2008)[39] | - | 25/self-report | dignity-related distress: physical, functional, psychological, social, existential, spiritual | + | n.a. | n.a. | Palliative care patients | X | | X | |  | |  | |  | | X |
| 40 | Personal Resource Questionnaire (PRQ2000)  -expert group- | Weinert C (2000) [40] | - | 15/self-report | attachment/intimacy, social integration, nurturance, reassurance of worth, availability of assistance | + | + | n.a. | General population | X | |  | | X | | X | | X | | X |
| **No** | **Instrument**  **name** | **Author (First published)** | **Versions** | **No. items/data collec-tion** | **Key**  **thematic concepts** | **Psycho-**  **metrics** | | | **Designed**  **for** | **Social health**  **domain(s)*** | | | | | | | | | | |
|  |  |  |  |  |  | Reliability | Validity | Responsiveness |  | Cap. (A1) | | Ind. (A2) | | SocP. (A3) | | Str. (B1) | | Func. (B2) | | Appr. (B3) |
| 41 | Reintegration to Normal Living Index (RNLI) | Wood-Dauphinee S et al. (1988)[41] | - | 11/self-report | participation in daily social activities, perception of self | + | + | + | Older population |  | |  | | X | |  | |  | |  |
| 42 | Restorative Activity Questionnaire (RAQ) | Chiu YC et al. (2013) [42] | - | 58/ob-servation (proxy) | leisure participation, restorative evaluation, leisure dysfunction | + | n.a. | n.a. | Not specified |  | |  | | X | |  | |  | |  |
| 43 | Revised Index for Social Engagement (RISE) | Gerritsen DL et al. (2008)[43] | - | 6/obser-vation (proxy) | quality of social interaction, group involvement | + | n.a. | n.a. | Older population |  | |  | | X | |  | |  | | X |
| 44 | Pleasant Events Schedule-AD (PES-AD) short form | Logsdon RG & Teri L (1997) [44] | original 53-item version (Teri & Logsdon, 1991) | 20/self-report & observation (proxy) | frequency of participation in leisure activities, enjoyment  of participation in leisure activities | + | + | + | MCI/  Dementia |  | |  | | X | |  | |  | |  |
| 45 | Positive Affect Index (PAI) | Bengtson VL et al. (1982) [45] | - | 5/self-report | closeness, communication, similarity of views, shared activities, generally getting along | + | n.a. | n.a. | Older population |  | |  | |  | |  | |  | | X |
| 46 | Satisfaction with Decision (SWD) Scale | Holmes-Rovner M (1996) [46] | - | 6/self-report | satisfaction with information, satisfaction with decision, conformity of decision with one's own personal values | + | + | n.a. | General patient population |  | | X | |  | |  | |  | |  |
| 47 | Scale for Quality of the Current Relationship in Caregiving (SQCRC) | Spruytte N et al. (2000) [47] | German version (Mortazavizadeh et al., 2020) | 14/self-report & observation (proxy) | warmth/affection, conflict/criticism | + | + | n.a. | General population |  | |  | |  | |  | |  | | X |
| **No** | **Instrument**  **name** | **Author (First published)** | **Versions** | **No. items/data collec-tion** | **Key**  **thematic concepts** | **Psycho-**  **metrics** | | | **Designed**  **for** | **Social health**  **domain(s)*** | | | | | | | | | | |
|  |  |  |  |  |  | Reliability | Validity | Responsiveness |  | Cap. (A1) | | Ind. (A2) | | SocP. (A3) | | Str. (B1) | | Func. (B2) | | Appr. (B3) |
| 48 | Social Adaptation Self-Evaluation Scale (SASS) | Bosc M et al. (1997) [48] | - | 21/self-report | work and leisure, family and extra-family relationships, intellectual interest, satisfaction in roles, patient self-perception of his ability to manage and control his environment | + | + | + | General population | X | | X | |  | |  | |  | |  |
| 49 | Social-Adaptive Functioning Evaluation (SAFE) | Harvey PD et al. (1997) [49] | - | 17/observation (proxy) | social competence and adjustment, self-care, impulse control, cooperativeness and life skill functioning | + | + | n.a. | Schizophrenia patients | X | |  | |  | |  | |  | |  |
| 50 | Social Disconnectedness and Perceived Isolation (SDPI) Scale  -expert group- | Cornwell EY et al. (2009) [50] | - | 17/self-report | social network, no. of friends, social participation, loneliness, social isolation | + | + | n.a. | Older population |  | |  | |  | | X | |  | | X |
| 51 | Social Distance Scale (SDS) | Link B et al. (1987) [51] | - | 7/self-report | social distance, stigmatization | + | n.a. | n.a. | General population | X | |  | |  | |  | |  | | X |
| 52 | Social Functioning in Dementia Scale (SF-DEM)  -expert group- | Sommer-lad A et al. (2017) [52] | - | 20/self-report & observation (proxy) | spending time with other people, communicating with other people, and sensitivity to other people | + | + | n.a. | Dementia |  | |  | | X | | X | |  | |  |
| 53 | Social Interaction Scale (SIS) | Chen YL et al. (2000) [53] | - | 41/ob-ser-vation (proxy) | family/community interaction, institutional interaction | + | n.a. | n.a. | Nursing home residents |  | |  | | X | | X | |  | |  |
| 54 | Social Norms Questionnaire (SNQ) | Rankin K et al. (2008) [54] | - | 22/self-report | social rigidity/endorsement of a socially appropriate behaviour, endorsement of a socially inappropriate behaviour | n.a. | + | n.a. | Older population | X | |  | |  | |  | |  | |  |
| **No** | **Instrument**  **name** | **Author (First published)** | **Versions** | **No. items/data collec-tion** | **Key**  **thematic concepts** | **Psycho-**  **metrics** | | | **Designed**  **for** | **Social health**  **domain(s)*** | | | | | | | | | | |
|  |  |  |  |  |  | Reliability | Validity | Responsiveness |  | Cap. (A1) | | Ind. (A2) | | SocP. (A3) | | Str. (B1) | | Func. (B2) | | Appr. (B3) |
| 55 | Social Observation Behaviors Residents Index (SOBRI) | Mabire JB (2016) [55] | - | 126/ob-ser-vation (proxy) | social interactions with other residents, social interactions with care staff, self-centred behaviours, unclassifiable behaviours | + | n.a. | n.a. | Dementia |  | |  | | X | |  | |  | |  |
| 56 | Social Performance Survey (SPS) | Lowe MR & Cautela JR (1978) [56] | - | 100/self-report | appropriate social skills and communication skills, inappropriate assertion, sociopathic behaviour | + | + | n.a. | General population | X | |  | |  | |  | |  | |  |
| 57 | Social Problem-Solving Inventory-Revised (SPSI-R:L) | D’Zurilla N et al. (2002)[57] | 25-items version (SPSI-R:S; D’Zurilla, Nezu, Maydeu-Olivares, 2002); Spanish adaptation (Maydeu-Olivares et al., 2000) | 52/self-report | positive problem orientation, negative problem orientation, rational problem solving, impulsivity/carelessness style, avoidance style | + | n.a. | n.a. | Psychiatric inpatients | X | |  | |  | |  | |  | |  |
| 58 | Social Provisions Scale | Cutrona CE & Russell DW (1987) [58] | 10-item version (SPS-10; Caron, J., 2013, French); 5-item version (SPS-5; Orpana, Lang, Yurkowski, 2019) | 24/self-report | attachment, social integration, reassurance of worth, sense of reliable alliance, guidance, and opportunity for nurturance | + | + | n.a. | General population |  | |  | |  | |  | | X | | X |
| 59 | Social role subscale of the Late Life Function and Disability Instrument (LLFDI) | Jette AM et al. (2002) [59] | - | 9/self-report | in touch with others, visit friends and family, provide care to others, voluntary work, recreation/travel, inviting people, organized social activities | + | + | + | Older population | X | |  | | X | | X | |  | |  |
| **No** | **Instrument**  **name** | **Author (First published)** | **Versions** | **No. items/data collec-tion** | **Key**  **thematic concepts** | **Psycho-**  **metrics** | | | **Designed**  **for** | **Social health**  **domain(s)*** | | | | | | | | | | |
|  |  |  |  |  |  | Reliability | Validity | Responsiveness |  | Cap. (A1) | | Ind. (A2) | | SocP. (A3) | | Str. (B1) | | Func. (B2) | | Appr. (B3) |
| 60 | Social Support Questionnaire Short-Form (SSQ6) | Sarason IG et al. (1987) [60] | original 27-items version (Sarason et al., 1981); 3-item version (SSQ3, Sarason et al., 1987) | 6/self-report | network size, overall satisfaction with the support received | + | + | n.a. | General population |  | |  | |  | |  | | X | | X |
| 61 | Social Support Rating Scale (SSRS) | Lue BH et al. (1995) [61] | original Chinese version (Xiao, 1994) | 20/self-report | instrumental support, emotional support | + | + | n.a. | Older population |  | |  | |  | |  | | X | |  |
| 62 | Social Vulnerability Scale (SVS-15) | Pinsker DM (2010) [62] | 22-item version (SVS-22; Pinsker, 2006) | 15/observation (proxy) | tendency to unquestioningly believe things that are unlikely to be true (credulity), and susceptibility to exploitation (gullibility) | + | + | n.a. | Older population | X | |  | |  | |  | |  | |  |
| 63 | Socioemotional Dysfunction Scale (SDS) | Barsuglia JP (2014) [63] | - | 40/observation (proxy) | extraversion, warmth, social influence, insight, openness, appropriateness, and maladjustment. | + | + | n.a. | Dementia | X | |  | |  | |  | |  | |  |
| 64 | Socio-Emotional Questionnaire (SEQ) | Bramham J et al. (2009) [64] | - | 30/self-report & ob-servation (proxy) | emotion recognition, empathy, social conformity, antisocial behaviour, sociability | + | + | n.a. | Brain injury patients | X | |  | |  | |  | |  | |  |
| 65 | Stigma Experience Scales (SES) | Wahl OF et al. (1999) [65] | - | 9/self-report | stigma experience | + | + | n.a. | Mental health patients |  | |  | |  | |  | |  | | X |
| 66 | Stigma Impact Scale (SIS) | Fife BL & Wright ER (2000) [66] | - | 24/self-report | social rejection, financial insecurity, internalized shame, social isolation | + | + | n.a. | Dementia |  | |  | |  | |  | |  | | X |
| 67 | UCLA-3 Loneliness Scale (UCLA-3-LS) | Hughes ME et al. (2004) [67] | 20-item version (UCLA-20-LS; Russel, 1978) | 3/self-report | subjective feelings of loneliness | + | + | n.a. | Older population |  | |  | |  | |  | |  | | X |
| **No** | **Instrument**  **name** | **Author (First published)** | **Versions** | **No. items/data collec-tion** | **Key**  **thematic concepts** | **Psycho-**  **metrics** | | | **Designed**  **for** | **Social health**  **domain(s)*** | | | | | | | | | | |
|  |  |  |  |  |  | Reliability | Validity | Responsiveness |  | Cap. (A1) | | Ind. (A2) | | SocP. (A3) | | Str. (B1) | | Func. (B2) | | Appr. (B3) |
| 68 | Victoria Longitudinal Study (VLS) Activity Questionnaire | Hultsch DF et al. (1993) [68] | revised version (Hultsch et al. 1999); shorter version (Jopp & Hertzog (2007) | 56/self-report | physical, craft, games, TV, Social-private, social-public, religious, developmental, experiential, technology, travel | + | + | n.a. | Older population |  | |  | | X | |  | |  | |  |
| 69 | The German Version of the Maastricht Electronic Daily Life Observation Tool (MEDLO-tool) | de Boer et al., Dichter et al.  (2016) [69] | - | 32/caregiver report (proxy) | social interactions, no social interaction,  one-way interaction from the resident's perspective,  one-way interaction from someone else, two-way interaction, interactions with more than one person | + | + | n.a | Older population |  | |  | | X | | X | | X | |  |
| 70 | Observed Emotion Rating Scale (OERS) | Lawton, Van Haitsma, & Klapper (1999) [70] | - | 2/observation (proxy) | two positive emotions (pleasure and general alertness) and three negative emotions (anger, anxiety or fear, and sadness) | + | n.a. | n.a. | General population |  | |  | |  | |  | | X | |  |
| 71 | Ability Assessment of the older adult | Ministry of Civil Affairs of People's Republic of China [71] | - | 5/self-report | social participation: life ability, ability to work, time/space orientation, person orientation, and social interaction ability | + | n.a. | n.a. | Older population |  | | X | | X | |  | |  | |  |
| 72 | social engagement (SE) index | Bassuk et al.; Ellwardt et al. (1999) [72] | - | 12/self-report | social engagement | + | n.a | n.a | General population |  | |  | | X | |  | |  | |  |
| 73 | perceived stigma assessment tool | Piver et al. (2013) [73] | - | 8/self-report | perceived stigma | + | n.a | n.a | Older population |  | |  | |  | |  | |  | | X |
| **No** | **Instrument**  **name** | **Author (First published)** | **Versions** | **No. items/data collec-tion** | **Key**  **thematic concepts** | **Psycho-**  **metrics** | | | **Designed**  **for** | **Social health**  **domain(s)*** | | | | | | | | | | |
|  |  |  |  |  |  | Reliability | Validity | Responsiveness |  | Cap. (A1) | | Ind. (A2) | | SocP. (A3) | | Str. (B1) | | Func. (B2) | | Appr. (B3) |
| 74 | ten-item Family Orientation sub-scale of the Chinese Personality Assessment Inventory (CPAI-2) | Cheung et al.  (1996)[74] | - | 10/self-report | family cohesion | + | n.a | n.a | General population |  | |  | |  | |  | | X | | X |
| 75 | Berkman Syme Network Index (SNI) | Berkman and Syme  (1979) [75] | - | 11/self-report | marital status sociability, church group membership, and membership in other community organizations | + | + | n.a. | General population |  | |  | |  | | X | | X | |  |
| 76 | Perceived Social Isolation Scale | Cornwell et al.  (2009) [76] | - | 9/self-report | loneliness and perceived lack of social support from family, friends, and spouse or current partner | + | n.a. | n.a. | General population |  | |  | |  | |  | | X | | X |
| 77 | Inventory of Interpersonal Situations (IIS) | Van Dam-Baggen & Kraaimaat (1999)[77] | - | 35/self-report | specific everyday social situations | + | n.a. | n.a. | General population | X | |  | |  | |  | | X | | X |
| 78 | Apathy Motivation Index (AMI) | Ang et al. (2017) [78] | - | 18/self -report and carer report | patients with neurological conditions | + | + | n.a. | Patients with neurological conditions |  | | X | | X | |  | |  | |  |
| 79 | social isolation using a 15-item scale | Cornwell et al. (2009) [50] | - | 15/self-report | social connectedness, social participation, social support and loneliness | + | n.a. | n.a. | Older population |  | |  | | X | | X | | X | |  |
| **No** | **Instrument**  **name** | **Author (First published)** | **Versions** | **No. items/data collec-tion** | **Key**  **thematic concepts** | **Psycho-**  **metrics** | | | **Designed**  **for** | **Social health**  **domain(s)*** | | | | | | | | | | |
|  |  |  |  |  |  | Reliability | Validity | Responsiveness |  | Cap. (A1) | | Ind. (A2) | | SocP. (A3) | | Str. (B1) | | Func. (B2) | | Appr. (B3) |
| 80 | Family Stigma in Alzheimer’s Disease Scale (FS-ADS) | Werner, Goldstein et al. (2011)[79] | - | 19/self-report | Structural Discrimination, Negative Severity Attributions, Negative Aesthetic Attributions, Antipathy, Pity, Social Distance | + | + | n.a. | Dementia |  | |  | |  | |  | |  | | X |
| 81 | Social Support Rating Scale (SSRC) | Xiao, S. Y.  (1994) [80] | - | 10/self-report | objective support, subjective support, and support utilization | + | + | n.a. | General population |  | |  | |  | | X | | X | |  |
| 82 | Florida Cognitive Activities Scale (FCAS) | Schinka et al. (2005) [81] | - | 25/self-report | cognitive and social activities | + | + | n.a. | Older population |  | |  | | X | |  | |  | |  |
| 83 | The Positive Affect Index (PAI) | Bengtson & Schrader (1982) [45] | - | 5/self-report | closeness, communication, similarity of views, shared activities, generally getting along | + | n.a. | n.a. | Older population |  | |  | |  | |  | |  | | X |
| 84 | Aid for Decision ‐ Making in Occupation Choice (ADOC) | Levack, L; Tomori,K.; Takasashi, K., Sherrington, A. (2018)[82] | - | 95/self-report | self-care, mobilitiy, domestic life, work/education, interpersonal interaction, social life, sport, and leisure | + | + | n.a. | Older population with MCI | X | | X | | X | |  | |  | |  |
| 85 | 13-item version of the Marlowe-Crowne Social Desirability Scale | Reynolds et al. (1982) [30] | - | 13/self-report | Social desirability | + | n.a. | n.a. | General population |  | |  | |  | |  | | X | | X |
| **No** | **Instrument**  **name** | **Author (First published)** | **Versions** | **No. items/data collec-tion** | **Key**  **thematic concepts** | **Psycho-**  **metrics** | | | **Designed**  **for** | **Social health**  **domain(s)*** | | | | | | | | | | |
|  |  |  |  |  |  | Reliability | Validity | Responsiveness |  | Cap. (A1) | | Ind. (A2) | | SocP. (A3) | | Str. (B1) | | Func. (B2) | | Appr. (B3) |
| 86 | 12-items from - Healthy Aging Questionnaire | Canadian Community Health Survey (CCHS) (2008) [83] | - | 12/self-report | including membership in community organizations, participating in religious activities, being a volunteer, playing music, painting, visiting family members or friends, attending a community center, going to restaurants, libraries, shopping malls, cultural and sportive centers, and events | + | n.a | n.a. | Older population |  | |  | | X | |  | |  | |  |
| 87 | IMIAS social network scale | Ahmet, T.; Belanger, E.; et al. (2018) [84] | - | 4/self-report | the numbers of friends, living children, and extended family (i.e., grandchildren and siblings); (b) the numbers of those social ties that they see at least once a month; (c) that they have a close relationship with; and d) that they speak to by phone at least once a month | + | + | n.a. | Older population |  | |  | |  | | X | |  | | X |
| 88 | IMIAS’s social support scale | Ahmet, T.; Belanger, E.; et al. (2018)[84] | - | 5/self-report | whether participants felt helpful, loved, listened to, important to, and useful to their social ties, including friends, children, extended family, and partner | + | n.a. | n.a. | Older population |  | |  | |  | |  | | X | |  |
| 89 | Social Network Questionnaire 8SNQ) -27 item | Shaw et al.  (2007) [85] | - | 27/self-report | social embeddedness, Enacted Support, provided support,  perceived support | + | n.a. | n.a. | General population |  | |  | |  | |  | | X | |  |
| **No** | **Instrument**  **name** | **Author (First published)** | **Versions** | **No. items/data collec-tion** | **Key**  **thematic concepts** | **Psycho-**  **metrics** | | | **Designed**  **for** | **Social health**  **domain(s)*** | | | | | | | | | | |
|  |  |  |  |  |  | Reliability | Validity | Responsiveness |  | Cap. (A1) | | Ind. (A2) | | SocP. (A3) | | Str. (B1) | | Func. (B2) | | Appr. (B3) |
| 90 | dementia-related stigma scale | Blay, SL & Peluso, ETP  (2010) [86] | - | 3/self-report | stereotypes, prejudice, and discrimination | + | n.a. | n.a. | Middle-aged population |  | |  | |  | |  | |  | | X |
| 91 | ENRICHD Social Support Instrument | ENRICHD investigators  (2000) [16] | - | 7/self-report | emotional support, instrumental support, appraisal support, and marital status | + | + | n.a | General population |  | |  | |  | |  | | X | |  |
| 92 | Japanese version of the assessment scale of dementia stigma (ASDS) | Nogochi et al.  (2022) [87] | - | 3/self-report | anticipated stigma | + | + | n.a. | Older population with demented |  | |  | |  | |  | |  | | X |
| 93 | Japanese version of the Rosenberg Self-Esteem Scale (RSES-J) | Mimura & Griffiths (2007) [88] | - | 35/self-report | subject–staff interaction, environmental adjustment through collaboration, necessary information gathering and problem awareness, proactive behavioral practices, self-disclosure, and self-management of activities. | + | + | n.a. | Older population |  | |  | |  | |  | |  | | X |
| 94 | 6-item Life Engagement Test | Scheier et al.  (2006) [89] | - | 6/self-report | subjective view on one’s purpose of life | + | + | n.a. | General population |  | |  | |  | |  | |  | | X |
| 95 | Social functioning Scale specific to PD (PDSFS) | Morosini PL et al., Sommerlad A et al. (2000) [90] | - | 36/self-report | social activities, social support, social connection, interpersonal  communication, leisure activities, daily activities,  and occupational function | + | + | n.a. | General population with Parkinsons Diesease |  | | X | |  | |  | | X | |  |
| **No** | **Instrument**  **name** | **Author (First published)** | **Versions** | **No. items/data collec-tion** | **Key**  **thematic concepts** | **Psycho-**  **metrics** | | | **Designed**  **for** | **Social health**  **domain(s)*** | | | | | | | | | | |
|  |  |  |  |  |  | Reliability | Validity | Responsiveness |  | Cap. (A1) | | Ind. (A2) | | SocP. (A3) | | Str. (B1) | | Func. (B2) | | Appr. (B3) |
| 96 | Social Capital Scale to cover the two dimensions of social capital (social cohesion and social interaction) | Mujahid et al. (2007) [91] | - | 9/self-report | cohesion subscale, social interaction scale, | + | + | n.a. | Older population |  | |  | |  | | X | | X | |  |
| 97 | Practitioner Assessment of Network Type (PANT) | Wenger et al. (1994) [92] | - | 8/self-report | distance to nearest relative, child or sibling and contact frequency with children or other relatives, friends in the community/ neighbourhood, neighbours, religious involvement, and involvement in community or social groups | n.a. | + | n.a. | General population |  | |  | |  | | X | |  | |  |
| 98 | The social support and strain scales  Apathy Evaluation Scale-informant version (AES-I) | Marin et al. (1990) [93] | - | 8/self-report | social support and strain | + | n.a. | n.a. | General population |  | |  | |  | |  | | X | |  |
| 99 | Leisure Activities Questionnaire (LAQ) | Tinsley, H. E. A. and Kass, R. A (1980) [94] | - | 25/ carer report (proxy) | social activities, physical activities, intellectual activities, and recreational activities | + | + | n.a. | Older population (with care degree) |  | |  | | X | |  | |  | |  |
| **No** | **Instrument**  **name** | **Author (First published)** | **Versions** | **No. items/data collec-tion** | **Key**  **thematic concepts** | **Psycho-**  **metrics** | | | **Designed**  **for** | **Social health**  **domain(s)*** | | | | | | | | | | |
|  |  |  |  |  |  | Reliability | Validity | Responsiveness |  | Cap. (A1) | | Ind. (A2) | | SocP. (A3) | | Str. (B1) | | Func. (B2) | | Appr. (B3) |
| 100 | The perceived neighborhood physical disorder index and the perceived neighborhood social cohesion index | - | - | 4/self-report | feeling of belonging to this area, trust people, friendly people, and whether participants would get help from others if they were in trouble | + | n.a. | n.a. | Older population |  | |  | |  | |  | | X | |  |
| 101 | PD social functioning scale (PDSFS) | Su et al. (2020) [95] | - | 23/self-report | family life, hobbies, self-care, interpersonal relationships and recreational leisure, and social bond | + | + | n.a. | Older population |  | |  | |  | |  | | X | |  |
| 102 | “name generator” approach | Bidart C, Charbonneau J (2011) [96] | - | 6/self-report | social network size and structure | + | n.a. | n.a. | General population |  | |  | |  | | X | |  | |  |
| n.a. = not available; + = available  Social Health Domain(s)*: A1. The capacity to fulfill one’s potential (competencies) and obligations (social demands); A2. The ability to manage life with some degree of independence (despite a medical condition); A3. The ability to participate in social activities; B1. Structure/infrastructure (e.g., size, density, or types of relationships); B2. Functions served by an immediate network (e.g., emotional support, instrumental aid); B3. Appraisal of the quality of the relationship and interaction (e.g., relationship quality and satisfaction) | | | | | | | | | | | | | | | | | | | | |

References

1. Shakespeare-Finch J, Obst PL: **The development of the 2-Way Social Support Scale: a measure of giving and receiving emotional and instrumental support**. *J Pers Assess* 2011, **93**(5):483-490.

2. Maxfield M, Greenberg J: **Anticipated Stigma and Dementia-Related Anxiety in Middle-Aged and Older Adults**. *GeroPsych (Bern)* 2021, **34**(1):13-22.

3. Barrera MJ: **A method for the assessment of social support networks in community survey research**. In*.*, vol. 3: Connection; 1980: 8-13.

4. Krause N, Rook KS: **Negative interaction in late life: issues in the stability and generalizability of conflict across relationships**. *J Gerontol B Psychol Sci Soc Sci* 2003, **58**(2):P88-99.

5. Elwyn G, Barr PJ, Grande SW, Thompson R, Walsh T, Ozanne EM: **Developing CollaboRATE: a fast and frugal patient-reported measure of shared decision making in clinical encounters**. *Patient Educ Couns* 2013, **93**(1):102-107.

6. Zawisza K, Galas A, Tobiasz-Adamczyk B, Chatterji S, Haro JM, Miret M, Koskinen S, Power M, Leonardi M: **The validity of the instrument to evaluate social network in the ageing population: the Collaborative Research on Ageing in Europe Social Network Index**. *Clin Psychol Psychother* 2014, **21**(3):227-241.

7. Marson DC, Ingram KK, Cody HA, Harrell LE: **Assessing the competency of patients with Alzheimer's disease under different legal standards. A prototype instrument**. *Arch Neurol* 1995, **52**(10):949-954.

8. Appelbaum PS, Bonnie RJ, Karlawish JH: **The capacity to vote of persons with Alzheimer's disease**. *Am J Psychiatry* 2005, **162**(11):2094-2100.

9. O'Connor AM: **Validation of a decisional conflict scale**. *Med Decis Making* 1995, **15**(1):25-30.

10. Feinberg LW, CJ **Making Hard Choices. Respecting Both Voices**. In*.* San Francisco: Family Caregiver Alliance; 2000.

11. De Jong Gierveld J, van Tilburg, T: **A 6-Item Scale for Overall, Emotional, and Social Loneliness**. *Research on Aging* 2006, **28(5)**:582-598.

12. Larzelere R, Huston T: **The dyadic trust scale: Toward understanding interpersonal trust in close relationships**. *Journal of Marriage and the Family* 1980:595-604.

13. Broadhead WE, Gehlbach SH, de Gruy FV, Kaplan BH: **The Duke-UNC Functional Social Support Questionnaire. Measurement of social support in family medicine patients**. *Med Care* 1988, **26**(7):709-723.

14. Koenig HG, Westlund RE, George LK, Hughes DC, Blazer DG, Hybels C: **Abbreviating the Duke Social Support Index for use in chronically ill elderly individuals**. *Psychosomatics* 1993, **34**(1):61-69.

15. Stoner CR, Orrell M, Spector A: **Psychometric Properties and Factor Analysis of the Engagement and Independence in Dementia Questionnaire (EID-Q)**. *Dement Geriatr Cogn Disord* 2018, **46**(3-4):119-127.

16. **Enhancing recovery in coronary heart disease patients (ENRICHD): study design and methods. The ENRICHD investigators**. *Am Heart J* 2000, **139**(1 Pt 1):1-9.

17. Resnick B, Gruber-Baldini AL, Pretzer-Aboff I, Galik E, Buie VC, Russ K, Zimmerman S: **Reliability and validity of the evaluation to sign consent measure**. *Gerontologist* 2007, **47**(1):69-77.

18. H SCGFPHJJCTLMS: **Development of the Family Emotional Involvement and Criticism Scales (FEICS): A Self-Report Scale to Measure Expressed Emotion**. *Journal of Marital and Family Therapy* 1992, **18(4)**:395-407.

19. Hawthorne G: **Measuring Social Isolation in Older Adults: Development and Initial Validation of the Friendship Scale**. *Soc Indic Res* 2006, **77**:521-548.

20. Fydrich T, Sommer, G., Tydecks, S., Brähler, E.: **Social Support Questionnaire (F-SouU): Standardization of short form (K-14).** *Zeitschrift fur Medizinische Psychologie* 2009, **18**:43-48.

21. Gagnon M, Hibert R, Dube M, Dubois MF: **Development and validation of an instrument measuring individual empowerment in relation to personal health care: the Health Care Empowerment Questionnaire (HCEQ)**. *Am J Health Promot* 2006, **20**(6):429-435.

22. Hertz JEG: **The perceived enactment of autonomy scale: Measuring the potential for self-care action in the elderly**. *The University of Texas at Austin* 1991.

23. Phillips SP, Auais M, Belanger E, Alvarado B, Zunzunegui MV: **Life-course social and economic circumstances, gender, and resilience in older adults: The longitudinal International Mobility in Aging Study (IMIAS)**. *SSM Popul Health* 2016, **2**:708-717.

24. Mor V, Branco K, Fleishman J, Hawes C, Phillips C, Morris J, Fries B: **The structure of social engagement among nursing home residents**. *J Gerontol B Psychol Sci Soc Sci* 1995, **50**(1):P1-P8.

25. Cohen S, & Hoberman, H. M.: **Positive events and social supports as buffers of life change stress**. *Journal of applied social psychology* 1983, **13(2)**:99-125.

26. Henderson S, Duncan-Jones P, Byrne DG, Scott R: **Measuring social relationships. The Interview Schedule for Social Interaction**. *Psychol Med* 1980, **10**(4):723-734.

27. Tallberg IM, Stormoen S, Almkvist O, Eriksdotter M, Sundstrom E: **Investigating medical decision-making capacity in patients with cognitive impairment using a protocol based on linguistic features**. *Scand J Psychol* 2013, **54**(5):386-392.

28. Lubben J, Blozik E, Gillmann G, Iliffe S, von Renteln Kruse W, Beck JC, Stuck AE: **Performance of an abbreviated version of the Lubben Social Network Scale among three European community-dwelling older adult populations**. *Gerontologist* 2006, **46**(4):503-513.

29. Appelbaum PS, & Grisso, T.: **MacArthur competence assessment tool for clinical research (MacCAT-CR)**. *Professional Resource Press/Professional Resource Exchange* 2001.

30. Reynolds WM: **Development of reliable and valid short forms of the marlowe-crowne social desirability scale.Psychodiagnostic Processes: Personality Inventories and Scales**. *Journal of Clinical Psychology* 1982, **38(1)**:119-125.

31. Sherbourne CD, Stewart AL: **The MOS social support survey**. *Soc Sci Med* 1991, **32**(6):705-714.

32. Mann L, Burnett, P., Radford, M., & Ford, S: **The Melbourne Decision Making Questionnaire: An instrument for measuring patterns for coping with decisional conflict**. *Journal of Behavioral Decision Making* 1997, **10(1)**:1-19.

33. Morris JN, Hawes C, Fries BE, Phillips CD, Mor V, Katz S, Murphy K, Drugovich ML, Friedlob AS: **Designing the national resident assessment instrument for nursing homes**. *Gerontologist* 1990, **30**(3):293-307.

34. Boyle PA, Yu L, Wilson RS, Segawa E, Buchman AS, Bennett DA: **Cognitive decline impairs financial and health literacy among community-based older persons without dementia**. *Psychol Aging* 2013, **28**(3):614-624.

35. Zimet GD, Dahlem, N. W., Zimet, S. G., & Farley, G. K: **The multidimensional scale of perceived social support**. *Journal of personality assessment* 1988, **52(1)**:30-41.

36. Dalgard OS, Dowrick C, Lehtinen V, Vazquez-Barquero JL, Casey P, Wilkinson G, Ayuso-Mateos JL, Page H, Dunn G, Group O: **Negative life events, social support and gender difference in depression: a multinational community survey with data from the ODIN study**. *Soc Psychiatry Psychiatr Epidemiol* 2006, **41**(6):444-451.

37. Hahlweg K: **Fragebogen zur Partnerschaftsdiagnostik (FDP) [Partnership Questionnaire (PFB)]**. Göttingen, Germany: Hogrefe; 1996.

38. Colling KB: **A taxonomy of passive behaviors in people with Alzheimer's disease**. *J Nurs Scholarsh* 2000, **32**(3):239-244.

39. Chochinov HM, Hassard T, McClement S, Hack T, Kristjanson LJ, Harlos M, Sinclair S, Murray A: **The patient dignity inventory: a novel way of measuring dignity-related distress in palliative care**. *J Pain Symptom Manage* 2008, **36**(6):559-571.

40. Weinert C: **Social support in cyberspace for women with chronic illness**. *Rehabilitation Nursing* 2000, **25(4)**.

41. Wood-Dauphinee SL, Opzoomer MA, Williams JI, Marchand B, Spitzer WO: **Assessment of global function: The Reintegration to Normal Living Index**. *Arch Phys Med Rehabil* 1988, **69**(8):583-590.

42. Chiu YC, Huang CY, Kolanowski AM, Huang HL, Shyu YL, Lee SH, Lin CR, Hsu WC: **The effects of participation in leisure activities on neuropsychiatric symptoms of persons with cognitive impairment: a cross-sectional study**. *Int J Nurs Stud* 2013, **50**(10):1314-1325.

43. Gerritsen DL, Steverink N, Frijters DH, Hirdes JP, Ooms ME, Ribbe MW: **A revised Index for Social Engagement for long-term care**. *J Gerontol Nurs* 2008, **34**(4):40-48.

44. Logsdon RG, Teri L: **The Pleasant Events Schedule-AD: psychometric properties and relationship to depression and cognition in Alzheimer's disease patients**. *Gerontologist* 1997, **37**(1):40-45.

45. Bengtson VLS, S.S: **Parents-Child Relations**. In: *Research Instruments in Social Gerontology.* Edited by Mangen DJaP, W.A, vol. 2. Minneapolis, USA: University of Minnesota Press; 1982: 115-128.

46. Holmes-Rovner M, Kroll J, Schmitt N, Rovner DR, Breer ML, Rothert ML, Padonu G, Talarczyk G: **Patient satisfaction with health care decisions: the satisfaction with decision scale**. *Med Decis Making* 1996, **16**(1):58-64.

47. Spruytte N VAC, Lammertyn F: **The Scale for the Quality of the Current Relationship in Caregiving**. *Internal Report LUCAS-KULeuven* 2000.

48. Bosc M, Dubini A, Polin V: **Development and validation of a social functioning scale, the Social Adaptation Self-evaluation Scale**. *Eur Neuropsychopharmacol* 1997, **7 Suppl 1**:S57-70; discussion S71-53.

49. Harvey PD, Davidson M, Mueser KT, Parrella M, White L, Powchik P: **Social-Adaptive Functioning Evaluation (SAFE): a rating scale for geriatric psychiatric patients**. *Schizophr Bull* 1997, **23**(1):131-145.

50. Cornwell EY, Waite LJ: **Measuring social isolation among older adults using multiple indicators from the NSHAP study**. *J Gerontol B Psychol Sci Soc Sci* 2009, **64 Suppl 1**(Suppl 1):i38-46.

51. Link BG, Cullen, F. T., Frank, J., & Wozniak, J. F: **The social rejection of former mental patients: Understanding why labels matter**. *American journal of Sociology* 1987, **92(6)**:1461-1500.

52. Sommerlad A, Singleton D, Jones R, Banerjee S, Livingston G: **Development of an instrument to assess social functioning in dementia: The Social Functioning in Dementia scale (SF-DEM)**. *Alzheimers Dement (Amst)* 2017, **7**:88-98.

53. Chen Y-L RM, Feldt K, Savik K: **The relationship between social interaction and characteristics of aggressive, cognitively impaired nursing home residents**. *American Journal of Alzheimer’s Disease* 2000, **15(1)**:10-17.

54. Rankin KP: **Social Norms Questionaire NINDS Domain Specific Tasks of Executive Function. Executive abilities: measures and instruments for neurobehavioral evaluation and research (EXAMINER) user manual**. 2008, **3**:45-47.

55. Mabire JB, Gay MC, Vrignaud P, Garitte C, Vernooij-Dassen M: **Social interactions between people with dementia: pilot evaluation of an observational instrument in a nursing home**. *Int Psychogeriatr* 2016, **28**(6):1005-1015.

56. Lowe MR, & Cautela, J. R: **A self-report measure of social skill**. *Behavior Therapy* 1978, **9(4)**:535-544.

57. D’Zurilla TJ, Nezu, A. M., & Maydeu-Olivares, A: **Social problem-solving inventory-revised**. 2002.

58. Cutrona CE, & Russell, D. W: **The provisions of social relationships and adaptation to stress**. *Advances in personal relationships* 1987, **1(1)**:37-67.

59. Jette AM, Haley SM, Coster WJ, Kooyoomjian JT, Levenson S, Heeren T, Ashba J: **Late life function and disability instrument: I. Development and evaluation of the disability component**. *J Gerontol A Biol Sci Med Sci* 2002, **57**(4):M209-216.

60. Sarason IG, Sarason, B. R., Shearin, E. N., & Pierce, G. R. : **A Brief Measure of Social Support: Practical and Theoretical Implications**. *Journal of Social and Personal Relationships* 1987, **4(4)**:497–510.

61. Lue BH, Leung, K.K., Fan-Jiang, C.S., Chen, H.J: **A study of social support, family interaction in relation to mental health**. *Chin J Fam Med* 1995, **15**:173-182.

62. Pinsker DM, McFarland K: **Exploitation in older adults: personal competence correlates of social vulnerability**. *Neuropsychol Dev Cogn B Aging Neuropsychol Cogn* 2010, **17**(6):673-708.

63. Barsuglia JP, Kaiser NC, Wilkins SS, Karve SJ, Barrows RJ, Paholpak P, Panchal HV, Jimenez EE, Mather MJ, Mendez MF: **A scale of socioemotional dysfunction in frontotemporal dementia**. *Arch Clin Neuropsychol* 2014, **29**(8):793-805.

64. Bramham J, Morris RG, Hornak J, Bullock P, Polkey CE: **Social and emotional functioning following bilateral and unilateral neurosurgical prefrontal cortex lesions**. *J Neuropsychol* 2009, **3**(Pt 1):125-143.

65. Wahl OF: **Mental health consumers' experience of stigma**. *Schizophr Bull* 1999, **25**(3):467-478.

66. Fife BL, Wright ER: **The dimensionality of stigma: a comparison of its impact on the self of persons with HIV/AIDS and cancer**. *J Health Soc Behav* 2000, **41**(1):50-67.

67. Hughes ME, Waite LJ, Hawkley LC, Cacioppo JT: **A Short Scale for Measuring Loneliness in Large Surveys: Results From Two Population-Based Studies**. *Res Aging* 2004, **26**(6):655-672.

68. Hultsch DF, Hammer M, Small BJ: **Age differences in cognitive performance in later life: relationships to self-reported health and activity life style**. *J Gerontol* 1993, **48**(1):P1-11.

69. de Boer B, Hamers JPH, Zwakhalen SMG, Tan FES, Verbeek H: **Quality of care and quality of life of people with dementia living at green care farms: a cross-sectional study**. *BMC Geriatr* 2017, **17**(1):155.

70. Lawton MP: **Quality of Life in Alzheimer Disease**. *ALZHEIMER DIS ASSOC DISORD (Alzheimer Disease & Associated Disorders)* 1994, **8**.

71. Wang S, Feng X, Liu Y, Wang H, Xie H, Wang Z: **Ability assessment for older adults. Ministry of Civil Affairs of the People's Republic of China**. 2013.

72. Bassuk SS, Glass, T. A., & Berkman, L. F.: **Social disengagement and incident cognitive decline in community-dwelling elderly persons**. *Annals of Internal Medicine* 1999, **131(3)**:165-173.

73. Piver LC, Nubukpo P, Faure A, Dumoitier N, Couratier P, Clement JP: **Describing perceived stigma against Alzheimer's disease in a general population in France: the STIG-MA survey**. *Int J Geriatr Psychiatry* 2013, **28**(9):933-938.

74. Cheung F, Leung K, Fan R, Zhang J-P: **Development of the Chinese Personality Assessment Inventory (CPAI)**. *Journal of Cross-Cultural Psychology* 1996, **27(2)**:181-199.

75. Berkman LF SS: **Social networks, host resistance, and mortality: a nine-year follow-up study of Alameda County residents**. *Am J Epidemiol* 1979, **109(2)**:186-204.

76. Cornwell EY, Waite LJ: **Social disconnectedness, perceived isolation, and health among older adults**. *J Health Soc Behav* 2009, **50**(1):31-48.

77. van Dam-Baggen R, & Kraaimaat, F: **Assessing social anxiety: The Inventory of Interpersonal Situations (IIS)**. *European Journal of Psychological Assessment* 1999:25-38.

78. Ang YS, Lockwood P, Apps MA, Muhammed K, Husain M: **Distinct Subtypes of Apathy Revealed by the Apathy Motivation Index**. *PLoS One* 2017, **12**(1):e0169938.

79. Werner P, Goldstein D, Heinik J: **Development and validity of the Family Stigma in Alzheimer's disease Scale (FS-ADS)**. *Alzheimer Dis Assoc Disord* 2011, **25**(1):42-48.

80. Xiao S: **The theoretical basis and applications of Social Support Rating Scale (SSRS)**. *J Clinical Psychiatry* 1994, **02**:98-100.

81. Schinka JA, McBride A, Vanderploeg RD, Tennyson K, Borenstein AR, Mortimer JA: **Florida Cognitive Activities Scale: initial development and validation**. *J Int Neuropsychol Soc* 2005, **11**(1):108-116.

82. Levack W, Tomori K, Takahashi K, Sherrington AJ: **Development of an English-language version of a Japanese iPad application to facilitate collaborative goal setting in rehabilitation: a Delphi study and field test**. *BMJ Open* 2018, **8**(3):e018908.

83. Statistics C: **Canadian Community Health Survey (CCHS)- Healthy Aging Questionnaire (2008–2009)**. *Statistics Canada* 2010:117-120.

84. Ahmed T, Belanger E, Vafaei A, Kone GK, Alvarado B, Beland F, Zunzunegui MV, Group IR: **Validation of a Social Networks and Support Measurement Tool for Use in International Aging Research: The International Mobility in Aging Study**. *J Cross Cult Gerontol* 2018, **33**(1):101-120.

85. Shaw B, Krause, N., Liang, J., & Bennett, J.: **Tracking change in social relations throughout late life**. *Journal of Gerontology: Social Sciences* 2007, **62(2)**:90-99.

86. Blay SL, Toledo Pisa Peluso E: **Public stigma: the community's tolerance of Alzheimer disease**. *Am J Geriatr Psychiatry* 2010, **18**(2):163-171.

87. Noguchi T, Shang E, Nakagawa T, Komatsu A, Murata C, Saito T: **Establishment of the Japanese version of the dementia stigma assessment scale**. *Geriatr Gerontol Int* 2022, **22**(9):790-796.

88. Mimura C, Griffiths P: **A Japanese version of the Rosenberg Self-Esteem Scale: translation and equivalence assessment.** *J Psychosom Res* 2007, **62(5)**:589-594.

89. Scheier MF, Wrosch C, Baum A, Cohen S, Martire LM, Matthews KA, Schulz R, Zdaniuk B: **The Life Engagement Test: assessing purpose in life**. *J Behav Med* 2006, **29**(3):291-298.

90. Morosini PL ML, Brambilla L, Ugolini S, Pioli **Development, reliability and acceptability of a new version of the DSM-IV Social and Occupational Functioning Assessment Scale (SOFAS) to assess routine social funtioning.** *Acta Psychiatr Scand* 2000, **101**:323-329.

91. Mujahid MS DRA, Morenoff JD, Raghunathan T: **Assessing the measurement properties of neighborhood scales: from psychometrics to ecometrics**. *Am J Epidemiol* 2007:858-867.

92. Wenger G: **Support network of older people: a guide for practitioners**. *Centre for Social Policy Research and Development University College of Wales* 1994.

93. Marin R: **Differential diagnosis and classification of apathy**. *Am J Psychiatry* 1990, **147(1)**:22-30.

94. Tinsley HEA, & Kass, R. A: **Discriminant Validity of the Leisure Activity Questionnaire and the Paragraphs About Leisure**. *Educational and Psychological Measurement* 1980, **40(1)**:227-233.

95. Su FT, Tai CH, Tan CH, Hwang WJ, Yu RL: **The Development of the Social Functioning Scale for Patients with Parkinson's Disease**. *J Parkinsons Dis* 2020, **10**(3):1143-1151.

96. Bidart C, & Charbonneau, J. : **How to generate personal networks: Issues and tools for a sociological perspective**. *Field Methods* 2011, **23(3)**:266-286.
